# Diagnostic performance and clinical impact of the BioFire FilmArray Pneumonia Panel compared with conventional culture in hospitalized patients with pneumonia

**DOI:** 10.1371/journal.pone.0358986

**Published:** 2026-09-28

**Authors:** I. Re Heo, Sunjoo Kim

**Affiliations:** 1 Department of Internal Medicine, Gyeongsang National University College of Medicine, Jinju, Republic of Korea; 2 Department of Laboratory Medicine, GC Labs, Yangsan, Republic of Korea; Università degli Studi di Parma: Universita degli Studi di Parma, ITALY

## Abstract

**Background:**

Rapid and accurate identification of respiratory pathogens and antimicrobial resistance is essential for optimizing antimicrobial therapy in pneumonia. Conventional culture-based diagnostics are limited by delayed turnaround times and reduced sensitivity, particularly after prior antibiotic exposure. The BioFire FilmArray Pneumonia Panel enables rapid multiplex detection of respiratory pathogens and antimicrobial resistance genes directly from clinical specimens.

**Objective:**

To evaluate the diagnostic performance and clinical impact of the BioFire FilmArray Pneumonia Panel in hospitalized patients with pneumonia.

**Methods:**

We conducted a retrospective observational study comparing the diagnostic performance of the FilmArray Pneumonia Panel with conventional culture using 558 bronchoalveolar lavage and sputum specimens collected between December 2022 and December 2023. Diagnostic performance, pathogen detection, antimicrobial resistance genes, and polymicrobial detection patterns were evaluated. The clinical impact of FilmArray implementation was also assessed in 494 hospitalized patients by comparing antibiotic management and clinical outcomes between the FilmArray period and pre-implementation period (January to November 2022). Multivariable regression analyses were performed to adjust for baseline differences.

**Results:**

The FilmArray panel detected a broad spectrum of bacterial pathogens, respiratory viruses, and antimicrobial resistance genes, with a substantial proportion of specimens being culture-negative but FilmArray-positive, particularly sputum specimens. Compared with the pre-implementation period, the FilmArray period showed a significantly higher frequency of result-driven antibiotic actions (40.5% vs. 26.5%; adjusted odds ratio, 1.86; 95% confidence interval [CI], 1.24–2.79; P = 0.003) and a shorter time from microbiological result reporting to antibiotic action (adjusted mean difference, −0.59 days; 95% CI, −1.07 to −0.10; P = 0.019). Measured from specimen sampling, the interval to the antibiotic action was more than three days shorter (adjusted mean difference, −3.53 days; 95% CI, −4.08 to −2.99; P < 0.001), reflecting the faster turnaround of the panel (median, 0 vs. 3 days). Hospital length of stay was also shorter (adjusted mean difference, −1.61 days; 95% CI, −3.07 to −0.15; P = 0.031). However, total antibiotic exposure was modestly longer (adjusted mean difference, 1.86 days; 95% CI, 0.29–3.44; P = 0.021), and antibiotic de-escalation rates did not differ significantly between the two periods.

**Conclusions:**

The BioFire FilmArray Pneumonia Panel improved pathogen detection and facilitated earlier and more frequent microbiology-guided antibiotic management, and was associated with a modest reduction in hospital length of stay. However, it modestly increased overall antibiotic exposure and did not increase antibiotic de-escalation. Rapid molecular diagnostics may improve the timeliness and precision of antimicrobial therapy, but their full clinical benefit is likely to be realized only when they are used as a complementary tool alongside conventional microbiological methods and integrated within a structured antimicrobial stewardship program.

## Introduction

Lower respiratory tract infections (LRTIs), including pneumonia, remain a major cause of morbidity and mortality worldwide across all age groups [1]. Prompt identification of causative pathogens is essential for initiating appropriate antimicrobial therapy and improving clinical outcomes. However, conventional culture-based diagnostic methods are limited by prolonged turnaround times, reduced sensitivity following prior antibiotic exposure, and suboptimal detection of fastidious or atypical organisms [2,3].

Pneumonia can be classified as community-acquired pneumonia (CAP), hospital-acquired pneumonia (HAP), and ventilator-associated pneumonia (VAP), and may be caused by a wide variety of bacterial, viral, and atypical pathogens. The increasing prevalence of multidrug-resistant (MDR) organisms has further complicated empirical treatment strategies, underscoring the importance of rapid detection of antimicrobial resistance (AMR) determinants [2,4]. In addition, conventional phenotypic antimicrobial susceptibility testing generally requires 48–96 hours, often delaying appropriate initiation, escalation, or de-escalation of antimicrobial therapy [5].

Multiplex polymerase chain reaction (PCR)-based syndromic diagnostic platforms have emerged as promising tools to overcome the limitations of conventional microbiological methods. Among these, the BioFire FilmArray Pneumonia Panel (BioFire Diagnostics, Salt Lake City, UT) enables simultaneous detection of multiple respiratory pathogens as well as AMR genes directly from respiratory specimens within approximately one hour [4,6]. Previous multicenter studies have demonstrated that the FilmArray Pneumonia Panel provides higher sensitivity than conventional culture while substantially shortening the time to pathogen identification [4,6,7].

Beyond improving diagnostic accuracy, rapid molecular testing has the potential to influence downstream clinical management by enabling earlier microbiology-guided antibiotic decisions and supporting antimicrobial stewardship interventions [8]. Delayed pathogen identification contributes to prolonged empirical broad-spectrum antibiotic use, which is associated with adverse drug events, antimicrobial resistance, prolonged hospitalization, and mortality [9]. Although several studies have shown that rapid molecular diagnostics facilitate earlier antibiotic modification, their effects on overall antibiotic exposure, antibiotic de-escalation, and clinical outcomes have been inconsistent [5,8,10,11]. Consequently, real-world evidence regarding the clinical impact of multiplex molecular diagnostics remains limited.

Despite these advantages, concerns remain regarding the relatively lower specificity of molecular diagnostic panels because they may detect colonizing organisms or nonviable bacteria [6]. Therefore, molecular test results should be interpreted in conjunction with clinical, microbiological, and radiologic findings. Furthermore, most previous studies have focused primarily on intensive care unit (ICU) populations [12–14] or endotracheal aspirate specimens [13,15], whereas relatively few real-world studies have directly compared bronchoalveolar lavage (BAL) and sputum specimens using multiplex molecular assays [7,16].

This study evaluated the diagnostic performance and clinical utility of the BioFire FilmArray Pneumonia Panel using both BAL and sputum specimens from hospitalized patients with pneumonia. Specifically, we evaluated pathogen distribution, antimicrobial resistance gene detection, polymicrobial infection patterns, and diagnostic performance compared with conventional culture. We also assessed the clinical impact of FilmArray implementation by comparing result-driven antibiotic actions, diagnostic turnaround time, the time from specimen sampling and microbiological result reporting to antibiotic action, total antibiotic days, hospital length of stay, and 30-day mortality between the pre-implementation and FilmArray periods.

## Materials and methods

### Study design and specimens

This retrospective observational study consisted of two complementary analyses: a diagnostic-performance analysis and a clinical-impact analysis. The study was conducted in accordance with the principles of the Declaration of Helsinki and was approved by the Institutional Review Board (IRB) of Gyeongsang National University Changwon Hospital (IRB No. 2023-07-028). Given the retrospective study design and the use of de-identified patient data, the requirement for informed consent was waived by the IRB.

For the diagnostic-performance analysis, hospitalized patients with suspected pneumonia who underwent conventional microbiological culture and the BioFire FilmArray Pneumonia Panel between December 1, 2022, and December 31, 2023, were included. A total of 558 respiratory specimens were analyzed, comprising 297 bronchoalveolar lavage (BAL) specimens and 261 sputum specimens. Because some patients contributed more than one respiratory specimen, diagnostic performance was evaluated on a specimen basis rather than a patient basis.

All specimens were tested using the BioFire FilmArray Pneumonia Panel (BioFire Diagnostics, Salt Lake City, UT, USA) in accordance with the manufacturer’s instructions. The FilmArray Pneumonia Panel detects 15 bacterial pathogens, 8 respiratory viruses, 3 atypical bacteria, and 7 antimicrobial resistance genes directly from respiratory specimens using a fully automated multiplex PCR platform.

Conventional bacterial culture was performed according to standard laboratory procedures. BAL and sputum specimens were inoculated onto blood agar, MacConkey agar, and chocolate agar supplemented with vancomycin, bacitracin, and clindamycin (Asan Pharmaceutical, Seoul, Korea). The culture plates were incubated overnight at 37°C in 5% CO_2_. Bacterial isolates were identified using matrix-assisted laser desorption/ionization time-of-flight mass spectrometry (MALDI-TOF MS), and antimicrobial susceptibility testing was performed using the VITEK 2 system (bioMérieux, Marcy-l'Étoile, France).

Viral and atypical bacterial pathogens (*Legionella pneumophila*, *Chlamydia pneumoniae*, and *Mycoplasma pneumoniae*) are not routinely detected by conventional culture and were therefore excluded from direct comparisons between the FilmArray Pneumonia Panel and conventional culture. Accordingly, diagnostic performance analyses were performed for bacterial pathogens only.

### Clinical data collection

The clinical impact of FilmArray implementation was evaluated by comparing two consecutive study periods at Gyeongsang National University Changwon Hospital (GNUCH), a secondary referral hospital in Changwon, Republic of Korea. The FilmArray period comprised patients admitted between December 1, 2022, and December 31, 2023, whereas the pre-implementation period comprised patients admitted between January 1, 2022, and November 30, 2022, before FilmArray implementation. Hospitalized patients diagnosed with pneumonia based on compatible clinical manifestations and radiologic findings who received antibiotic treatment and had a positive microbiological result were eligible for the clinical-impact analysis. Patients with negative microbiological results were excluded. Clinical data, including demographic characteristics, microbiological findings, antibiotic use, and clinical outcomes, were retrospectively extracted from the electronic medical records. A total of 494 patients were included in the clinical-impact analysis, comprising 200 patients in the FilmArray period and 294 patients in the pre-implementation period (Supplementary Fig. 1). Baseline demographic and clinical characteristics were collected to assess the comparability of the two study periods.

The primary clinical-impact outcome was result-driven antibiotic action, defined as initiation of a new antibiotic or modification of an existing antibiotic regimen on the day of, or within 5 days after, reporting of the final microbiological result. Antibiotic modifications included both escalation and de-escalation of therapy. Escalation was defined as either changing to an antimicrobial regimen with a broader spectrum than the current regimen or adding one or more antimicrobial agents. De-escalation was defined as changing to a narrower-spectrum regimen or discontinuing one or more agents from combination therapy without increasing the overall antimicrobial spectrum. Turnaround time was defined as the interval from specimen sampling to reporting of the final microbiological result, and the time from sampling to antibiotic action as the interval from sampling to the qualifying result-driven action; turnaround time was compared without covariate adjustment because it is a property of the diagnostic method rather than of the patient. Secondary outcomes included total antibiotic days during hospitalization, hospital length of stay, and 30-day mortality.

### Statistical analysis

Continuous variables were summarized as mean ± standard deviation (SD) or median (interquartile range [IQR]), and categorical variables as frequencies and percentages. Between-group comparisons were performed using Welch’s t test or the Mann–Whitney U test for continuous variables and the chi-square test or Fisher’s exact test for categorical variables, as appropriate. Baseline balance before and after propensity score weighting was assessed using absolute standardized mean differences (SMDs).

To account for baseline imbalances between the two study groups, multivariable regression analyses were performed adjusting for age, sex, intensive care unit (ICU) admission, C-reactive protein (CRP), Eastern Cooperative Oncology Group (ECOG) performance status, and residence type. These covariates were selected because they either demonstrated SMDs of ≥0.10 with complete data (age, CRP, and residence type) or were prespecified a priori because of their established prognostic importance in patients with pneumonia (sex, ICU admission, and ECOG performance status). Continuous outcomes were analyzed using multivariable linear regression with HC3 heteroscedasticity-consistent standard errors, whereas binary outcomes were analyzed using multivariable logistic regression.

Residence type was collapsed from four categories to three (home; long-term care, comprising nursing homes and long-term care facilities; and acute-care hospital) because the nursing-home category contained only two patients in the entire cohort. Retaining this category as a separate model term resulted in a leverage value approaching 1 and unstable parameter estimates.

Propensity score weighting using stabilized inverse probability of treatment weights (IPTW), truncated at the 99th percentile, was performed as a sensitivity analysis. Covariate balance after weighting was assessed using SMDs.

Erythrocyte sedimentation rate, procalcitonin, and D-dimer also showed SMDs of ≥0.10 but had 6–8% missing values, predominantly in the pre-implementation period. These variables were therefore excluded from the prespecified primary models because their inclusion would have reduced the analysis population on the basis of incomplete laboratory data rather than clinical criteria. An exploratory model additionally adjusting for D-dimer was also fitted and is presented for completeness.

Normality was assessed using the Shapiro–Wilk test and quantile–quantile (Q–Q) plots. All continuous outcomes were substantially right-skewed (Shapiro–Wilk P < 0.001 in both groups for every outcome); therefore, unadjusted comparisons are presented using both the Welch t test and the Mann–Whitney U test.

As prespecified, antibiotic courses exceeding 45 days and hospital stays exceeding 30 days were excluded from the corresponding analyses. The numbers of excluded patients are reported in the footnotes to Table 6, and analyses without these restrictions are presented as sensitivity analyses. Patients who died during hospitalization were retained in the length-of-stay analysis, with length of stay defined as the interval from admission to death. All analyses were considered exploratory, and no adjustment was made for multiple comparisons. For the diagnostic performance analysis, conventional microbiological culture served as the reference standard. For each bacterial target, sensitivity, specificity, positive predictive value (PPV), negative predictive value (NPV), positive percent agreement (PPA), and negative percent agreement (NPA) were calculated. Effect estimates are presented with corresponding 95% confidence intervals whenever applicable. All eligible patients during the study period were included; no formal sample size calculation was performed because of the retrospective observational design.

All statistical analyses were performed using R software (version 4.4.2; R Foundation for Statistical Computing, Vienna, Austria). All tests were two-sided, and P < 0.05 was considered statistically significant.

## Results

### Pathogen distribution detected by the FilmArray pneumonia panel

Among the 558 respiratory specimens analyzed using the FilmArray Pneumonia Panel, 359 (64.3%) were positive for at least one pathogen, whereas 199 (35.7%) were negative (Table 1).

**Table 1:** Pathogens detected by the FilmArray Pneumonia Panel in bronchoalveolar lavage (BAL) and sputum specimens.

Pathogen	BAL N	Sputum N	Total N (%)
**Bacteria**			
*Acinetobacter calcoaceticus–baumannii* complex	30	22	52 (8.7)
*Enterobacter cloacae* complex	4	6	10 (1.7)
*Escherichia coli*	20	28	48 (8.1)
*Haemophilus influenzae*	29	44	73 (12.3)
*Klebsiella aerogenes*	12	3	15 (2.5)
*Klebsiella oxytoca*	3	5	8 (1.3)
*Klebsiella pneumoniae* group	59	52	111 (18.7)
*Moraxella catarrhalis*	0	4	4 (0.7)
*Proteus* spp.	16	12	28 (4.7)
*Pseudomonas aeruginosa*	54	37	91 (15.3)
*Serratia marcescens*	7	4	11 (1.8)
*Staphylococcus aureus*	42	43	85 (14.3)
*Streptococcus agalactiae*	11	13	24 (4.0)
*Streptococcus pneumoniae*	16	19	35 (5.9)
Total	303	292	595 (100.0)
**Atypical Bacteria**			
*Chlamydia pneumoniae*	1	0	1 (10.0)
*Legionella pneumophila*	5	1	6 (60.0)
*Mycoplasma pneumoniae*	1	2	3 (30.0)
Total	7	3	10 (100.0)
**Virus**			
Adenovirus	4	2	6 (5.5)
Coronavirus	6	10	16 (14.5)
Human Metapneumovirus	3	10	13 (11.8)
Human Rhinovirus/Enterovirus	7	12	19 (17.3)
Influenza A	9	13	22 (20.0)
Influenza B	0	1	1 (0.91)
Parainfluenza Virus	15	12	27 (24.5)
Respiratory Syncytial Virus	2	4	6 (5.5)
Total	46	64	110 (100.0)

*297 BAL specimens and 261 sputum specimens were included in this study. Multiple pathogens could be detected in a single specimen.

Among the positive specimens, 595 bacterial pathogens, 10 atypical bacterial pathogens, and 110 viral pathogens were identified. The most frequently detected bacterial pathogen was *Klebsiella pneumoniae* (18.7%), followed by *Pseudomonas aeruginosa* (15.3%), *Staphylococcus aureus* (14.3%), and *Haemophilus influenzae* (12.3%). *L. pneumophila* was the most frequently detected atypical bacterial pathogen (60.0%). Among viral pathogens, parainfluenza virus (24.5%), influenza A virus (20.0%), and human rhinovirus/enterovirus (17.3%) were the most frequently detected. Viral pathogens were identified more often in sputum specimens than in BAL specimens.

### Comparison of culture and FilmArray results

Among the 297 BAL specimens, 35.0% were positive by both conventional culture and the FilmArray Pneumonia Panel, whereas 41.1% were negative by both methods. The remaining specimens showed discordant results: 20.2% were culture-negative but FilmArray-positive, whereas only 3.7% were culture-positive but FilmArray-negative.

Similarly, among the 261 sputum specimens, 31.0% were positive by both methods and 40.2% were negative by both conventional culture and the FilmArray Pneumonia Panel. In contrast, 28.0% of sputum specimens were culture-negative but FilmArray-positive, whereas only 0.8% were culture-positive but FilmArray-negative.

At the organism-level, the FilmArray Pneumonia Panel detected substantially more bacterial pathogens than conventional culture in culture-negative BAL and sputum specimens, whereas only a limited number of bacterial pathogens were detected exclusively by conventional culture (Tables 2 and 3).

**Table 2:** Bacterial pathogens detected exclusively by FilmArray Pneumonia Panel or by conventional culture in bronchoalveolar lavage specimens.

Bacterial pathogens	FilmArray positive, Culture negative, n	Culture positive, FilmArray negative, n
*Acinetobacter calcoaceticus–baumannii* complex	14	2
*Enterobacter cloacae* complex	4	0
*Escherichia coli*	17	1
*Haemophilus influenzae*	24	0
*Klebsiella aerogenes*	12	0
*Klebsiella oxytoca*	3	0
*Klebsiella pneumoniae* group	22	8
*Proteus spp.*	16	0
*Pseudomonas aeruginosa*	30	0
*Serratia marcescens*	5	0
*Staphylococcus aureus*	26	2
*Streptococcus agalactiae*	11	0
*Streptococcus pneumoniae*	14	0
Total	198	13

**Table 3:** Bacterial pathogens detected exclusively by FilmArray Pneumonia Panel or by conventional culture in sputum specimens.

Bacterial pathogens	FilmArray positive, Culture negative, n	Culture positive, FilmArray negative, n
*Acinetobacter calcoaceticus–baumannii* complex	14	3
*Enterobacter cloacae* complex	6	0
*Escherichia coli*	20	0
*Haemophilus influenzae*	39	0
*Klebsiella aerogenes*	3	0
*Klebsiella oxytoca*	4	0
*Klebsiella pneumoniae* group	23	3
*Moraxella catarrhalis*	4	0
*Proteus* spp.	12	0
*Pseudomonas aeruginosa*	21	1
*Serratia marcescens*	2	0
*Staphylococcus aureus*	36	1
*Streptococcus agalactiae*	13	0
*Streptococcus pneumoniae*	17	0
Total	214	8

Footnote

Values represent the number of organism detections, not the number of specimens. Because multiple bacterial pathogens could be detected in a single specimen, the summed frequencies exceed the total number of specimens.

### Antimicrobial resistance gene detection

A total of 212 AMR genes were detected, comprising 118 in BAL specimens and 94 in sputum specimens (Table 4). The most frequently detected AMR gene was CTX-M, accounting for 47.2% of all detected AMR genes, followed by *mecA/C* with MREJ (25.0%) and KPC (16.5%). The detected carbapenemase genes included NDM (6.1%), VIM (2.8%), IMP (1.9%), and OXA-48-like (0.5%). Overall, carbapenemase genes accounted for 27.8% of all detected AMR genes.

**Table 4:** Antimicrobial resistance determinants detected by the FilmArray Pneumonia Panel.

Antimicrobial resistance gene and/or protein	BAL N	Sputum N	Total N (%)
CTX-M	54	46	100 (47.2)
IMP	1	3	4 (1.9)
KPC	21	14	35 (16.5)
*mecA/C* and MREJ	29	24	53 (25.0)
NDM	9	4	13 (6.1)
OXA-48-like	1	0	1 (0.5)
VIM	3	3	6 (2.8)
Total	118	94	212 (100.0)

*297 BAL *specimens and 261 sputum specimens were included in this study. Multiple antimicrobial resistance genes could be detected in a single specimen. Abbreviations:* BAL*, bronchoalveolar lavage.*

### Polymicrobial detection patterns

Among the 359 FilmArray-positive specimens, 177 (49.3%) contained a single pathogen. Two pathogens were detected in 96 specimens (26.7%), three pathogens in 44 specimens (12.3%), and four or more pathogens in 42 specimens (11.7%). Overall, multiple pathogens were detected in 182 specimens (50.7%), with a maximum of eight pathogens identified in a single specimen. Detection of multiple pathogens appeared to be more common in sputum specimens (53.7%) than in BAL specimens (47.8%).

### Diagnostic performance compared with conventional culture

For BAL specimens, the FilmArray Pneumonia Panel demonstrated an overall sensitivity of 90.4% and specificity of 67.0% across all bacterial targets (Supplementary Table 1). The overall positive percent agreement (PPA) and negative percent agreement (NPA) were 74.6% and 77.5%, respectively. Among the major bacterial pathogens, sensitivity was 100% for *P. aeruginosa*, *H. influenzae*, and *S. pneumoniae*.

Similarly, in sputum specimens, the FilmArray Pneumonia Panel demonstrated an overall sensitivity of 97.6% and specificity of 59.0% (Supplementary Table 2). The overall PPA and NPA were 68.4% and 73.7%, respectively. Sensitivity was highest for *Escherichia coli* (100%), *H. influenzae* (100%), *S. pneumoniae* (100%), *P. aeruginosa* (94.1%), and *K. pneumoniae* (90.6%).

### Baseline characteristics of the clinical-impact cohort

Baseline characteristics of the patients included in the clinical-impact analysis are summarized in Table 5. A total of 494 patients were included: 294 in the pre-implementation period and 200 in the FilmArray period. Baseline imbalances were observed for age, CRP, erythrocyte sedimentation rate, D-dimer level, and residence type, whereas the remaining baseline characteristics were generally comparable between the two groups. Accordingly, the primary multivariable analyses were adjusted for the three variables with complete data and standardized mean differences of ≥0.10 (age, CRP, and residence type), together with sex, ICU admission, and ECOG performance status, which were prespecified a priori because of their established prognostic importance in pneumonia. Erythrocyte sedimentation rate, procalcitonin, and D-dimer also had standardized mean differences of ≥0.10 but contained 6–8% missing data, predominantly in the pre-implementation period; therefore, these variables were evaluated in sensitivity analyses rather than included in the primary models. The same covariates were used for both propensity score weighting and multivariable adjustment to ensure consistency across the primary analyses.

**Table 5:** Baseline characteristics of patients included in the clinical-impact analysis.

Characteristic	Pre-implementation period (n = 294)	FilmArray period (n = 200)	P	SMD
Male sex, n (%)	200 (68.0)	140 (70.0)	0.715	0.04
Age, years	75.37 ± 13.57	71.03 ± 14.29	<0.001	**0.31**
Body mass index, kg/m²	21.87 ± 3.88	21.63 ± 4.16	0.537	0.06
ICU admission, n (%)	115 (39.2)	69 (34.7)	0.350	0.09
White blood cells, × 10³/µL	11.00 ± 6.01	11.25 ± 6.47	0.665	0.04
ESR, mm/h	48.73 ± 31.39	53.13 ± 32.31	0.147	**0.14**
CRP, mg/L	104.70 ± 88.08	133.80 ± 107.50	0.002	**0.30**
Procalcitonin, ng/mL	3.33 ± 10.96	4.49 ± 12.80	0.307	**0.10**
Lactate, mmol/L	2.18 ± 2.00	2.09 ± 2.06	0.748	0.04
Albumin, g/dL	3.28 ± 0.61	3.24 ± 0.61	0.462	0.07
D-dimer, µg/mL	3.63 ± 4.35	2.79 ± 3.45	0.021	**0.21**
Temperature, °C	36.36 ± 3.05	36.25 ± 3.68	0.728	0.03
PaO_2_, mmHg	69.73 ± 50.23	65.85 ± 45.17	0.521	0.08
ECOG performance status, n (%)			0.800	
0	124 (42.5)	91 (45.5)		0.06
1	73 (25.0)	47 (23.5)		0.04
≥2	95 (32.5)	62 (31.0)		0.03
Residence before admission, n (%)			0.011	
Home	164 (56.4)	83 (41.5)		**0.30**
Nursing home	1 (0.3)	1 (0.5)		0.02
Long-term care facility	42 (14.4)	44 (22.0)		**0.20**
Acute care hospital	84 (28.9)	72 (36.0)		**0.15**

Continuous variables are presented as mean ± standard deviation and categorical variables as n (%).

P values were calculated using Welch’s t test for continuous variables and the chi-square test for categorical variables.

An absolute standardized mean difference (SMD) ≥0.10 (shown in bold) was considered to indicate meaningful imbalance. Variables with SMD ≥ 0.10 (age, C-reactive protein, and residence type) and variables selected a priori because of their established prognostic importance in pneumonia (sex, ICU admission, and ECOG performance status) were included in the multivariable models.

Residence: Because only two patients resided in nursing homes, this category was combined with long-term care facilities in the multivariable and propensity-score analyses, resulting in three categories (home, long-term care, and acute-care hospital). The original four-category distribution is shown here for completeness. The P value for the four-category comparison was calculated using Fisher’s exact test.

Abbreviations: ECOG, Eastern Cooperative Oncology Group; ESR, erythrocyte sedimentation rate; CRP, C-reactive protein; ICU, intensive care unit; SMD, standardized mean difference.

### Clinical impact of FilmArray testing

The primary clinical-impact endpoint was result-driven antibiotic action (initiation or modification). After adjustment for prespecified covariates, patients in the FilmArray period were significantly more likely to undergo antibiotic initiation or modification within five days after microbiological reporting than those in the pre-implementation period (40.5% vs. 26.5%; adjusted odds ratio, 1.86; 95% confidence interval [CI], 1.24–2.79; P = 0.003) (Table 6). The median turnaround time from specimen sampling to reporting of the final microbiological result was 3 days (interquartile range [IQR], 2–4) in the pre-implementation period and 0 days (IQR, 0–0) in the FilmArray period (mean difference, −3.17 days; 95% CI, −3.31 to −3.03; P < 0.001). Accordingly, the interval from sampling to the result-driven antibiotic action was more than three days shorter in the FilmArray period (3.91 ± 1.73 vs. 0.47 ± 1.23 days; adjusted mean difference, −3.53 days; 95% CI, −4.08 to −2.99; P < 0.001), whereas the interval from result reporting to the antibiotic action was shorter by approximately half a day (adjusted mean difference, −0.59 days; 95% CI, −1.07 to −0.10; P = 0.019), indicating that most of the gain derived from the faster diagnostic turnaround rather than from a faster clinician response once the result was available. The FilmArray period had a shorter hospital length of stay (adjusted mean difference, −1.61 days; 95% CI, −3.07 to −0.15; P = 0.031). However, the adjusted duration of antibiotic therapy during hospitalization was longer (adjusted mean difference, 1.86 days; 95% CI, 0.29–3.44; P = 0.021). No significant difference in 30-day mortality was observed between the two groups.

**Table 6:** Clinical impact of the FilmArray Pneumonia Panel on antibiotic management and clinical outcomes.

Outcome	Analysis population	Pre-implementation period (n = 294)	FilmArray period (n = 200)	Effect estimate (95% CI) ‡	P ‡
Result-driven antibiotic action within 5 days, n (%) §	All patients	78 (26.5%)	81 (40.5%)	OR 1.86 (1.24–2.79)	0.003
Turnaround time from sampling to result reporting, days ††	All patients (n = 293 / 199)*	3.18 ± 1.21 3 (2–4)	0.02 ± 0.12 0 (0–0)	−3.17 (−3.31 to −3.03)	<0.001
Time from sampling to antibiotic action, days ††	Patients with result-driven action (n = 78 / 81)*	3.91 ± 1.73 4 (2–5)	0.47 ± 1.23 0 (0–0)	−3.53 (−4.08 to −2.99)	<0.001
Time from result reporting to antibiotic action, days	Patients with result-driven action (n = 78 / 81)*	1.03 ± 1.35 0 (0–2)	0.47 ± 1.23 0 (0–0)	−0.59 (−1.07 to −0.10)	0.019
Total antibiotic days ¶	All patients receiving antibiotics (n = 277 / 183)*	11.96 ± 8.44 10 (7–16)	13.55 ± 7.99 12 (8–17)	+1.86 (+0.29 to +3.44)	0.021
Length of hospital stay, days **	All patients (n = 200 / 157)*	15.30 ± 6.64 14 (10–20)	13.46 ± 7.07 11 (8–18)	−1.61 (−3.07 to −0.15)	0.031
30-day mortality, n (%)***	All patients	58/293 (19.8%)	46/199 (23.1%)	OR 1.50 (0.90–2.48)	0.118

Continuous variables are presented as mean ± standard deviation (upper line) and median (interquartile range) (lower line).

**‡** Adjusted for age, sex, ICU admission, CRP, ECOG performance status, and residence type (collapsed into three categories: home, long-term care facility, and acute-care hospital). Continuous outcomes were analyzed using multivariable linear regression with heteroscedasticity-consistent (HC3) standard errors; adjusted effects are presented as mean differences (FilmArray period minus pre-implementation period). Binary outcomes were analyzed using multivariable logistic regression; adjusted effects are presented as odds ratios. Results were confirmed in a propensity score–weighted sensitivity analysis using stabilized inverse probability of treatment weights.

**§** Result-driven antibiotic action was defined as initiation of a new antibiotic or modification of an existing regimen on the day of, or within 5 days after, reporting of the final microbiological result. Antibiotic changes preceding microbiological reporting were not considered result-driven.

†† Sampling refers to the date on which the respiratory specimen was obtained. The turnaround time (sampling to reporting of the final result) is presented without adjustment, because it is a property of the diagnostic method rather than of the patient.

**¶** Antibiotic courses exceeding 45 days were excluded (14 in the pre-implementation period and 15 in the FilmArray period). Antibiotic duration could not be calculated for an additional 3 and 2 patients, respectively, because of incomplete medication records. Sensitivity analyses including these patients yielded consistent results (adjusted ratio of means, 1.37; 95% CI, 1.16–1.62; P < 0.001; log-transformed regression model).

* Number of patients analyzed during pre-implementation period/FilmArray period.

** Hospital stays exceeding 30 days were excluded (93 patients [31.6%] in the pre-implementation period and 41 [20.5%] in the FilmArray period; P = 0.009 for the difference in exclusion rates). Because the exclusion rates differed between the two study periods, the analysis was repeated in the full cohort without the 30-day restriction. In this sensitivity analysis, an even larger difference was observed (mean, 31.55 vs. 21.74 days; adjusted mean difference, −9.22 days; 95% CI, −14.16 to −4.28; P < 0.001; adjusted ratio of means in a log-transformed model, 0.74; 95% CI, 0.64–0.85). These findings suggest that the 30-day restriction may have resulted in a conservative estimate of the effect. Patients who died during hospitalization were retained, with length of stay measured to the date of death. Results were consistent when the analysis was restricted to survivors (adjusted mean difference, −1.62 days; 95% CI, −3.15 to −0.10; P = 0.037).

*** Vital status at 30 days could not be ascertained in one patient in each period, both of whom were transferred to another institution before day 30; these two patients were excluded from the 30-day mortality analysis, and assigning them to either outcome did not alter the results.

**Abbreviations:** CI, confidence interval; ECOG, Eastern Cooperative Oncology Group; ICU, intensive care unit; OR, odds ratio.

### Patterns of antibiotic initiation and modification

When the whole clinical-impact cohort was taken as the denominator, result-guided initiation of antibiotics was markedly more frequent in the FilmArray period than in the pre-implementation period (27.5% vs. 3.4%; adjusted odds ratio, 9.20; 95% CI, 4.48–18.90; P < 0.001), and the proportion of patients in whom no antibiotic action followed the microbiological result fell from 73.5% to 59.5% (adjusted odds ratio, 0.54; 95% CI, 0.36–0.81; P = 0.003) (Table 7). Escalation (15.0% vs. 9.0%; P = 0.053) and de-escalation (8.2% vs. 4.0%; P = 0.092) of an ongoing regimen were numerically less frequent in the FilmArray period, but neither difference was statistically significant before or after adjustment. The overall four-category distribution differed between the periods (chi-square P < 0.001). Implementation of the panel therefore changed the character of result-driven decisions − towards initiation of targeted therapy in patients not yet receiving antibiotics − rather than the frequency of escalation or de-escalation. Nevertheless, even in the FilmArray period, 59.5% of patients had no antibiotic action attributable to the microbiological result within five days.

**Table 7:** Antibiotic actions attributable to the microbiological result in the whole clinical-impact cohort.

Pattern of antibiotic action	Pre-implementation period (n = 294)	FilmArray period (n = 200)	P
Result-guided initiation of antibiotics †	10 (3.4)	55 (27.5)	<0.001
Escalation of an ongoing regimen	44 (15.0)	18 (9.0)	0.053
De-escalation of an ongoing regimen	24 (8.2)	8 (4.0)	0.092
No antibiotic action attributable to the result	216 (73.5)	119 (59.5)	0.001

Values are presented as n (%). Percentages are of all patients included in the clinical-impact analysis in each period (294 and 200, respectively), so that patients in whom the regimen was deliberately left unchanged after the microbiological result are represented; the four categories are mutually exclusive and sum to 100%. The four-category distribution differed between the periods (chi-square P < 0.001). Adjusted odds ratios (FilmArray vs. pre-implementation period), from multivariable logistic regression with the covariates listed in Table 6, were 9.20 (95% CI, 4.48–18.90; P < 0.001) for result-guided initiation, 0.55 (95% CI, 0.29–1.03; P = 0.061) for escalation, 0.58 (95% CI, 0.24–1.40; P = 0.226) for de-escalation, and 0.54 (95% CI, 0.36–0.81; P = 0.003) for no antibiotic action. P values in the table were calculated using Fisher’s exact test comparing each category with all other patients in the same period.

† Result-driven initiation was defined as initiation of antibiotic therapy on the day of, or within 5 days after, reporting of the microbiological result in patients who were not receiving antibiotics at the time of reporting. In the pre-implementation period, empirical antibiotic therapy had almost always been initiated before culture results became available; therefore, result-guided initiation was uncommon in this period (10 of 294 patients, 3.4%).

## Discussion

In this real-world before-and-after observational study, the BioFire FilmArray Pneumonia Panel demonstrated high diagnostic sensitivity and broad pathogen coverage in both BAL and sputum specimens and was associated with clinically meaningful improvements in microbiology-guided antibiotic management. Whereas previous studies have primarily focused on the analytical validation or diagnostic performance of the FilmArray Pneumonia Panel, our study evaluated both diagnostic performance and downstream clinical impact in hospitalized patients with pneumonia, thereby providing a more comprehensive assessment of its clinical utility in routine clinical practice.

Consistent with previous international studies, the FilmArray Pneumonia Panel frequently detected clinically important bacterial pathogens, including *K. pneumoniae*, *P. aeruginosa*, and *S. aureus* [4,6]. These organisms are commonly associated with multidrug resistance and poor clinical outcomes. In addition, respiratory viruses and atypical bacterial pathogens were frequently identified, particularly in sputum specimens, highlighting the broad etiologic spectrum of lower respiratory tract infections and the limitations of conventional culture in detecting these pathogens.

A prominent finding of our study was the high proportion of specimens that were culture-negative but FilmArray-positive, particularly among sputum specimens. This observation reflects the well-recognized limitations of conventional culture, including reduced sensitivity following prior antibiotic exposure and its inability to detect fastidious or slow-growing organisms [2,6]. Similar findings have been reported in critically ill patients and patients with COVID-19, in whom early empirical antibiotic therapy substantially decreases culture yield [6,7,15]. Furthermore, because conventional culture does not detect respiratory viruses or atypical bacterial pathogens, part of this discordance is expected and should not necessarily be interpreted as false-positive FilmArray results.

Despite these advantages, conventional culture remains an indispensable component of the microbiological evaluation of pneumonia. Unlike targeted multiplex PCR assays, culture can detect a broader range of bacterial and fungal pathogens, including unexpected organisms not covered by the FilmArray panel. In addition, semi-quantitative culture results may help distinguish colonization from true infection, while culture enables phenotypic antimicrobial susceptibility testing and further molecular characterization. Accordingly, the FilmArray Pneumonia Panel should be regarded as a complementary diagnostic tool rather than a replacement for conventional microbiological culture.

Although previous systematic reviews have reported higher diagnostic odds ratios and better clinical agreement for BAL specimens than for sputum specimens [16,17], our study was not designed to compare diagnostic performance between specimen types. Furthermore, previous studies have reported only modest agreement between FilmArray results and the final clinical diagnosis of pneumonia [18], emphasizing that molecular findings should always be interpreted within the overall clinical context.

The detection of antimicrobial resistance (AMR) genes further underscores the clinical value of FilmArray testing. In our cohort, CTX-M and mecA/C with MREJ were the most frequently detected AMR genes, reflecting the high prevalence of extended-spectrum β-lactamase-producing Enterobacterales and methicillin-resistant S. aureus among hospitalized patients with pneumonia. During the FilmArray study period (2023), local antimicrobial resistance surveillance demonstrated that approximately 20% of *E. coli* and 30% of *K. pneumoniae* isolates were ESBL producers, whereas carbapenem resistance was identified in approximately 1% of *E. coli* and 10% of *K. pneumoniae* isolates. In addition, approximately 50% of *S. aureus* isolates were methicillin resistant. These regional resistance patterns highlight the importance of rapid identification of both ESBL- and carbapenemase-associated resistance determinants, as well as methicillin-resistant *S. aureus*, to support early optimization of antimicrobial therapy. These findings are consistent with previous antimicrobial stewardship studies demonstrating the importance of rapid resistance gene identification for guiding appropriate antimicrobial therapy [11,15,18]. Early identification of resistance determinants may facilitate timely escalation when resistant pathogens are present while avoiding unnecessary broad-spectrum therapy when resistance genes are absent [2].

Detection of multiple pathogens was common in our study, with more than half of FilmArray-positive specimens containing two or more detected microorganisms. This finding highlights the complex microbiology of pneumonia and illustrates an important advantage of multiplex molecular diagnostics over conventional culture and single-target molecular assays [11,16]. Recognition of multiple-pathogen detection may influence antimicrobial selection and overall treatment strategies; however, the clinical significance of each detected organism requires careful interpretation together with the patient’s clinical presentation and other microbiological findings [4,17]. As reported previously, multiple-pathogen detection was more frequent in sputum specimens than in BAL specimens [4]. Because no independent clinical adjudication was performed, the clinical significance of multiple-pathogen detection could not be determined, and these findings should not be interpreted as definitive evidence of true polymicrobial infection.

Despite these diagnostic advantages, the moderate specificity observed for the FilmArray Pneumonia Panel warrants careful interpretation. This limitation is largely attributable to its high analytical sensitivity, which may detect colonizing organisms, residual nucleic acids from nonviable bacteria, or microorganisms below the threshold of clinical significance. Consequently, positive molecular results do not necessarily indicate active infection and, if interpreted without adequate clinical correlation, may lead to unnecessary initiation or escalation of antimicrobial therapy. Therefore, FilmArray results should always be interpreted together with the patient’s clinical presentation, radiologic findings, and conventional microbiological results.

The relatively high cost of multiplex molecular testing may also limit its widespread implementation in routine clinical practice. Future studies evaluating the cost-effectiveness and scalability of the FilmArray Pneumonia Panel in different healthcare settings are warranted.

Beyond diagnostic performance, implementation of the FilmArray Pneumonia Panel facilitated earlier and more frequent microbiology-guided antibiotic management. Measured from specimen sampling, patients in the FilmArray period underwent a result-driven antibiotic action more than three days earlier than those in the pre-implementation period, whereas the gain measured from result reporting was only about half a day. Almost the whole of this benefit therefore derived from the faster diagnostic turnaround of the panel (median, 0 vs. 3 days) rather than from a faster clinician response once the result was available, supporting the role of rapid molecular diagnostics in improving the timeliness of antimicrobial decision-making, one of the principal goals of antimicrobial stewardship programs.

Previous studies have reported reductions in overall antibiotic exposure following implementation of the FilmArray Pneumonia Panel [19]. However, our adjusted analyses did not demonstrate this benefit. Instead, total antibiotic exposure during hospitalization was modestly longer in the FilmArray period despite earlier microbiology-guided antibiotic modification. One possible explanation is that rapid identification of clinically important pathogens and AMR genes prompted continuation or escalation of appropriate therapy in patients with microbiologically confirmed or more severe infections rather than premature discontinuation of antibiotics. These findings suggest that rapid molecular diagnostics primarily improve the appropriateness and timeliness of antibiotic management rather than simply reducing antibiotic use.

When the whole cohort was taken as the denominator, escalation and de-escalation of an ongoing regimen were numerically less frequent in the FilmArray period, but neither difference was statistically significant; what changed was the character of the decision, with a nine-fold increase in the adjusted odds of result-guided initiation of targeted therapy and a fall in the proportion of patients in whom the result changed nothing (73.5% to 59.5%). Nevertheless, the microbiological result still did not alter management within five days in 59.5% of patients in the FilmArray period, and we did not observe an increase in antibiotic de-escalation. Clinicians appeared to continue relying primarily on conventional culture and phenotypic antimicrobial susceptibility testing when making definitive antimicrobial decisions, consistent with previous reports [12,20,21]. This may reflect uncertainty regarding the interpretation of multiple-pathogen detection, the clinical significance of molecular findings, or the limited predictive value of resistance gene detection alone [12]. These findings emphasize that successful implementation of rapid molecular diagnostics requires close integration with antimicrobial stewardship programs as well as clinician education to optimize interpretation and clinical decision-making [3,20,22].

FilmArray implementation was also associated with a modest reduction in hospital length of stay after adjustment for baseline characteristics, although no significant difference in 30-day mortality was observed. Similar findings have been reported in previous stewardship-focused investigations evaluating multiplex molecular diagnostics [19,23]. Although causal relationships cannot be established because of the observational study design, earlier pathogen identification and more timely microbiology-guided antibiotic management may contribute to improved clinical efficiency and healthcare resource utilization.

Several limitations should be acknowledged. First, this was a retrospective before-and-after observational study; therefore, temporal changes in clinical practice, antimicrobial stewardship activities, patient case mix, as well as other unmeasured factors may have contributed to the observed differences between the two study periods. Although multivariable adjustment and propensity score weighting were performed to reduce baseline imbalances, residual confounding cannot be excluded. Second, conventional culture was used as the reference standard despite its limited sensitivity, which may have affected estimates of the apparent diagnostic specificity of the FilmArray Pneumonia Panel. Third, antimicrobial management was not standardized and may have been influenced by individual clinician judgment and institutional antimicrobial stewardship practices. Fourth, we did not evaluate the semi-quantitative bacterial load categories or cycle threshold values reported by the FilmArray Pneumonia Panel, nor did we systematically compare detected antimicrobial resistance genes with the results of conventional antimicrobial susceptibility testing. These parameters should be evaluated in future studies. Fifth, hospital stays exceeding 30 days were excluded in different proportions in the two study periods (31.6% vs. 20.5%). However, analysis of the full cohort without this restriction showed a larger effect in the same direction, suggesting that the primary estimate may have been conservative. Sixth, no routine reference method for respiratory viruses was available during the study period; therefore, the diagnostic performance of the viral targets could not be evaluated, and we were unable to determine how positive viral results influenced antimicrobial prescribing decisions. Finally, although the FilmArray Pneumonia Panel provides rapid microbiological information, antibiotic prescribing decisions are influenced by multiple clinical factors in addition to microbiological test results. Clinical improvement or deterioration, radiologic findings, inflammatory markers, and other patient-specific considerations may all contribute to treatment modification. Therefore, although antibiotic actions were evaluated only when they occurred within five days of result reporting, this temporal association should not be interpreted as establishing a causal relationship between FilmArray implementation and changes in antibiotic prescribing. Future prospective studies should evaluate the proportion of clinically actionable FilmArray results and their direct effects on antimicrobial stewardship and patient outcomes.

## Conclusion

In conclusion, the BioFire FilmArray Pneumonia Panel demonstrated high diagnostic sensitivity and broad pathogen coverage in both BAL and sputum specimens. Compared with conventional culture, the panel detected additional microorganisms and antimicrobial resistance genes; however, this study was not designed to determine whether these additional detections represented the true causative pathogens or clinically relevant resistance mechanisms. Therefore, FilmArray results should always be interpreted in conjunction with conventional microbiological testing and the overall clinical context.

In the clinical-impact analysis, implementation of the FilmArray Pneumonia Panel facilitated earlier and more frequent microbiology-guided antibiotic management and was associated with a modest reduction in hospital length of stay. However, implementation was associated with modestly increased overall antibiotic exposure and did not significantly increase antibiotic de-escalation. These findings support the role of rapid multiplex molecular diagnostics as an adjunct to conventional microbiological testing for improving the timeliness and precision of antimicrobial therapy. Nevertheless, their full clinical benefit is likely to be realized only when integrated with structured antimicrobial stewardship programs.

## Supporting information

S1 Table Diagnostic performance of the FilmArray Pneumonia Panel compared with conventional culture in bronchoalveolar lavage specimens. (PDF)

S2 Table Diagnostic performance of the FilmArray Pneumonia Panel compared with conventional culture in sputum specimens. (PDF)

S3 Table STROBE Checklist excluding participant flow figure. (DOCX)

S4 Table STARD 2015 Checklist. (DOCX)

S1 Fig Flow diagram of participants included in the clinical-impact analysis. (TIF)
