# Supplementary material for: Diagnostic performance and clinical impact of the BioFire FilmArray Pneumonia Panel compared with conventional culture in hospitalized patients with pneumonia
Source: PLoS One. 2026 Sep 28;21(9):e0358986. doi: 10.1371/journal.pone.0358986 (PMC13618948; doi:10.1371/journal.pone.0358986)
Supplement: Supplementary file 1 [file pone.0358986.s001.pdf]

Supplementary Table 1. Performance of the FilmArray Pneumonia Panel compared with conventional culture on BAL specimens.

Target	No. of samples									
	Culture - Film Array-	Culture - Film Array+	Culture + Film Array-	Culture + Film Array+	Sensitivity (95% CI)	Specificity (95% CI)	PPV (95% CI)	NPV (95% CI)	PPA (95% CI)	NPA (95% CI)
Acinetobacter baumannii	265	12	2	18	90.0 (68.3-98.8)	95.7 (92.6-97.7)	60.0 (40.6-77.3)	99.3 (97.3-99.9)	72.0 (57.5-83.8)	97.4 (95.7-98.6)
Enterobacter cloacae complex	293	4	0	0	NA	98.7 (96.6-99.6)	0.0 (0.0-60.2)	100.0 (98.7-100.0)	0.0 (0.0-60.2)	99.3 (98.3-99.8)
Escherichia coli	276	17	1	3	75.0 (19.4-99.4)	94.2 (90.9-96.6)	15.0 (3.2-37.9)	99.6 (98.0-100.0)	25.0 (9.8-46.7)	96.8 (95.5-98.1)
Haemophilus influenzae	268	24	0	5	100.0 (47.8-100.0)	91.8 (88.0-94.7)	17.2 (5.8-35.8)	100.0 (98.6-100.0)	29.4 (15.1-47.5)	95.7 (93.7-97.2)
Klebsiella aerogenes	285	12	0	0	NA	96.0 (93.0-97.9)	0.0 (0.0-26.5)	100.0 (98.7-100.0)	0.0 (0.0-26.5)	97.9 (96.4-98.9)

Klebsiella oxytoca	294	3	0	0	NA	99.0 (97.1-99.8)	0.0 (0.0-70.8)	100.0 (98.8-100.0)	0.0 (0.0-70.8)	99.5 (98.5-99.9)
Klebsiella pneumoniae	230	22	8	37	82.2 (67.9-92.0)	91.3 (87.1-94.4)	62.7 (49.1-75.0)	96.6 (93.5-98.5)	71.2 (61.4-79.6)	93.9 (91.4-95.8)
Moraxella catarrhalis	297	0	0	0	NA	NA	NA	NA	NA	NA
Proteus spp.	281	16	0	0	NA	94.9 (91.4-96.9)	0.0 (0.0-20.6)	100.0 (98.7-100.0)	0.0 (0.0-20.6)	97.2 (95.5-98.4)
Pseudomonas aeruginosa	243	30	0	24	100.0 (85.8-100.0)	89.0 (84.6-92.4)	44.4 (30.9-58.6)	100.0 (98.5-100.0)	61.5 (49.8-72.3)	94.2 (91.8-96.0)
Serratia marcescens	290	5	0	2	100.0 (15.8-100.0)	98.3 (96.1-99.4)	28.6 (3.7-71.0)	100.0 (98.7-100.0)	44.4 (13.7-78.8)	99.1 (98.0-99.7)
Staphylococcus aureus	253	26	2	16	88.9 (65.3-98.6)	90.7 (86.6-93.8)	38.1 (23.6-54.4)	99.2 (97.2-99.9)	53.3 (40.0-66.3)	94.8 (92.5-96.5)
Streptococcus agalactiae	286	11	0	0	NA	96.3 (93.5-98.1)	0.0 (0.0-28.5)	100.0 (98.7-100.0)	0.0 (0.0-28.5)	98.1 (96.6-99.1)
Streptococcus pneumoniae	281	14	0	2	100.0 (15.8-100.0)	95.3 (92.2-97.4)	12.5 (1.6-38.3)	100.0 (98.7-100.0)	22.2 (6.4-47.6)	97.6 (96.0-98.7)

Total	122	60	11	104	90.4 (83.5-95.1)	67.0 (59.7-73.8)	63.4 (55.5-70.8)	91.7 (85.7-95.8)	74.6 (69.0-79.6)	77.5 (72.4-82.0)

Abbreviations: BAL, broncho-alveolar lavage; CI, confidence interval; PPV, positive predictive value; NPV, negative predictive value; PPA, positive percent agreement; NPA, negative percent agreement.
