# Supplementary material for: Diagnostic performance and clinical impact of the BioFire FilmArray Pneumonia Panel compared with conventional culture in hospitalized patients with pneumonia
Source: PLoS One. 2026 Sep 28;21(9):e0358986. doi: 10.1371/journal.pone.0358986 (PMC13618948; doi:10.1371/journal.pone.0358986)
Supplement: Supplementary file 2 [file pone.0358986.s002.pdf]

Supplementary Table 2. Performance of the FilmArray Pneumonia Panel compared with conventional culture on sputum specimens.

Target	No. of samples				Sensitivity (95% CI)	Specificity (95% CI)	PPV (95% CI)	NPV (95% CI)	PPA (95% CI)	NPA (95% CI)
	Culture - Film Array-	Culture - Film Array+	Culture + Film Array-	Culture + Film Array+						
Acinetobacter baumannii:	236	14	3	8	72.7 (39.0-94.0)	94.4 (90.8-96.9)	36.4 (17.2-59.3)	98.7 (96.4-99.7)	48.5 (30.8-66.5)	96.5 (94.5-98.0)
Enterobacter cloacae complex	255	6	0	0	NA	97.7 (95.1-99.2)	0.0 (0.0-45.9)	100.0 (98.6-100.0)	0.0 (0.0-45.9)	98.8 (97.5-99.6)
Escherichia coli	233	20	0	8	100.0 (63.1-100.0)	92.1 (88.1-95.1)	28.6 (13.2-48.7)	100.0 (98.4-100.0)	44.4 (27.9-61.9)	95.9 (93.7-97.5)
Haemophilus influenzae	217	39	0	5	100.0 (47.8-100.0)	84.8 (79.8-88.9)	11.4 (3.8-24.6)	100.0 (98.3-100.0)	20.4 (10.2-34.3)	91.8 (88.9-94.1)
Klebsiella aerogenes	258	3	0	0	NA	98.9 (96.7-99.8)	0.0 (0.0-70.8)	100.0 (98.6-100.0)	0.0 (0.0-70.8)	99.4 (98.3-99.9)

Klebsiella oxytoca	256	4	0	1	100.0 (2.5-100.0)	98.5 (96.1-99.6)	20.0 (0.5-71.6)	100.0 (98.6-100.0)	33.3 (4.3-77.7)	99.2 (98.0-99.8)
Klebsiella pneumoniae	206	23	3	29	90.6 (75.0-98.0)	90.0 (85.3-93.5)	55.8 (41.3-69.5)	98.6 (95.9-99.7)	69.0 (58.0-78.7)	94.1 (91.4-96.1)
Moraxella catarrhalis	257	4	0	0	NA	98.5 (96.1-99.6)	0.0 (0.0-60.2)	100.0 (98.6-100.0)	0.0 (0.0-60.2)	99.2 (98.0-99.8)
Proteus spp.	249	12	0	0	NA	95.4 (92.1-97.6)	0.0 (0.0-26.5)	100.0 (98.5-100.0)	0.0 (0.0-26.5)	97.6 (95.9-98.8)
Pseudomonas aeruginosa	223	21	1	16	94.1 (71.3-99.9)	91.4 (87.1-94.6)	43.2 (27.1-60.5)	99.6 (97.5-100.0)	59.3 (45.0-72.4)	95.3 (93.0-97.0)
Serratia marcescens	257	2	0	2	100.0 (15.8-100.0)	99.2 (97.2-99.9)	50.0 (6.8-93.2)	100.0 (98.6-100.0)	66.7 (22.3-95.7)	99.6 (98.6-100.0)
Staphylococcus aureus	217	36	1	7	87.5 (47.3-99.7)	85.8 (80.8-89.8)	16.3 (6.8-30.7)	99.5 (97.5-100.0)	27.5 (15.9-41.7)	92.1 (89.33-94.4)
Streptococcus agalactiae	248	13	0	0	NA	95.0 (91.6-97.3)	0.0 (0.0-24.7)	100.0 (98.5-100.0)	0.0 (0.0-24.7)	97.4 (95.7-98.6)

Streptococcus pneumoniae	242	17	0	2	100.0 (15.8-100.0)	93.4 (89.7-96.1)	10.5 (1.3-33.1)	100.0 (98.5-100.0)	19.0 (5.4-41.9)	96.6 (94.6-98.0)
Total	105	73	2	81	97.6 (91.6-99.7)	59.0 (51.4-66.3)	52.6 (44.4-60.7)	98.1 (93.4-99.8)	68.4 (62.0-74.2)	73.7 (68.2-78.7)

Abbreviations: BAL, broncho-alveolar lavage; CI, confidence interval; PPV, positive predictive value; NPV, negative predictive value; PPA, positive percent agreement; NPA, negative percent agreement.
