# Supplementary material for: Diagnostic performance and clinical impact of the BioFire FilmArray Pneumonia Panel compared with conventional culture in hospitalized patients with pneumonia
Source: PLoS One. 2026 Sep 28;21(9):e0358986. doi: 10.1371/journal.pone.0358986 (PMC13618948; doi:10.1371/journal.pone.0358986)
Supplement: Supplementary file 3 [file pone.0358986.s007.pdf]

I would like to thank the Editor, the other Reviewers and the Authors for thorough work on this article. Also, I would like to congratulate the authors on the comprehensive corrections in the revised version and the improvements made in the article. However, as the changes made are quite extensive, there are some novel points I would like to raise for consideration to further improve the current version of the article. The two comments listed first here, I believe to be most pertinent, and comments 3-6 are mostly minor in nature.

1) One of the previous comments, concerning the time to result driven action, prompted extensive revision. I would like to add, that an additional important comparison might in fact be made comparing time from sampling to result driven decision: the main advantage of molecular testing generally being faster turn-around time. Thus, an important additional correction would be to report also time from sampling to result driven decision for both groups (pre-intervention period and FilmArray period) and use terminology that describes this clearly. For clarity, I believe the current comparison of time from microbiologic result to action should also remain in the article.

2) Table 6:

   The authors have explored the proportions of different types of actions in a subgroup of patients where some active action after microbiologic result was undertaken. I believe it would be more meaningful and informative to report the proportion of patients with specific modifications of all patients included in the clinical impact evaluation (that is, include in the denominator also the patients, where no modification was made). Deciding not to change the antibiotic regimen after the microbiological result is still a clinical decision even if it appears "passive". At the very minimum, a row indicating how many patients in each period did not have changes in the antibiotic regimen, should be added. But to demonstrate the possible difference seen with a different approach to the dataset, please find below as an example the proportions calculated from the total group included in the clinical comparison:

   pre-FilmArray period

   initiation: 10/294, 3.4%

   escalation: 44/294, 14.9%

   de-escalation: 24/294, 8.1%

   FilmArray period

initiation: 55/200, 27.5%

escalation: 18/200, 9%

de escalation: 8/200, 4%

Crudely, both de-escalation and escalation appear to be more frequent in the pre-intervention period, (possible statistical significance?). Please, consider this carefully and if you decide to maintain the initial approach, please provide justification in the article. I acknowledge that exploring the suggested approach might result in the need to revise the conclusions drawn and the discussion section, but I believe it to be an important enough question to warrant careful revision.

Detailed minor revisions:

3) Abstract, lines 47-48: I recommend removing the indirect ("double-negative") expression: "did not decrease" and substituting with more direct language: "increased antibiotic exposure".
4) Abstract lines 49-50: I suggest adding something to further clarify the role of molecular testing, for example: "when used as **a complementary tool to traditional microbiological methods and** integrated with structured ..."
5) Table 5: In the 30-day mortality comparison both groups are missing one patient. Based on Figure S1 this is missing data. Can the authors confirm whether this is correct? Missing data on 30-mortality is of course possible, but in small datasets (about 200 patients) usually rather rare (i.e. usually it is apparent whether patients are alive or not at the 30-day time point).
6) Figure S1: Pre-implementation box only mentions sputum culture, where I believe, should be also culture of BAL-samples.
