# Supplementary material for: Diagnostic performance and clinical impact of the BioFire FilmArray Pneumonia Panel compared with conventional culture in hospitalized patients with pneumonia
Source: PLoS One. 2026 Sep 28;21(9):e0358986. doi: 10.1371/journal.pone.0358986 (PMC13618948; doi:10.1371/journal.pone.0358986)
Supplement: Supplementary file 4 [file pone.0358986.s003.docx]

# Supplementary Table 3. STROBE Checklist excluding participant flow figure.

| Item | STROBE recommendation | Status | Location in manuscript |
| --- | --- | --- | --- |
| Title/Abstract | Indicate study design in title/abstract | Completed | Title; Abstract |
| Background | Explain scientific background and rationale | Completed | Introduction |
| Objectives | State specific objectives | Completed | Introduction |
| Study design | Present key elements early | Completed | Methods |
| Setting | Describe setting, locations, dates | Completed | Methods |
| Participants | Eligibility criteria and selection | Completed | Methods |
| Variables | Define outcomes and predictors | Completed | Methods |
| Data sources | Describe measurements | Completed | Methods |
| Bias | Describe efforts to address bias | Completed | Statistical analysis |
| Study size | Explain sample size | Completed | Methods |
| Statistical methods | Describe all analyses | Completed | Methods |
| Participants (Results) | Numbers at each stage | See Supplementary Figure S1 | Results |
| Descriptive data | Participant characteristics | Completed | Table 4 |
| Outcome data | Report outcome events | Completed | Results; Tables 5–6 |
| Main results | Provide estimates and CIs | Completed | Results |
| Limitations | Discuss limitations | Completed | Discussion |
| Interpretation | Overall interpretation | Completed | Discussion |
| Generalisability | Discuss external validity | Completed | Discussion |
| Funding | Funding statement | Completed | Declarations |

## Note

The participant flow diagram corresponding to STROBE/STARD participant reporting is provided separately as Supplementary Figure S1 and is therefore not included in this document.
