# Supplementary material for: Diagnostic performance and clinical impact of the BioFire FilmArray Pneumonia Panel compared with conventional culture in hospitalized patients with pneumonia
Source: PLoS One. 2026 Sep 28;21(9):e0358986. doi: 10.1371/journal.pone.0358986 (PMC13618948; doi:10.1371/journal.pone.0358986)
Supplement: Supplementary file 5 [file pone.0358986.s004.docx]

# Supplementary Table 4. STARD 2015 Checklist.

| Item | STARD 2015 Requirement | Location in Manuscript | Status |
| --- | --- | --- | --- |
| 1–2 | Title/Abstract identify diagnostic accuracy study; structured abstract | Title; Abstract | ✓ |
| 3–4 | Scientific background and objectives | Introduction | ✓ |
| 5 | Study design | Methods | ✓ |
| 6–9 | Participants, eligibility, setting, dates | Methods | ✓ |
| 10a | Index test | Methods | ✓ |
| 10b–11 | Reference standard and rationale | Methods | ✓ |
| 12a–13b | Test methods and positivity definitions | Methods | ✓ |
| 14 | Blinding | Methods (Statistical Analysis) | ✓ |
| 15–17 | Indeterminate results, missing data | Methods | ✓/NA |
| 18 | Sample size | Methods | ✓ |
| 19 | Flow diagram | Supporting Information | ✓ |
| 20–22 | Participant characteristics | Results | ✓ |
| 23 | Cross-tabulation | Tables 2A, 2B | ✓ |
| 24 | Accuracy estimates with 95% CI | Supplementary Tables 1–2 | ✓ |
| 25 | Adverse events | Not applicable | NA |
| 26–27 | Limitations and implications | Discussion | ✓ |
| 28–29 | Registration/protocol | Not applicable | NA |
| 30 | Funding | Funding | ✓ |
