# Supplementary material for: Diagnostic performance and clinical impact of the BioFire FilmArray Pneumonia Panel compared with conventional culture in hospitalized patients with pneumonia
Source: PLoS One. 2026 Sep 28;21(9):e0358986. doi: 10.1371/journal.pone.0358986 (PMC13618948; doi:10.1371/journal.pone.0358986)
Supplement: Supplementary file 6 [file pone.0358986.s008.docx]

**Response to Reviewers**

*Manuscript: Clinical Performance and Clinical Impact of the BioFire FilmArray Pneumonia Panel Compared with Conventional Culture in Hospitalized Patients with Pneumonia*

*PLOS ONE — Major Revision*

# General Response

We thank the Academic Editor and the three reviewers for their careful evaluation of our manuscript and for their constructive comments. Their feedback prompted us to conduct a comprehensive re-audit of the analytic dataset underlying the clinical-impact comparison. This re-examination identified several inconsistencies in the derivation of clinical outcome variables between the two study periods. We harmonized the operational definitions, recalculated all clinical-impact outcomes using identical criteria in both groups, and repeated the analyses using multivariable adjustment together with propensity score–based sensitivity analyses. These revisions have strengthened the methodological rigor and transparency of our study.

**Two reviewer comments prompted particularly extensive revisions.**

- **Reviewer #2** noted that our clinical-impact analysis relied entirely on unadjusted univariate comparisons despite meaningful baseline imbalance and asked whether the observed findings remained after appropriate statistical adjustment.
- **Reviewer #3** asked us to clarify whether time to antibiotic change had been measured from reporting of the microbiological result or from specimen collection, and pointed out that the reported value for the sputum culture group appeared implausibly long if it had indeed been measured from result reporting.

Reviewer #3 correctly identified an inconsistency in our original analysis. Re-examination showed that this outcome had been calculated from **specimen collection** in the sputum culture group but from **result reporting** in the FilmArray group. Because conventional sputum culture at our institution has a median turnaround time of approximately three days, whereas the FilmArray panel reports on the same day (median, 0 days), this inconsistency substantially exaggerated the apparent advantage of the panel. The audit also identified an inconsistent derivation of total antibiotic days between the two groups, together with a single implausible body mass index value (658.8 kg/m²) that distorted the mean and standard error reported in the baseline table.

Using the same 494-patient cohort (sputum culture group, n = 294; FilmArray group, n = 200), we therefore re-analyzed every clinical-impact outcome by applying a single pre-specified operational definition identically to both groups and incorporating the multivariable adjustment and propensity score–based sensitivity analyses requested by Reviewer #2. Accordingly, the baseline table, Tables 4 and 5, the Results, the Abstract, and the Conclusions have all been revised.

**The revised analyses resulted in an important revision of one of our original conclusions.**

Total antibiotic exposure was not reduced in the FilmArray group; instead, it was modestly but significantly increased (adjusted difference, +1.86 days; 95% CI, +0.29 to +3.44; P = 0.021). At the same time, implementation of the panel:

- increased the proportion of patients in whom the microbiological result led to an antibiotic decision, from 26.5% to 40.5% (adjusted OR, 1.86; 95% CI, 1.24–2.79; P = 0.003);
- shortened the interval between result reporting and that decision (adjusted difference, −0.59 days; 95% CI, −1.07 to −0.10; P = 0.019); and
- changed the nature of those decisions—from post hoc modification of already initiated empirical therapy (87.2% of actions in the culture group) to result-guided initiation of therapy in antibiotic-naïve patients (67.9% of actions in the FilmArray group; P < 0.001).

De-escalation, however, was no more frequent (30.8% vs. 35.3%; P = 0.81).

**Importantly,** these revised analyses resolve the apparent paradox identified by Reviewer #2. The inconsistency arose from differences in outcome derivation rather than from conflicting clinical findings. Once total antibiotic days were calculated identically in both groups, antibiotic exposure was consistently higher in the FilmArray group during both days 1–7 and the overall hospitalization. Both observations are explained by the same underlying pattern: the panel was used predominantly to initiate or broaden antibiotic therapy rather than to facilitate de-escalation. These findings are consistent with previously published randomized and observational studies (Virk et al., Lancet Microbe, 2024; Enne et al., Intensive Care Medicine, 2025; Chen et al., 2024; Plattner et al., 2024; Manda et al., 2025), which likewise reported that syndromic molecular panels more readily support escalation than de-escalation of antimicrobial therapy.

Accordingly, the Discussion and Conclusions were revised to emphasize that the BioFire FilmArray Pneumonia Panel should be interpreted as a complementary diagnostic tool to support antimicrobial stewardship, rather than as a stand-alone strategy for reducing antibiotic exposure.

We recognize that corrections of this magnitude require complete transparency. We have therefore deposited the fully de-identified analytic dataset together with the complete analysis script in a public repository, enabling independent verification of every value reported in the revised tables.

# Academic Editor

**Comment**

"The submitted article addresses a relevant topic. However, it requires further methodological clarification, as well as a better definition of the role of molecular diagnostics versus traditional diagnostics."

### Response: We appreciate the Academic Editor's thoughtful comments.

We have completely rewritten the **Statistical Analysis** section to improve methodological clarity. Detailed responses are provided under **Reviewer #2 (Comments 2 and 4)** and **Reviewer #3 (comments regarding lines 141 and 153–158)**. We also revised the Discussion and Conclusions to more clearly distinguish the diagnostic role of the FilmArray Pneumonia Panel from its real-world clinical impact on antimicrobial prescribing.

Completed STROBE (clinical-impact analysis) and STARD 2015 (diagnostic-performance analysis) reporting checklists, together with a participant flow diagram, have been included as Supporting Information.

In addition, the Discussion has been revised to provide a clearer distinction between the respective roles of the **BioFire FilmArray Pneumonia Panel** and conventional culture. Specifically, we emphasize that the FilmArray panel facilitates earlier, result-guided initiation of antimicrobial therapy, but did not reduce overall antibiotic exposure or increase de-escalation compared with conventional culture in the present study. We also discuss the respective strengths and limitations of the two diagnostic methods.

Moreover, we changed the title from "**Clinical Performance** and Clinical Impact..." to "**Diagnostic Performance** and Clinical Impact..." because the word ‘**clinical**’ appeared twice in the original title. Importantly, the term **"sputum culture group"** was misleading because it included both sputum and bronchoalveolar lavage (BAL) specimens. Therefore, throughout the revised manuscript, this term has been replaced with **"pre-implementation period."** Accordingly, **"FilmArray group"** has been renamed **"FilmArray period"** to maintain consistent terminology.

**Data availability**

*“We note that you have indicated that there are restrictions to data sharing for this study. PLOS only allows data to be available upon request if there are legal or ethical restrictions on sharing data publicly.”*

**Response:** The fully de-identified minimal dataset needed to reproduce every value in Tables 1, 4 and 5, together with the complete analysis script, has been deposited in a public repository and the Data Availability Statement has been updated accordingly. The fully de-identified dataset together with the complete statistical analysis script has been deposited in Zenodo (DOI: <https://doi.org/10.5281/zenodo.21767056>).

**Acknowledgements**

We have revised the **Acknowledgements** section to include the following statement:

"We thank Myeongjin Seo, MT, for microbiological data curation, and Seung-Hee Shin, Health Information Management Specialist, for assistance with medical record review and patient identification."

**This revision appropriately acknowledges their contributions to the study.**

**Reviewer #1**

**Comment 1 — Operational definition of antibiotic modification**

*"Antibiotic modification is defined quite broadly as 'escalation, de-escalation, or any change in regimen.' This definition makes it impossible to distinguish whether a clinician made a decision based on FilmArray findings or on concurrent biochemical, radiological, or other clinical data. Distinguishing modifications that can truly be attributed to FilmArray from this broad set requires a review of both the operational definition and the analytical method."*

**Response:** We agree, and have revised both the operational definition and the analytical method.

The primary clinical-impact endpoint is now a result-driven antibiotic action, defined a priori as the initiation of a new antibiotic, or a change to an existing regimen, occurring on the day of, or within five days after, the final microbiological result was reported. Antibiotic actions preceding the report were not counted. A "one day before the report" window that had been permitted for the FilmArray group in the original analysis has been removed, so that the temporal rule is now identical in both groups. The definition, and the number of patients meeting it in each group, are stated in the Methods and footnoted in Tables 4 and 5.

We also now report what kind of action was taken (Table 5): initiation of therapy in an antibiotic-naive patient versus modification of an ongoing regimen, and, among modifications, escalation versus de-escalation. This distinction proved to be the single most informative finding of the study.

We nevertheless accept the reviewer's deeper point: a temporal rule, however strict, cannot establish that the clinician acted because of the panel result rather than because of concurrent clinical, biochemical or radiological information. Our electronic records did not contain a structured field recording the clinician's stated reason for each antibiotic decision, so a formal attribution analysis was not possible. We have removed all language implying attribution, and have added the following to the Limitations:

*"*Although the FilmArray Pneumonia Panel provides rapid microbiological information, antibiotic prescribing decisions are influenced by multiple clinical factors in addition to microbiological test results. Clinical improvement or deterioration, radiologic findings, inflammatory markers, and other patient-specific considerations may all contribute to treatment modification. Therefore, although antibiotic actions were evaluated only when they occurred within five days of result reporting, this temporal association should not be interpreted as establishing a causal relationship between FilmArray implementation and changes in antibiotic prescribing.*."*

**Comments 2 and 3 — Validation of AMR genes against phenotypic susceptibility testing**

“*the detection of AMR genes was not validated by conventional susceptibility test results significantly weakens the clinical interpretation of resistance gene data. For example, it is unknown whether treatment was actually changed after KPC positivity was detected. This shortcoming significantly limits the study’s claims regarding its impact on antimicrobial stewardship. It is not specified whether these resistance genes have been investigated in pathogens cultured in vitro. If they have not been investigated, the fact that this comparison was not made appears to be a shortcoming*.”

Response: We agree that validation of antimicrobial resistance (AMR) genes against phenotypic antimicrobial susceptibility testing (AST) would provide additional information regarding the clinical significance of the detected resistance genes. However, **this analysis** was beyond the scope of the present study. The primary objectives of this study were to evaluate the diagnostic performance of the BioFire FilmArray Pneumonia Panel compared with conventional culture and to assess its clinical impact on antibiotic management. Accordingly, we did not perform a systematic correlation analysis between AMR gene detection and phenotypic AST results. **This limitation has been acknowledged in the revised manuscript, and we suggest that future studies specifically designed to investigate the concordance between molecular resistance determinants and phenotypic susceptibility are warranted.**

**Reviewer #2**

## Comment 1 — Uncontrolled before-and-after design; causal language

"...The authors should consider reframing all conclusions to reflect the observational nature of the study. Replace causal language (e.g., 'FilmArray testing was associated with...') with cautious, descriptive language (e.g., 'In this before-and-after comparison, the FilmArray group exhibited...')."

### Response: We fully agree with the reviewer's valuable comment and have revised the manuscript accordingly. Throughout the manuscript, statements implying a causal effect of the FilmArray Pneumonia Panel have been replaced with descriptive language appropriate for an observational before-and-after study. For example, expressions suggesting that the panel "led to" or "resulted in" changes in antibiotic management have been revised to describe observed differences between the two study periods without implying causality.

The **Abstract Conclusions**, **Discussion**, and **Conclusions** have all been rewritten to explicitly acknowledge that the study design does not permit causal inference. In addition, we have expanded the **Limitations** section to discuss potential temporal confounding, including secular changes in clinical practice, antimicrobial stewardship activities, patient case-mix, seasonal variation in pathogen prevalence, and institutional policy changes that may have influenced the observed findings.

## Comment 2 — Baseline imbalance and the absence of multivariable analysis

"...the FilmArray group is significantly younger, has higher CRP levels..., and differs in ICU proportion and residence distribution. Currently, the authors rely solely on univariate comparisons (t-tests and chi-square). The absence of multivariable analysis is a critical omission. Perform multivariable regression models (logistic or linear) or propensity score matching to adjust for these baseline imbalances (age, CRP, ICU status, residence)."

### Response: We appreciate this important comment, which prompted a comprehensive re-analysis of all clinical-impact outcomes and substantially strengthened the statistical analyses presented in the revised manuscript.

All clinical-impact outcomes are now reported as **adjusted** estimates. **Multivariable models** included the four variables identified by the reviewer (age, CRP, ICU admission, and residence type), together with sex and ECOG performance status. Continuous outcomes were analyzed using multivariable linear regression with heteroscedasticity-consistent (HC3) standard errors, whereas binary outcomes were analyzed using multivariable logistic regression.

Because IPTW was performed as a sensitivity analysis to assess the robustness of the primary multivariable analyses, the detailed IPTW results were not included in the manuscript.

| **Outcome** | **Multivariable regression** | **IPTW (propensity score)** |
| --- | --- | --- |
| Result-driven antibiotic action | OR 1.86 (1.24–2.79), P = 0.003 | OR 1.80 (1.20–2.69), P = 0.004 |
| Time to antibiotic action, days | −0.59 (−1.07 to −0.10), P = 0.019 | −0.57 (−1.01 to −0.13), P = 0.011 |
| Total antibiotic days | +1.86 (+0.29 to +3.44), P = 0.021 | +1.66 (+0.00 to +3.32), P = 0.050 |
| Length of hospital stay, days | −1.61 (−3.07 to −0.15), P = 0.031 | −1.77 (−3.32 to −0.22), P = 0.025 |
| 30-day mortality | OR 1.50 (0.90–2.48), P = 0.118 | OR 1.46 (0.92–2.32), P = 0.105 |

During the re-analysis, we identified an additional technical issue, which we report here for transparency. The residence variable originally included a nursing-home category containing only two patients in the entire cohort (one in each study group). Retaining this category as an independent model term produced a leverage value close to 1 and unstable heteroscedasticity-consistent standard errors. We therefore combined this category with the clinically adjacent long-term-care category, resulting in a three-level residence variable (home, long-term care, and acute-care hospital). This modification is described in the **Statistical Analysis** section and produced more stable model estimates and statistically reliable confidence intervals.

The reviewer specifically asked whether the observed findings remained after adjustment. The revised analyses provide a nuanced answer. The association between the FilmArray implementation period and **result-driven antibiotic decisions** as well as **earlier antibiotic modification** remained statistically significant after adjustment. In contrast, the apparent reduction in **total antibiotic exposure** observed in the original analysis was no longer supported; instead, adjusted analyses showed a modest but statistically significant increase in total antibiotic days. Likewise, the reduction in hospital length of stay persisted after adjustment but was considerably smaller than originally reported (adjusted reduction, 1.6 days versus the unadjusted estimate 5.0 days; P = 0.031 by multivariable regression, P = 0.025 by IPTW, and P = 0.038 in the survivor-only analysis).

## Comment 3 — The day 1–7 paradox

"The manuscript reports a decrease in overall antibiotic days in the FilmArray group, yet paradoxically reports longer antibiotic exposure during the first seven days (5.41 vs. 4.62 days, p = 0.012). This contradiction undermines the central narrative. Please provide an explicit discussion and explanation for this paradox."

### Response: We appreciate this insightful observation. The **apparent paradox** has now been resolved through the revised analyses.

The contradiction resulted from an inconsistency in the derivation of total antibiotic days, as described in the **General Response**. After recalculating antibiotic exposure using identical operational definitions in both study groups, the apparent contradiction disappeared. Antibiotic exposure was consistently higher in the FilmArray group during both the first seven days and the entire hospitalization.

The revised analyses also provide a plausible explanation for these findings. As shown in the **Table 5**, 67.9% of result-driven antibiotic actions in the FilmArray group represented **initiation of therapy in previously antibiotic-naïve patients**, compared with only 12.8% in the conventional culture group (P < 0.001). In contrast, de-escalation occurred at similar frequencies in both groups (30.8% vs. 35.3%; P = 0.81).

Taken together, these findings indicate that the FilmArray panel facilitated **earlier and more microbiologically informed antibiotic decisions**, but it did not shorten overall antibiotic exposure because clinicians more often used the results to initiate or broaden antimicrobial therapy rather than to de-escalate treatment. We have added a dedicated paragraph to the **Discussion** to explain this finding and revised the **Conclusions** accordingly.

## Comment 4 — Confidence intervals and effect sizes

## “*While CIs are mentioned in the abstract, the main results (Table 4) only report mean values and p-values. Report 95% CIs in all main results tables and consider adding effect sizes for continuous outcomes to improve the interpretability of the findings*.”

### Response: Thank you for this helpful suggestion. The manuscript has been revised accordingly. Every outcome reported in the revised **Table 4** now includes the number of patients contributing to the analysis, mean ± standard deviation, median (interquartile range), and the adjusted effect estimate (mean difference or odds ratio) with its corresponding 95% confidence interval.

The Statistical Analysis section has been revised to clarify the statistical methods used. Continuous outcomes were assessed for normality using the Shapiro–Wilk test. Because the primary analyses were based on adjusted multivariable regression models, only adjusted effect estimates with corresponding 95% confidence intervals are presented in the revised Table 5.

## Comment 5 — STARD and STROBE

### Response: We appreciate the reviewer's suggestion. Completed **STROBE** (for the clinical-impact analysis) and **STARD 2015** (for the diagnostic-performance analysis) checklists have been included as Supporting Information, together with a STROBE participant flow diagram.

### To comply with the STARD 2015 reporting guidelines, we added three sentences to the Methods section regarding (1) the reference standard, (2) blinding, and (3) sample size.

### 1) Line 160. Because this was a retrospective observational study, all eligible patients identified during the predefined study period were included, and no formal sample size calculation was performed. FilmArray testing and conventional microbiological culture were performed independently as part of routine clinical practice.

2) Line 161. FilmArray testing and conventional microbiological culture were performed independently as part of routine clinical practice, and no additional interpretation of either test was performed with knowledge of the corresponding results from the other method.
3) Line 131. Because of the retrospective study design, no formal sample size calculation was performed; instead, the sample size was determined by the number of eligible patients identified during the predefined study period.

**Reviewer #3**

1) “*The utility and performance characteristics of the tests viral targets are mentioned only in passing, and not sufficiently quantified or discussed. Without adding information on these, the article provides only a narrow and biased view of the tests clinical utility. For example how did a positive result on a viral target affect antibiotic de-escalation? And importantly; how well did Filmarray perform against reference tests for viral targets, and which tests were used as reference tests?*”

Response: We appreciate the reviewer's thoughtful comment. We agree that evaluation of the viral targets would require comparison with an appropriate reference method and assessment of their potential impact on clinical decision-making.

However, the present study was designed to evaluate the diagnostic performance of the BioFire FilmArray Pneumonia Panel for **bacterial pathogens** and its clinical impact on **antibiotic management**. Routine laboratory testing for respiratory viruses was not generally performed during the study period; therefore, no independent reference standard was available for assessing the diagnostic performance of the viral targets.

Likewise, the contribution of viral target detection to antibiotic de-escalation could not be reliably evaluated because antibiotic prescribing decisions were based on the overall clinical assessment, including patients' clinical presentation, radiologic findings, and microbiological results, rather than viral detection alone.

**Accordingly, diagnostic performance analyses were intentionally restricted to bacterial pathogens, and evaluation of the viral targets and their influence on antibiotic management was beyond the scope of the present study. We have clarified this point in the revised Methods, Results, and Discussion.**

2) “*For the bacterial targets, the comparison of clinical impact between the two study periods was only done with sputum samples, if I understood correctly. The authors should either include culture results for BAL samples prior to the adoption of Filmarray assay as well, or explain why this information is not available.*
Response: We appreciate the reviewer's important comment. This concern arose from our original use of the term **"sputum culture group,"** which was misleading. The clinical-impact analysis was **not** restricted to sputum specimens. Both sputum and bronchoalveolar lavage (BAL) specimens were included in the pre-implementation period and were evaluated using conventional culture, while the post-implementation period included the same specimen types evaluated using the FilmArray Pneumonia Panel in addition to conventional culture.”

To avoid this misunderstanding, we have revised the terminology throughout the manuscript. The comparator group is now consistently referred to as the **pre-implementation period**, rather than the **sputum culture group**, and we have clarified in the Methods that both sputum and BAL specimens were included in the clinical-impact analysis.

3) “*The routine culture methods and bacterial identification methods for culture are not described in the article. For the reader to adequately estimate the performance characteristics of the multiplex PCR test, more information on the reference testing methods is required*.”

Response: We agree that a more detailed description of the conventional microbiological methods is necessary to allow readers to appropriately interpret the diagnostic performance of the FilmArray Pneumonia Panel.

Accordingly, we have expanded the Methods section to describe the routine culture procedures used for respiratory specimens, including specimen processing, bacterial culture methods, organism identification, and antimicrobial susceptibility testing. We believe that these additional methodological details improve the transparency and reproducibility of the study.

4) “*Please verify that the article fulfills the STARD 2015 items*.”

### Response: Completed **STROBE** (for the clinical-impact analysis) and **STARD 2015** (for the diagnostic-performance analysis) checklists have been included as Supporting Information, together with a STROBE participant flow diagram. No substantive changes to the manuscript text were required because the reporting checklists are provided as supplementary files.

## Abstract, lines 49–50 — Reference point for time to antibiotic change

"Please confirm whether the time to change is from test result (as is stated in the abstract and later in the text) or from the time from sample collection or time from sample arriving to laboratory."

### **Response We thank the reviewer for this careful observation. The reviewer correctly identified an inconsistency in our original analysis, and this question led us to uncover a genuine error.**

The reported data of **3.83 days** for the sputum culture group had in fact been measured from **specimen collection**, whereas the corresponding FilmArray value had been measured from **result reporting**. Because the median turnaround time of sputum culture at our institution is **3 days**, compared with **0 days** for the FilmArray Pneumonia Panel, **this inconsistency accounted for most of the apparent difference between the two groups.**

Both groups are now measured from the same reference point—the reporting of the final microbiological result. Using this consistent definition, the mean time to a result-driven antibiotic action was **1.03 days** in the sputum culture group and **0.47 days** in the FilmArray group (adjusted difference, **−0.59 days**, 95% CI **−1.07 to −0.10**; P = 0.019). **A statistically significant difference therefore remains, although the magnitude of the effect is substantially smaller than originally reported.** The **Abstract**, **Results**, **Table 5**, and **Conclusions** have all been revised accordingly.

## Abstract, lines 50–51 — Escalation / de-escalation wording

## 50-51 “*The phrase describing escalation of sixty percent and de-escalation of thirty percent can seem unclear to the reader. Consider revising either adding detail and clarity, or simplifying to just stating there was no significant difference in the patterns of escalation or de-escalation, and provide the additional detail in the article*.”

### **Response:** We adopted the reviewer's second suggestion. The Abstract now states that, among patients whose ongoing antibiotic regimen was modified, the proportions of escalation and de-escalation did not differ significantly between the two study periods, with the complete distribution of antibiotic actions presented in Table 5.

**Definitions of escalation and de-escalation**

*"Please provide definitions for antibiotic escalation and antibiotic de-escalation that were used in the study."*

**Response:** These definitions have now been added to the **Methods**. Escalation was defined as a change to an antimicrobial agent or combination with a broader antibacterial spectrum than the regimen currently in use, including the addition of a new agent. De-escalation was defined as a change to a narrower-spectrum regimen or discontinuation of one or more agents from combination therapy without broadening antibacterial coverage.

Initiation of antibiotic therapy in patients who were not receiving antimicrobial treatment at the time of microbiological result reporting is now reported separately in **Table 5** as **"result-guided initiation"**, rather than being classified as escalation, **because no existing antibiotic regimen was available for comparison**

127-128 “*A table or a figure demonstrating the concordance (or lack of concordance) between BAL and sputum results from individuals who had both sample materials available would be an interesting addition to the paper*.”

Response: We appreciate the reviewer's thoughtful suggestion. We agree that a comparison of the concordance between bronchoalveolar lavage (BAL) and sputum results in patients who had both specimen types available would be of interest and could provide additional insight into the diagnostic performance of the FilmArray Pneumonia Panel. However, this analysis was beyond the scope of the present study, which was designed to compare the overall diagnostic performance of the FilmArray Pneumonia Panel with conventional culture and to evaluate its clinical impact on antibiotic management. Therefore, we did not perform this additional analysis. We agree that such an analysis would be valuable and consider it an important topic for future investigation.

141 “*Are these the intended references? Their absolute relevance for the diagnostic performance analysis seems questionable, ie these publications are hardly the first to describe the use of sensitivity, specificity, PPV and NPV, in the way they are utilized in the current study. In addition, there are differences between these two references, (for example in their approach to discrepant analysis), which makes clearer explanation of the approaches selected for this current study even more essential. As far as I can deduce from the tables, the sensitivity and specificity are calculated based on worst case scenario (ie. Assuming the culture result is always more ”correct” than the Filmarray result, possibly underestimating the PCR tests performance?)? If so, this could be stated explicitly. Consider revising this section to provide the reader clarity on your rationale and methodology, and additionally verify that the references are correct*.”

Response: We appreciate this insightful comment and agree that the rationale for our analytical approach, together with the methods used to calculate the diagnostic performance measures, should be described more explicitly.

In the revised manuscript, we have clarified that diagnostic performance was evaluated using conventional culture as the reference standard, and that sensitivity, specificity, positive predictive value (PPV), and negative predictive value (NPV) were calculated based on standard 2 × 2 contingency tables. We did not perform discrepant analysis or any adjudication of discordant results. Consequently, FilmArray-positive/culture-negative results were classified as false-positive results and FilmArray-negative/culture-positive results as false-negative results for the purpose of calculating diagnostic performance. As the reviewer correctly noted, this represents a conservative ("worst-case") approach that may underestimate the diagnostic performance of the molecular assay.

Because the statistical methods used are standard and self-explanatory, we removed the previous references and instead expanded the methodological description to explicitly explain how diagnostic performance was calculated.

147 ”*According to the above period.. I suggest stating the specific time period, as it is laborious for the reader to go back to the beginning to check it.”*

**Response:** We appreciate the reviewer's helpful suggestion. The manuscript has been revised to state the specific study period directly, rather than referring to "the above period," thereby improving clarity and readability.

149 ”*Were categorized to the sputum culture group..” Meaning no BAL samples were included in the culture group? The lack of BAL samples in the ”pre-Biofire” group/comparator group should be addressed, see also major revision 2*.”

Response: We appreciate the reviewer's careful and very important observation. We agree that the original wording, **"sputum culture group,"** was misleading and could be interpreted as indicating that only sputum specimens were included in the comparator group. This was not the case. The pre-implementation period included both sputum and bronchoalveolar lavage (BAL) specimens, all of which were evaluated by conventional culture.

To avoid this misunderstanding, we have revised the terminology throughout the manuscript. The comparator group is now consistently referred to as the **pre-implementation period**, rather than the **sputum culture group**. **Similarly, the "FilmArray group" has been renamed the "FilmArray period" to maintain consistent terminology.** We have also clarified that both sputum and BAL specimens were included during the pre-implementation period, whereas the post-implementation period included the same specimen types evaluated using the FilmArray Pneumonia Panel in addition to conventional culture.

## Methods, lines 153–154 — Inclusion and exclusion criteria

## 153-154 “*The Filmarray time period for the clinical impact comparison was defined as same as the diagnostic performance evaluation, but here is stated that only 200 samples were included in the Filmarray group. Please clarify in the article, what were the inclusion/exclusion criteria here*?”

**Response:** We apologize that these eligibility criteria were not clearly described in the original submission. They are now stated explicitly in the Methods, and a STROBE participant flow diagram has been added as Supporting Information to illustrate the number of patients excluded at each step.

Both groups comprised hospitalized patients with pneumonia who (i) received antibiotic therapy during admission and (ii) had a positive microbiological result—a bacterial pathogen detected by sputum culture or a bacterial target detected by the FilmArray panel. Patients were excluded if they had not received antibiotics, had a negative microbiological result, or—**during the FilmArray period**—had only atypical bacteria or viral targets detected.

**The rationale for the final exclusion criterion has now been clarified in the manuscript.** Conventional sputum culture cannot detect respiratory viruses; therefore, an equivalent "virus-only" category does not exist in the comparator group. Including such patients only in the FilmArray group would have introduced a population with, by definition, no microbiological indication for antibacterial therapy into one study arm but not the other, thereby biasing all antibiotic-related comparisons. Excluding these patients ensured that the two cohorts were comparable with respect to the microbiological indication for antibiotic treatment. These criteria yielded **294 patients during the pre-implementation period** and **200 patients during the FilmArray period**.

We have also clarified at the beginning of both Results subsections that the diagnostic accuracy analysis (558 specimens) and the clinical-impact analysis (494 patients) were conducted in different study populations.

## Methods,

## 158-159 “*Please confirm whether the time to antibiotic change is from test result, or from the time from sample collection or time from sample arriving to laboratory. If it is truly from reporting the test result, the mean times, especially for the sputum culture group, in the results section seem rather long and in that case would be beneficial to discuss, whether antibiotic changes this long after reporting the results are related to the laboratory results or are they attributable to other factors*.”

**Response:** The reviewer correctly inferred that the reported interval had **not** been measured from result reporting (see our response above).

Using a consistent definition based on result reporting, the mean interval to a result-driven antibiotic action was **1.03 days** in the pre-implementation period and **0.47 days** in the FilmArray period, while the median was **0 days** in both groups. The implausibly long intervals that prompted the reviewer's question **have therefore been eliminated.**

Furthermore, antibiotic actions occurring more than five days after result reporting are no longer considered result-driven, precisely because such decisions are increasingly likely to reflect subsequent clinical developments rather than the microbiological result itself. This rationale is now explicitly described in the Methods and **further discussed** in the Limitations.

“*In Table 5 the total number of samples includes only the patients receiving antibiotic treatment. As this number is notably lower than the total number of samples in each group, it appears evident that many of the samples that were positive for bacterial pathogens in Filmarray testing were from patients that were not clinically considered true bacterial infections (only 81 from 200 patients in the Filmarray group were receiving antibiotics according to this table; In patients considered clinically as having bacterial pneumonia, antibiotic treatment would have undoubtedly been initiated). I highly recommend exploring this (patients positive for bacterial pathogens, but not receiving antibiotic treatment) in the results and discussion*.”

Response: We appreciate the reviewer's careful observation. The apparent discrepancy results from a misunderstanding of the denominator used in Table 5. Table 5 does **not** include all patients in the clinical-impact cohort or all patients who received antibiotic therapy. Rather, it includes **only patients whose antibiotic regimen was modified after the microbiological result became available**. Patients whose antibiotic treatment remained unchanged are not included in this table.

Therefore, the number of patients shown in Table 5 should not be interpreted as the number of patients who received antibiotic treatment or as evidence that bacterial pathogen-positive patients were not treated with antibiotics. To avoid this misunderstanding, we have revised the table title and footnotes to clarify that Table 5 summarizes **only patients who underwent a microbiology-guided antibiotic modification**.

“*The method section seems to lack clear information on additional testing that the patients with Biofire Filmarray testing results underwent. Later in the results section it is clear that most these patients underwent conventional culture as well, but this should be described in the materials and methods section as well. For example details on whether selective culture for Legionella species (or urine antigen testing for Legionella pneumophila) was performed on any subset of the patients, and so on for other pathogens, as this would help assess the performance of the multiplex test for different contexts*.”

Response: We agree that the additional microbiological testing performed during the FilmArray period should be described more clearly in the Methods.

In the revised manuscript, we have clarified that patients in the FilmArray period also underwent conventional microbiological culture as part of routine clinical care, and that the diagnostic performance analysis was based on the comparison between FilmArray results and conventional culture. We have also clarified that additional pathogen-specific investigations, such as selective culture for Legionella species or urinary antigen testing for Legionella pneumophila, were **not performed systematically according to the study protocol** and therefore were not incorporated into the diagnostic performance analysis. Consequently, these tests were not used as reference standards for evaluating the performance of the FilmArray Pneumonia Panel.

These clarifications have been added to the Methods section to improve the transparency of the study design and microbiological evaluation.

“*If available, results for blood cultures from the patients would also be of great interest, and clarify the possible additional value of the Biofire Pneumonia panel in pneumonia patients with positive blood cultures*.”

Response: We agree that blood culture results could provide additional information regarding the potential complementary value of the FilmArray Pneumonia Panel in patients with bacteremic pneumonia. However, the present study was designed to evaluate the diagnostic performance of the FilmArray Pneumonia Panel using respiratory specimens and to assess its clinical impact on antibiotic management. Accordingly, blood culture results were beyond the scope of the current study and were not included in the study analyses. We agree that integrating respiratory and blood culture findings would be of considerable interest and represents an important direction for future research.

“*Importantly, comparison to other detection methods for viral pathogens is lacking. Please describe what were the reference methods used in the laboratory to evaluate the performance of the viral targets of the Biofire Filmarray assay. See also major revision note 1*.”

Response: We agree that evaluation of the viral targets would require comparison with an appropriate reference method, such as a validated respiratory viral PCR assay. However, routine laboratory testing for respiratory viruses was not generally performed in the study population, and therefore no independent reference method was available for evaluating the viral targets detected by the FilmArray Pneumonia Panel.

**Table 4 — Number of patients in each row**

*"Table 4: Please include in the table or in the text description what was the total number of patients on each row."*

**Response:** Thank you for this helpful suggestion. The manuscript has been revised accordingly. Every row of the revised Table 5 states its analysis population and the number of patients contributing to it in each group. The footnotes also state how many patients were removed by each pre-specified truncation (antibiotic courses > 45 days: 14 in the culture group and 15 in the FilmArray group; hospital stays > 30 days: 94 and 43 respectively), and we now report a sensitivity analysis in which the 45-day truncation is removed. The conclusion is unchanged (adjusted ratio of means 1.37, 95% CI 1.16–1.62; P < 0.001, log-transformed model).

**Handling of deaths in the length-of-stay analysis**

*"Please explain, how were deaths handled in the analysis of hospital stay length? Excluded from analysis?"*

**Response:** Patients who died in hospital were included; their length of stay was the interval from admission to death. This is now stated explicitly in the Methods. Because in-hospital death shortens length of stay, and because 30-day mortality differed numerically between the groups, we repeated the analysis restricted to survivors as a sensitivity analysis. The estimate was essentially unchanged (adjusted difference −1.62 days, 95% CI −3.16 to −0.09; P = 0.038, versus −1.61 days, 95% CI −3.07 to −0.15; P = 0.031 in the primary analysis). We also now report 30-day mortality, which was omitted from the original Table 4: 19.8% in the sputum culture group and 23.1% in the FilmArray group (adjusted OR 1.50, 95% CI 0.90–2.48; P = 0.118).

### Results:

## Figures

## “*Figures present only percentages, consider adding a table of the Filmarray findings that were missed by culture and vice versa.*”

### **Response:** We thank the reviewer for this valuable suggestion. In response, we removed the previous figure showing concordance percentages between conventional culture and the FilmArray Pneumonia Panel. **It has been replaced by two new tables (Tables 2A and 2B)** presenting organism-level discordant results, including pathogens detected only by the FilmArray panel (culture-negative/FilmArray-positive) and those detected only by conventional culture (culture-positive/FilmArray-negative), separately for BAL and sputum specimens.

These tables provide substantially more clinically relevant information regarding discordant findings between the two diagnostic methods. The **Results** section has been revised accordingly, and the previous **Figure 1** has been removed.

Tables.
“*Consider if Table 3 is necessary? And were all multiple findings considered significant ie.true polymicrobial infections? Any confirmatory testing performed, or additional evaluation of clinical presentation? Personally I would find it more interesting to explore the clinical significance of multiple findings, rather than the number of possible pathogens detected*.”
**Response:** We agree that the clinical significance of multiple pathogen detections is more important than simply reporting the number of organisms detected. Accordingly, **Table 3** has been **removed** from the revised manuscript.

In the present retrospective study, we did not consider all multiple detections to represent true polymicrobial infections. No confirmatory microbiological testing or standardized clinical adjudication was performed to determine the clinical significance of each detected organism. Therefore, distinguishing true infection from colonization or clinically insignificant findings was beyond the scope of this study.

We have acknowledged this limitation in the Discussion and stated that future prospective studies integrating microbiological, radiological, and clinical data are warranted to better evaluate the clinical significance of multiple pathogen detections.

275 and Table 5: “*For antibiotic modification, were other factors considered, for example modifications based on clinical improvement/deterioration? Consider adding a flowchart to clarify escalation and de-escalation that different patient groups underwent. Ideally the flow chart would include information on the type of result that prompted the modification, or a categorical evaluation on whether the modification was truly warranted based on the result, and whether there had been results, that would have warranted escalation or de-escalation, but did not result in changes in patient management*.”

Response: We appreciate the reviewer's thoughtful and constructive suggestion. We agree that a detailed evaluation of the clinical factors influencing antibiotic modification, including whether changes were prompted by microbiological results, clinical improvement or deterioration, or other clinical considerations, would provide additional insight into antimicrobial decision-making.

However, the present study was designed to compare antibiotic modifications between the pre-implementation and FilmArray periods rather than to adjudicate the clinical appropriateness of individual treatment decisions. Because of the retrospective study design, it was not possible to reliably determine the relative contribution of microbiological results versus other clinical factors to each antibiotic modification, nor to objectively assess whether changes were warranted or whether opportunities for escalation or de-escalation were missed.

We agree that a flowchart incorporating these clinical decision pathways would be valuable. However, such an analysis was beyond the scope of the current study and would require detailed prospective clinical adjudication. We have acknowledged this as a limitation in the revised manuscript and consider it an important area for future research.

“*The comparison of the demographic details between the study groups is presented in Supplementary file 3. As this is important information consider transferring it to the main article*.”

Response: We agree that comparison of baseline demographic characteristics is essential for interpreting the clinical-impact analyses. Accordingly, the table previously provided as **Supplementary File 3** has been moved into the main manuscript and is now presented as **Table 4**.

To improve clarity throughout the manuscript, we have also revised the terminology used to describe the study groups. The previous labels, **"sputum culture group"** and **"FilmArray group,"** have been replaced with **"pre-implementation period"** and **"FilmArray period,"** respectively. These revised terms more accurately reflect the study design because both sputum and bronchoalveolar lavage specimens were included during the FilmArray period, whereas the previous terminology could incorrectly suggest that only sputum specimens were analyzed.

DISCUSSION

318-319 “*Consulting the list of bacteria for this group shows that most of them appear to be true pathogens. Please clarify, what do the authors mean by this phrase?*”

Response: We thank the reviewer for pointing out this ambiguity. The original wording was imprecise. By this phrase, we intended to indicate that the bacteria detected by the FilmArray Pneumonia Panel in this group were organisms that are commonly recognized as respiratory pathogens, rather than microorganisms that are typically regarded as colonizers or contaminants. We have revised the text to clarify this point and to avoid overinterpretation of the results.

319 ”*In THE systematic review..”, I believe should be: ”in A systematic review*”

Response: Thank you for this helpful suggestion. The manuscript has been revised accordingly.

327 “*Could the authors refer here to surveillance data from their region*?”

**Response:** We have incorporated **regional antimicrobial resistance surveillance data from Gyeongsang National University Changwon Hospital collected during the same study period (2023)** to provide local epidemiological context for ESBL-producing Enterobacterales, carbapenem-resistant Enterobacterales, and methicillin-resistant Staphylococcus aureus. The **Discussion** has been revised accordingly.

331 “*Consider discussing the possibility of colonization, thus leading to unnecessary antibiotic escalation?*”

**Response:** We thank the reviewer for this valuable suggestion. We agree that the high analytical sensitivity of the FilmArray Pneumonia Panel may detect colonizing organisms that are not responsible for the patient's infection. If these findings are interpreted without appropriate clinical correlation, they could potentially lead to unnecessary initiation or escalation of antimicrobial therapy. We have therefore revised the Discussion to explicitly acknowledge this limitation and to emphasize that FilmArray results should always be interpreted together with the patient's clinical presentation, radiologic findings, and conventional microbiological results.

337-339: “*Consider discussing also what are the advantages of culture in comparison with molecular methods? Such as semi-quantitative nature, scope of detectable bacteria (and fungi) more comprehensive than in a targeted panel, possibilities for selective cultures and phenotypic susceptibility testing*?”
**Response:** We fully agree that the complementary strengths of conventional culture should be more clearly acknowledged.

Accordingly, the **Discussion** now emphasizes that, despite the rapid turnaround time of the FilmArray Pneumonia Panel, conventional culture remains indispensable because it provides semi-quantitative information that may help distinguish colonization from infection, detects a broader range of bacterial and fungal pathogens than those included in targeted molecular panels, enables selective culture for unexpected organisms, and supports phenotypic antimicrobial susceptibility testing. **We therefore emphasize that molecular assays should be regarded as complementary to, rather than replacements for, conventional microbiological methods.**

356-357 *”several studies”, but only one reference, consider adding other relevant references or rephrasing.*

**Response:** We changed the expression, ‘several studies’ into ‘a study’.

362-364 “*seems to contradict with 374-376, so please specify how you define the difference between antibiotic optimization and antibiotic modification*.”

**Response:** We agree that the term "antibiotic optimization"was too broad and could be interpreted inconsistently with "antibiotic modification."To avoid ambiguity, we have removed the term "antibiotic optimization". Instead, Table 5 and the corresponding text now explicitly distinguish "result-guided initiation of antibiotics"from "modification of an ongoing antibiotic regimen (including escalation and de-escalation)." We believe these revisions provide a clearer description of the clinical actions evaluated in this study.

366 “*I believe it would be important to evaluate whether the Filmarray results even would consistently enough warrant de-escalation to have a detectable effect on antibiotic prescription patterns? Ie if the Filmarray result is, for example polymicrobial (as it often is), it seems natural that the clinician would await for culture results and/or clinical improvement, before de-escalation. The authors could explore already in the results section, what is the proportion of truly actionable results in a real life scenario*.”
**Response:** We greatly appreciate this thoughtful and clinically important suggestion because it prompted us to examine an aspect of clinical utility that had not been sufficiently explored in the original manuscript.

We agree that not every positive FilmArray result is immediately actionable, particularly in patients with polymicrobial detections or when confirmation by conventional culture and correlation with the clinical presentation are required before narrowing antimicrobial therapy.

In our study, antibiotic management reflected real-world clinical practice rather than hypothetical treatment decisions. Clinicians interpreted FilmArray results together with patients' clinical status, radiologic findings, severity of illness, and subsequent culture results when making antimicrobial decisions. Consequently, not every positive FilmArray result led to immediate antibiotic de-escalation.

Because this was a retrospective study, we could not reliably classify individual FilmArray results as "actionable" or "non-actionable" using predefined criteria without introducing subjective interpretation. We therefore acknowledge this as an important limitation and recommend that future prospective studies evaluate both the proportion of actionable FilmArray results and their direct impact on antimicrobial stewardship interventions.

To address the reviewer's concern, we have expanded the **Discussion** to clarify that the clinical utility of the FilmArray Pneumonia Panel depends not only on its diagnostic performance but also on the interpretability and clinical actionability of individual test results. We also emphasize that polymicrobial detections and discordant findings may appropriately prompt clinicians to continue empirical therapy until additional microbiological or clinical information becomes available. Consequently, the observed impact on antibiotic prescribing reflects real-world clinical decision-making rather than the diagnostic performance of the assay alone.

410 *”Lack of reliability”, can the authors be more clear; does this refer to the clinicians lack of confidence in the results (if so, how was this evaluated or measured)? Or does it refer to the fact, that the absence of the genes included in the panel does not necessarily reflect phenotypic susceptibility?*

**Response:** We thank the reviewer for pointing out this ambiguity. Our intention was not to suggest that clinicians lacked confidence in the FilmArray results, as clinician confidence or perceptions were not evaluated in this retrospective study. **To avoid misinterpretation,** we have removed the phrase **"lack of reliability"** and replaced it with a more precise description that accurately reflects the scope of the present study. The revised wording now emphasizes the limitations of molecular resistance-marker detection rather than clinician confidence in the assay.

414-415 “*Can this statement be made with confidence? Is it shown in the article, that pathogens responsible for the clinical disease or AMR genes were missed by culture, especially when correlation with culture based susceptibility testing or culture based AMR screening was not included in the study? Consider revising to better reflect the actual results of the current study*.”

**Response:** We thank the reviewer for this important observation and agree that our original wording was stronger than warranted by the available evidence.

Our study did not directly evaluate whether pathogens responsible for the clinical infection or antimicrobial resistance determinants were missed by conventional culture. In addition, correlation between FilmArray resistance gene detection and culture-based antimicrobial susceptibility testing or comprehensive resistance mechanism analysis was beyond the scope of this study.

Accordingly, we have revised the **Discussion** to avoid implying that the FilmArray Pneumonia Panel definitively identified pathogens or resistance determinants missed by conventional culture. Instead, we now state that the FilmArray Pneumonia Panel **may provide complementary microbiological information** that can assist conventional diagnostic methods, while emphasizing that its clinical significance should always be interpreted together with conventional culture findings and the patient's clinical presentation.

We believe that the revised wording more accurately reflects both the findings and the limitations of the present study.

“*Because of the retrospective nature of the study, the differences observed between the timeframe before Filmarray use and during the Filmarray use may be due to various factors unrelated to the newly adopted methodology. It is mentioned briefly in the manuscript (principally in 354-355, 390-392), but I believe it warrants to be acknowledged as a significant limitation even more clearly*.”

**Response:** We thank the reviewer for this important comment and fully agree that the retrospective before-and-after study design represents one of the major limitations of the present study.

Because the pre-implementation period and the FilmArray implementation period occurred at different time points, temporal changes unrelated to the introduction of the FilmArray Pneumonia Panel may have influenced antibiotic prescribing practices and clinical outcomes. Potential sources of confounding include changes in antimicrobial stewardship activities, institutional treatment protocols, clinician experience, patient characteristics, and local epidemiology.

Accordingly, we have substantially expanded the **Limitations** section to emphasize that the observed differences between the two study periods cannot be attributed solely to implementation of the FilmArray Pneumonia Panel. We further state that **prospective controlled studies with concurrent comparison groups** are required to more accurately determine the causal impact of the FilmArray Pneumonia Panel on antimicrobial prescribing and patient outcomes.

“*Some inherent limitations of molecular methods, that I believe could be mentioned or discussed in relation to the results:
-Discussion of clinically insignificant findings due to high sensitivity of the assay, and the risks related to this.
-Scalability of the methodology at its current price point?*”

Response: We agree that these are important considerations when interpreting the clinical utility of multiplex molecular diagnostic assays.

Accordingly, we have expanded the **Discussion** to acknowledge two additional limitations of the FilmArray Pneumonia Panel.

First, because of its high analytical sensitivity, the assay may detect colonizing organisms, nonviable microorganisms, or clinically insignificant pathogens that are not responsible for the patient's infection. Therefore, FilmArray results should always be interpreted in conjunction with the patient's clinical presentation, radiologic findings, and conventional microbiological results to avoid unnecessary antimicrobial therapy.

Second, we acknowledge that the relatively high cost of multiplex molecular testing may limit its widespread implementation, particularly in resource-limited healthcare settings. Although earlier diagnosis and more appropriate antimicrobial therapy **may partially offset these additional costs**, formal cost-effectiveness analyses were beyond the scope of the present study. Future prospective studies should evaluate both the economic impact and the scalability of routine FilmArray implementation in different clinical settings.

These additional considerations have been incorporated into the revised **Discussion**.

**.** **Discussion, line 366 — Proportion of truly actionable results**

*"...The authors could explore already in the results section, what is the proportion of truly actionable results in a real life scenario."*

**Response:** We greatly appreciate this suggestion because it prompted us to examine an important aspect of clinical utility that had not been sufficiently addressed in the original manuscript.

The new **Table 6** now directly addresses this question by reporting both the proportion of microbiological results that led to an antibiotic action (**26.5% vs. 40.5%**) and the specific type of action (**result-guided initiation, escalation, or de-escalation**).

Importantly, de-escalation occurred in only **8 of 81 patients** in the FilmArray period, supporting the reviewer's **hypothesis** that polymicrobial molecular results alone rarely provide clinicians with sufficient confidence to narrow antimicrobial therapy. This finding is now discussed explicitly in the revised **Discussion**, where we emphasize that the clinical value of the FilmArray Pneumonia Panel lies not only in its diagnostic accuracy but also in how its results are integrated into real-world antimicrobial decision-making.

**Summary of changes to the manuscript (statistical sections)**

- **The Statistical Analysis section was completely rewritten** to include pre-specified operational definitions, normality assessment, parallel reporting of parametric and non-parametric analyses, multivariable regression models with predefined covariates, IPTW sensitivity analyses, handling of deaths and outcome truncation, statistical software, and explicit adherence to the STROBE and STARD reporting guidelines.
- **Table 4 (Baseline characteristics)** was moved from the Supplementary Material to the main manuscript. Standardized mean differences were added, and one implausible body mass index value (658.8 kg/m²) was corrected. The revised BMI values are **21.87 ± 3.88 kg/m²** and **21.63 ± 4.16 kg/m²**.
- **Table 5 (Clinical outcomes)** was completely reconstructed to report the analysis population and sample size for each outcome, mean ± SD and median (IQR), adjusted effect estimates with 95% confidence intervals, and 30-day mortality.
- **Table 6 (Result-driven antibiotic actions)** was newly structured to present the frequency and pattern of antibiotic actions (result-guided initiation, escalation, and de-escalation), together with explicit denominators and Fisher's exact test results.
- The **Abstract** and **Conclusions** were revised to reflect the corrected analyses and updated interpretation of the study findings.
- A **de-identified analytic dataset** together with the complete **statistical analysis script** has been deposited in a public repository to ensure transparency and reproducibility.
