# Supplementary material for: Diagnostic performance and clinical impact of the BioFire FilmArray Pneumonia Panel compared with conventional culture in hospitalized patients with pneumonia
Source: PLoS One. 2026 Sep 28;21(9):e0358986. doi: 10.1371/journal.pone.0358986 (PMC13618948; doi:10.1371/journal.pone.0358986)
Supplement: Supplementary file 7 [file pone.0358986.s009.docx]

**Response to Reviewers — second revision**

*PONE-D-26-13230R1*

*Diagnostic Performance and Clinical Impact of the BioFire FilmArray Pneumonia Panel Compared with Conventional Culture in Hospitalized Patients with Pneumonia*

**General response**

We thank Reviewer #3 for a second careful reading and for the generous assessment of our first revision. The two principal comments have again improved the paper substantially, and both have been fully addressed in the revised manuscript.

Comment 1 highlighted an important aspect that was not adequately addressed in our previous revision. Turnaround time is the central practical advantage of syndromic molecular testing, and our previous analysis, anchored entirely on the reporting of the result, could not show it.

Comment 2 identified an equally important framing problem: by restricting Table 6 to patients in whom some action followed the result, we had treated a deliberate decision not to change therapy as though it were not a decision at all. Both tables have been rebuilt. Measured from specimen sampling, the difference was large and statistically significant, and this finding is now emphasized in the revised manuscript.

We are glad to report that the reviewer’s own calculations were reproduced by our re-analysis, and that the reviewer’s substantive question about escalation and de-escalation has a clear answer, given below.

The reviewer’s minor points 3 to 6 have all been addressed, and one of them (point 6) prompted a correction to the supplementary figure that we are grateful to have caught before publication.

**Reviewer #3**

**Comment 1 — Time from sampling to result-driven action**

*"...an additional important comparison might in fact be made comparing time from sampling to result driven decision: the main advantage of molecular testing generally being faster turn-around time. Thus, an important additional correction would be to report also time from sampling to result driven decision for both groups... For clarity, I believe the current comparison of time from microbiologic result to action should also remain in the article."*

**Response:** We agree, and this comment has changed the emphasis of the paper. Two new rows have been added to Table 5, and the existing result-reporting comparison has been retained exactly as the reviewer requests, so that the two timings can be read side by side.

First, we now report the turnaround time itself — the interval from obtaining the respiratory specimen to reporting of the final microbiological result:

— Pre-implementation period: mean 3.18 ± 1.21 days, median 3 (IQR 2–4), n = 293

— FilmArray period: mean 0.02 ± 0.12 days, median 0 (IQR 0–0), n = 199

— Difference −3.17 days (95% CI −3.31 to −3.03); P < 0.001

Second, we report the interval from sampling to the result-driven antibiotic action:

— Pre-implementation period: mean 3.91 ± 1.73 days, median 4 (IQR 2–5), n = 78

— FilmArray period: mean 0.47 ± 1.23 days, median 0 (IQR 0–0), n = 81

— Unadjusted difference −3.44 days (95% CI −3.91 to −2.97); adjusted difference −3.53 days (95% CI −4.08 to −2.99); P < 0.001

The contrast between the two timings is itself informative, and we now make it explicitly in the Discussion. Measured from the reporting of the result, the panel advanced antibiotic decisions by about half a day; measured from the moment the specimen was taken, it advanced them by three and a half days.

The terminology has been made explicit throughout: "turnaround time" for sampling to reporting, "time from sampling to antibiotic action", and "time from result reporting to antibiotic action". Most of the observed time advantage therefore appears to derive from the shorter diagnostic turnaround time rather than from a faster clinician response after the result became available.

Turnaround time is presented unadjusted, since it is a property of the diagnostic method rather than of the patient; the two action-time outcomes are presented both unadjusted and adjusted.

**Comment 2 — Denominator for Table 6**

*"...I believe it would be more meaningful and informative to report the proportion of patients with specific modifications of all patients included in the clinical impact evaluation... Deciding not to change the antibiotic regimen after the microbiological result is still a clinical decision even if it appears ‘passive’... Crudely, both de-escalation and escalation appear to be more frequent in the pre-intervention period, (possible statistical significance?)"*

**Response:** We accept this point without reservation. Table 6 has been rebuilt on the whole clinical-impact cohort, and a row for patients in whom no action followed the result has been added. The reviewer’s calculations were confirmed by our re-analysis, with minor differences due only to rounding. The revised Table 6 is as follows (percentages are of 294 and 200 patients respectively):

— Result-guided initiation: 10 (3.4%) vs 55 (27.5%); P < 0.001; adjusted OR 9.20 (95% CI 4.48–18.90), P < 0.001

— Escalation: 44 (15.0%) vs 18 (9.0%); P = 0.053; adjusted OR 0.55 (95% CI 0.29–1.03), P = 0.061

— De-escalation: 24 (8.2%) vs 8 (4.0%); P = 0.092; adjusted OR 0.58 (95% CI 0.24–1.40), P = 0.226

— No action attributable to the result: 216 (73.5%) vs 119 (59.5%); P = 0.001; adjusted OR 0.54 (95% CI 0.36–0.81), P = 0.003

The four-category distribution differed between periods (chi-square P < 0.001).

**On the reviewer’s specific question — whether escalation and de-escalation were significantly more frequent before implementation — the answer is that the direction is as the reviewer observed, but neither difference reaches conventional significance, before or after adjustment. We now state this explicitly rather than leaving it to be inferred.**

This whole-cohort analysis provides a more clinically informative interpretation of the pattern of antibiotic actions, and we have revised the Results and Discussion accordingly. What changed with implementation of the panel was not the frequency of escalation or de-escalation but the character of the decision: result-guided antibiotic initiation was substantially more frequent among patients who were not receiving antibiotics at the time of microbiological reporting (3.4% vs. 27.5%), and the proportion of patients in whom the microbiological result changed nothing fell from 73.5% to 59.5%. At the same time, the fact that 59.5% of patients in the FilmArray period had no antibiotic action attributable to the microbiological result within five days, and that de-escalation remained uncommon in both periods, is now presented as a genuine limitation of real-world actionability rather than as a neutral observation. This is consistent with our observation of modestly increased total antibiotic exposure that we report, and with the randomized and observational literature we cite.

**Comment 3 — Abstract wording**

*"Abstract, lines 47-48: I recommend removing the indirect (“double-negative”) expression: “did not decrease” and substituting with more direct language: “increased antibiotic exposure”."*

**Response:** Changed as suggested. The sentence now reads:

"…However, total antibiotic exposure was modestly longer (adjusted mean difference, 1.86 days; 95% CI, 0.29–3.44; P = 0.021), and antibiotic de-escalation rates did not differ significantly between the two periods."

**Comment 4 — Role of molecular testing in the Abstract**

*"Abstract lines 49-50: I suggest adding something to further clarify the role of molecular testing, for example: “when used as a complementary tool to traditional microbiological methods and integrated with structured ...”"*

**Response:** Adopted. The concluding sentence of the Abstract now reads:

"The BioFire FilmArray Pneumonia Panel improved pathogen detection and facilitated earlier and more frequent microbiology-guided antibiotic management, and was associated with a modest reduction in hospital length of stay. However, it modestly increased overall antibiotic exposure and did not increase antibiotic de-escalation. Rapid molecular diagnostics may improve the timeliness and precision of antimicrobial therapy, but their full clinical benefit is likely to be realized only when they are used as a complementary tool alongside conventional microbiological methods and integrated within a structured antimicrobial stewardship program."

**Comment 5 — Missing 30-day mortality data**

*"Table 5: In the 30-day mortality comparison both groups are missing one patient. Based on Figure S1 this is missing data. Can the authors confirm whether this is correct?"*

**Response:** The reviewer is correct, and we thank them for checking. Vital status at 30 days could not be ascertained in exactly one patient in each period. In both cases the patient was transferred to another institution before day 30 and no further record was available to us. The reviewer is also right that this should be rare, and it is: 2 of 494 patients (0.4%).

We have added a footnote to Table 5 stating the reason, and the lower panel of S1 Fig now labels these two patients explicitly. The mortality comparison is therefore based on 293 and 199 patients. Assigning both patients to either outcome does not change the conclusion (all P > 0.1).

**Comment 6 — S1 Fig: culture of BAL samples**

*"Figure S1: Pre-implementation box only mentions sputum culture, where I believe, should be also culture of BAL-samples."*

**Response:** The reviewer is right and we are grateful for the correction. Bronchoalveolar lavage cultures were indeed included in the pre-implementation period; the label in S1 Fig was also inconsistent with the manuscript text, which refers throughout to conventional culture rather than to sputum culture alone. S1 Fig has been corrected: the period is now labelled "conventional culture" and the screening box reads "Patients with a respiratory culture (sputum or BAL) for suspected pneumonia". We have re-checked the remaining figure labels for the same error and found none.

**Summary of changes**

— Table 5: two new rows reporting turnaround time (sampling to result reporting) and time from sampling to antibiotic action, with the existing result-reporting comparison retained; footnote added on the two patients with unascertained 30-day vital status.

— Table 6: rebuilt on the whole clinical-impact cohort (294 and 200), with a row for patients in whom no action followed the result, Fisher exact P values, and adjusted odds ratios.

— Abstract: direct wording for the antibiotic-exposure finding; sampling-to-decision interval added; concluding sentence revised to describe the panel as a complementary tool within a stewardship program.

— Results and Discussion: revised to distinguish turnaround time from clinician response time, and to interpret the whole-cohort distribution of antibiotic actions, including the absence of a significant difference in escalation and de-escalation.

— S1 Fig: pre-implementation period relabelled as conventional culture of sputum or BAL; turnaround time added to the analysis-population panel.

— Statistical Analysis: definitions of the two sampling-based intervals added.
