# Supplementary material for: Diagnostic performance and clinical impact of the BioFire FilmArray Pneumonia Panel compared with conventional culture in hospitalized patients with pneumonia
Source: PLoS One. 2026 Sep 28;21(9):e0358986. doi: 10.1371/journal.pone.0358986 (PMC13618948; doi:10.1371/journal.pone.0358986)
Supplement: Peer review history

# Peer review: Decision Letter 0

**DOI:** 10.1371/journal.pone.0358986.r001

**Authors:** Flora De Conto

24 Jun 2026

Dear Dr. Kim,

Thank you for submitting your manuscript to PLOS ONE. After careful consideration, we feel that it has merit but does not fully meet PLOS ONE’s publication criteria as it currently stands. Therefore, we invite you to submit a revised version of the manuscript that addresses the points raised during the review process.

==============================

The submitted article addresses a relevant topic, but it presents some methodological shortcomings that need to be addressed. It is also important to highlight the innovative nature of the molecular diagnostic approach employed, although it has some limitations. I believe a thorough review based on the reviewers' comments is warranted.

==============================

Please submit your revised manuscript by Aug 08 2026 11:59PM. If you will need more time than this to complete your revisions, please reply to this message or contact the journal office at plosone@plos.org. When you're ready to submit your revision, log on to https://www.editorialmanager.com/pone/ and select the 'Submissions Needing Revision' folder to locate your manuscript file.

- A letter that responds to each point raised by the academic editor and reviewer(s). You should upload this letter as a separate file labeled 'Response to Reviewers'.
- A marked-up copy of your manuscript that highlights changes made to the original version. You should upload this as a separate file labeled 'Revised Manuscript with Track Changes'.
- An unmarked version of your revised paper without tracked changes. You should upload this as a separate file labeled 'Manuscript'.

If you would like to make changes to your financial disclosure, please include your updated statement in your cover letter. Guidelines for resubmitting your figure files are available below the reviewer comments at the end of this letter.

If applicable, we recommend that you deposit your laboratory protocols in protocols.io to enhance the reproducibility of your results. Protocols.io assigns your protocol its own identifier (DOI) so that it can be cited independently in the future. For instructions see: https://journals.plos.org/plosone/s/submission-guidelines#loc-laboratory-protocols. Additionally, PLOS ONE offers an option for publishing peer-reviewed Lab Protocol articles, which describe protocols hosted on protocols.io. Read more information on sharing protocols at https://plos.org/protocols?utm_medium=editorial-email&utm_source=authorletters&utm_campaign=protocols.

As the corresponding author, your ORCID iD is verified in the submission system and will appear in the published article. PLOS supports the use of ORCID, and we encourage all coauthors to register for an ORCID iD and use it as well. Please encourage your coauthors to verify their ORCID iD within the submission system before final acceptance, as unverified ORCID iDs will not appear in the published article. *Only*  the individual author can complete the verification step; PLOS staff *cannot*  verify ORCID iDs on behalf of authors.

We look forward to receiving your revised manuscript.

Kind regards,

Flora De Conto, Ph.D.

Academic Editor

PLOS One

Journal Requirements:

When submitting your revision, we need you to address these additional requirements.

1. Please ensure that your manuscript meets PLOS ONE’s style requirements, including those for file naming. The PLOS ONE style templates can be found at

https://journals.plos.org/plosone/s/file?id=wjVg/PLOSOne_formatting_sample_main_body.pdf and

https://journals.plos.org/plosone/s/file?id=ba62/PLOSOne_formatting_sample_title_authors_affiliations.pdf

2. Thank you for stating the following financial disclosure:

“This study was funded by Gyeongsang National University Changwon Hospital (GNUCHBRIF-2024-0005) and the Ministry of Trade, Industry, and Energy of Korea (RS-2024-00403563).”

Please state what role the funders took in the study.  If the funders had no role, please state: "The funders had no role in study design, data collection and analysis, decision to publish, or preparation of the manuscript."

If this statement is not correct you must amend it as needed.

Please include this amended Role of Funder statement in your cover letter; we will change the online submission form on your behalf.

3. Your ethics statement should only appear in the Methods section of your manuscript. If your ethics statement is written in any section besides the Methods, please move it to the Methods section and delete it from any other section. Please ensure that your ethics statement is included in your manuscript, as the ethics statement entered into the online submission form will not be published alongside your manuscript.

4.  We note that you have indicated that there are restrictions to data sharing for this study. PLOS only allows data to be available upon request if there are legal or ethical restrictions on sharing data publicly. For more information on unacceptable data access restrictions, please see http://journals.plos.org/plosone/s/data-availability#loc-unacceptable-data-access-restrictions.

Before we proceed with your manuscript, please address the following prompts:

a) If there are ethical or legal restrictions on sharing a de-identified data set, please explain them in detail (e.g., data contain potentially identifying or sensitive patient information, data are owned by a third-party organization, etc.) and who has imposed them (e.g., a Research Ethics Committee or Institutional Review Board, etc.). Please also provide contact information for a data access committee, ethics committee, or other institutional body to which data requests may be sent.

b) If there are no restrictions, please upload the minimal anonymized data set necessary to replicate your study findings to a stable, public repository and provide us with the relevant URLs, DOIs, or accession numbers. For a list of recommended repositories, please see

https://journals.plos.org/plosone/s/recommended-repositories. You also have the option of uploading the data as Supporting Information files, but we would recommend depositing data directly to a data repository if possible.

We will update your Data Availability statement on your behalf to reflect the information you provide

5. If the reviewer comments include a recommendation to cite specific previously published works, please review and evaluate these publications to determine whether they are relevant and should be cited. There is no requirement to cite these works unless the editor has indicated otherwise.

Additional Editor Comments :

The submitted article addresses a relevant topic. However, it requires further methodological clarification, as well as a better definition of the role of molecular diagnostics versus traditional diagnostics. The issues specifically highlighted by the reviewers must be addressed.

[Note: HTML markup is below. Please do not edit.]

Reviewers' comments:

Reviewer’s Responses to Questions

**Comments to the Author**

1. Is the manuscript technically sound, and do the data support the conclusions?

Reviewer #1: Yes

Reviewer #2: Yes

Reviewer #3: Yes

**********

2. Has the statistical analysis been performed appropriately and rigorously?

Reviewer #1: Yes

Reviewer #2: Yes

Reviewer #3: I Don't Know

**********

3. Have the authors made all data underlying the findings in their manuscript fully available?

The PLOS Data policy

Reviewer #1: Yes

Reviewer #2: No

Reviewer #3: Yes

**********

4. Is the manuscript presented in an intelligible fashion and written in standard English?

Reviewer #1: Yes

Reviewer #2: Yes

Reviewer #3: Yes

**********

Reviewer #1: I have carefully reviewed your study. By investigating the clinical performance and clinical impact of the BioFire FilmArray Pneumonia Panel in comparison with conventional culture, your study addresses a timely and critically important question for clinicians. In particular, the significant reduction in the time to antibiotic change and the decrease in hospital length of stay are notable findings. However, the methodological challenges and shortcomings I detail below present significant obstacles to the publication of the study in its current form. Therefore, I believe major revisions to the manuscript are necessary.

Antibiotic modification is defined quite broadly as "escalation, de-escalation, or any change in regimen." This definition makes it impossible to distinguish whether a clinician made a decision based on FilmArray findings or on concurrent biochemical, radiological, or other clinical data. Distinguishing modifications that can truly be attributed to FilmArray from this broad set requires a review of both the operational definition and the analytical method.

Although the authors are aware of this limitation, the fact that the detection of AMR genes was not validated by conventional susceptibility test results significantly weakens the clinical interpretation of resistance gene data. For example, it is unknown whether treatment was actually changed after KPC positivity was detected. This shortcoming significantly limits the study’s claims regarding its impact on antimicrobial stewardship.

It is not specified whether these resistance genes have been investigated in pathogens cultured in vitro. If they have not been investigated, the fact that this comparison was not made appears to be a shortcoming.

Reviewer #2: This manuscript presents a retrospective before-and-after study evaluating the diagnostic performance and clinical impact of the BioFire FilmArray Pneumonia Panel. While the topic is clinically relevant, the study suffers from significant methodological limitations—most critically, the inability to establish causality due to its uncontrolled design, substantial baseline imbalances between groups, and incomplete statistical reporting. These issues substantially undermine the conclusions drawn.

Methods 121–168; Results 245–291:The primary clinical impact analysis compares two sequential time periods (Jan–Nov 2022 vs. Dec 2022–Dec 2023) without a concurrent control group or randomization. This uncontrolled design allows for significant confounding by temporal changes in clinical practice, antibiotic stewardship, patient case-mix, seasonal pathogen prevalence, and institutional policy shifts.The authors should consider reframing all conclusions to reflect the observational nature of the study. Replace causal language (e.g., "FilmArray testing was associated with...") with cautious, descriptive language (e.g., "In this before-and-after comparison, the FilmArray group exhibited...").

Supplementary Table 3; Statistical Analysis 163–168: There are meaningful differences between the two groups that are not addressed: the FilmArray group is significantly younger, has higher CRP levels (suggesting higher illness severity), and differs in ICU proportion and residence distribution. Currently, the authors rely solely on univariate comparisons (t-tests and chi-square). The absence of multivariable analysis is a critical omission. Perform multivariable regression models (logistic or linear) or propensity score matching to adjust for these baseline imbalances (age, CRP, ICU status, residence). This is necessary to determine if the observed clinical benefits persist after adjustment.

Results 248–254: The manuscript reports a decrease in overall antibiotic days in the FilmArray group, yet paradoxically reports longer antibiotic exposure during the first seven days (5.41 vs. 4.62 days, p=0.012). This contradiction undermines the central narrative that rapid molecular testing enables earlier antibiotic optimization. Provide an explicit discussion and explanation for this paradox. The authors should consider analyzing this by severity subgroup or examining whether the difference is driven by specific pathogen profiles.

While CIs are mentioned in the abstract, the main results (Table 4) only report mean values and p-values. Report 95% CIs in all main results tables and consider adding effect sizes for continuous outcomes to improve the interpretability of the findings.

Please add and follow both the STARD and STROBE reporting guidelines, with the completed reporting checklists provided in the Supplementary Materials.

Reviewer #3: First I would like to thank the Editor and the Authors for the opportunity to review this paper.

This study explores performance characteristics as well as clinical utility and clinical impact of the Filmarray Biofire Pneumonia panel. The principal strength of this study is a clear focus on clinical impact of sophisticated, and fairly novel laboratory methodology. However, some added details and clarity on the study protocol and a more in depth exploration of the strengths and weaknesses of the diagnostic test are in my opinion warranted. With clarifications and a revision, I believe this article will be suitable for publication and provides valuable information to diagnostic laboratories and clinicians alike.

General comments/Major revision

1) The utility and performance characteristics of the tests viral targets are mentioned only in passing, and not sufficiently quantified or discussed. Without adding information on these, the article provides only a narrow and biased view of the tests clinical utility. For example how did a positive result on a viral target affect antibiotic de-escalation? And importantly; how well did Filmarray perform against reference tests for viral targets, and which tests were used as reference tests?

2) For the bacterial targets, the comparison of clinical impact between the two study periods was only done with sputum samples, if I understood correctly. The authors should either include culture results for BAL samples prior to the adoption of Filmarray assay as well, or explain why this information is not available.

3) The routine culture methods and bacterial identification methods for culture are not described in the article. For the reader to adequately estimate the performance characteristics of the multiplex PCR test, more information on the reference testing methods is required.

4) Please verify that the article fulfills the STARD 2015 items

Detailed comments and suggestions

ABSTRACT:

49-50 Please confirm whether the time to change is from test result (as is stated in the abstract and later in the text) or from the time from sample collection or time from sample arriving to laboratory.

50-51 The phrase describing escalation of sixty percent and de-escalation of thirty percent can seem unclear to the reader. Consider revising either adding detail and clarity, or simplifying to just stating there was no significant difference in the patterns of escalation or de-escalation, and provide the additional detail in the article.

MATERIALS AND METHODS:

127-128 A table or a figure demonstrating the concordance (or lack of concordance) between BAL and sputum results from individuals who had both sample materials available would be an interesting addition to the paper.

133-136 Consider adding more detail on the routine culture and related methods here, or possibly as a supplementary material. Referring to previous articles seems insufficient, especially as between the two references noted here, there are differences in the types of culture media used in standard of care testing, reference testing or comparator testing.

141 Are these the intended references? Their absolute relevance for the diagnostic performance analysis seems questionable, ie these publications are hardly the first to describe the use of sensitivity, specificity, PPV and NPV, in the way they are utilized in the current study. In addition, there are differences between these two references, (for example in their approach to discrepant analysis), which makes clearer explanation of the approaches selected for this current study even more essential. As far as I can deduce from the tables, the sensitivity and specificity are calculated based on worst case scenario (ie. Assuming the culture result is always more ”correct” than the Filmarray result, possibly underestimating the PCR tests performance?)? If so, this could be stated explicitly. Consider revising this section to provide the reader clarity on your rationale and methodology, and additionally verify that the references are correct.

147 ”According to the above period…” I suggest stating the specific time period, as it is laborious for the reader to go back to the beginning to check it.

149 ”Were categorized to the sputum culture group..” Meaning no BAL samples were included in the culture group? The lack of BAL samples in the ”pre-Biofire” group/comparator group should be addressed, see also major revision 2.

149 ”electric”, please correct to ”electronic”.

153-154 The Filmarray time period for the clinical impact comparison was defined as same as the diagnostic performance evaluation, but here is stated that only 200 samples were included in the Filmarray group. Please clarify in the article, what were the inclusion/exclusion criteria here?

158-159 Please confirm whether the time to antibiotic change is from test result, or from the time from sample collection or time from sample arriving to laboratory. If it is truly from reporting the test result, the mean times, especially for the sputum culture group, in the results section seem rather long and in that case would be beneficial to discuss, whether antibiotic changes this long after reporting the results are related to the laboratory results or are they attributable to other factors.

In Table 5 the total number of samples includes only the patients receiving antibiotic treatment. As this number is notably lower than the total number of samples in each group, it appears evident that many of the samples that were positive for bacterial pathogens in Filmarray testing were from patients that were not clinically considered true bacterial infections (only 81 from 200 patients in the Filmarray group were receiving antibiotics according to this table; In patients considered clinically as having bacterial pneumonia, antibiotic treatment would have undoubtedly been initiated). I highly recommend exploring this (patients positive for bacterial pathogens, but not receiving antibiotic treatment) in the results and discussion.

-The method section seems to lack clear information on additional testing that the patients with Biofire Filmarray testing results underwent. Later in the results section it is clear that most these patients underwent conventional culture as well, but this should be described in the materials and methods section as well. For example details on whether selective culture for Legionella species (or urine antigen testing for Legionella pneumophila) was performed on any subset of the patients, and so on for other pathogens, as this would help assess the performance of the multiplex test for different contexts.

-If available, results for blood cultures from the patients would also be of great interest, and clarify the possible additional value of the Biofire Pneumonia panel in pneumonia patients with positive blood cultures.

-Importantly, comparison to other detection methods for viral pathogens is lacking. Please describe what were the reference methods used in the laboratory to evaluate the performance of the viral targets of the Biofire Filmarray assay. See also major revision note 1.

-Please explain, how were deaths handled in the analysis of hospital stay length? Excluded from analysis?

-Please provide definitions for antibiotic escalation and antibiotic de-escalation that were used in the study.

RESULTS

Figures present only percentages, consider adding a table of the Filmarray findings that were missed by culture and vice versa.

Consider if Table 3 is necessary? And were all multiple findings considered significant ie.true polymicrobial infections? Any confirmatory testing performed, or additional evaluation of clinical presentation? Personally I would find it more interesting to explore the clinical significance of multiple findings, rather than the number of possible pathogens detected.

The comparison of the demographic details between the study groups is presented in Supplementary file 3. As this is important information consider transferring it to the main article.

Table 4: Please include in the table or in the text description what was the total number of patients on each row.

275 and Table 5: For antibiotic modification, were other factors considered, for example modifications based on clinical improvement/deterioration? Consider adding a flowchart to clarify escalation and de-escalation that different patient groups underwent. Ideally the flow chart would include information on the type of result that prompted the modification, or a categorical evaluation on whether the modification was truly warranted based on the result, and whether there had been results, that would have warranted escalation or de-escalation, but did not result in changes in patient management.

DISCUSSION

318-319 ”Consulting the list of bacteria for this group shows that most of them appear to be true pathogens”. Please clarify, what do the authors mean by this phrase?

319 ”In THE systematic review..”, I believe should be: ”in A systematic review”

327 Could the authors refer here to surveillance data from their region?

331 Consider discussing the possibility of colonization, thus leading to unnecessary antibiotic escalation?

337-339: Consider discussing also what are the advantages of culture in comparison with molecular methods? Such as semi-quantitative nature, scope of detectable bacteria (and fungi) more comprehensive than in a targeted panel, possibilities for selective cultures and phenotypic susceptibility testing?

356-357 ”several studies”, but only one reference, consider adding other relevant references or rephrasing.

362-364 seems to contradict with 374-376, so please specify how you define the difference between antibiotic optimization and antibiotic modification.

366 I believe it would be important to evaluate whether the Filmarray results even would consistently enough warrant de-escalation to have a detectable effect on antibiotic prescription patterns? Ie if the Filmarray result is, for example polymicrobial (as it often is), it seems natural that the clinician would await for culture results and/or clinical improvement, before de-escalation. The authors could explore already in the results section, what is the proportion of truly actionable results in a real life scenario.

410 ”Lack of reliability”, can the authors be more clear; does this refer to the clinicians lack of confidence in the results (if so, how was this evaluated or measured)? Or does it refer to the fact, that the absence of the genes included in the panel does not necessarily reflect phenotypic susceptibility?

414-415 Can this statement be made with confidence? Is it shown in the article, that pathogens responsible for the clinical disease or AMR genes were missed by culture, especially when correlation with culture based susceptibility testing or culture based AMR screening was not included in the study? Consider revising to better reflect the actual results of the current study.

-Because of the retrospective nature of the study, the differences observed between the timeframe before Filmarray use and during the Filmarray use may be due to various factors unrelated to the newly adopted methodology. It is mentioned briefly in the manuscript (principally in 354-355, 390-392), but I believe it warrants to be acknowledged as a significant limitation even more clearly.

Some inherent limitations of molecular methods, that I believe could be mentioned or discussed in relation to the results:

-Discussion of clinically insignificant findings due to high sensitivity of the assay, and the risks related to this.

-Scalability of the methodology at its current price point?

**********

what does this mean?). If published, this will include your full peer review and any attached files.

If you choose “no”, your identity will remain anonymous but your review may still be made public.

**Do you want your identity to be public for this peer review?**  For information about this choice, including consent withdrawal, please see our Privacy Policy

Reviewer #1: No

Reviewer #2: No

Reviewer #3: **Yes:** Maarit Johanna Ahava

**********

[NOTE: If reviewer comments were submitted as an attachment file, they will be attached to this email and accessible via the submission site. Please log into your account, locate the manuscript record, and check for the action link "View Attachments". If this link does not appear, there are no attachment files.]

To ensure your figures meet our technical requirements, please review our figure guidelines: https://journals.plos.org/plosone/s/figures

You may also use PLOS’s free figure tool, NAAS, to help you prepare publication quality figures: https://journals.plos.org/plosone/s/figures#loc-tools-for-figure-preparation.

NAAS will assess whether your figures meet our technical requirements by comparing each figure against our figure specifications.

# Author comment: Author response to Decision Letter 1

**DOI:** 10.1371/journal.pone.0358986.r002

5 Aug 2026

Response to Reviewers

Manuscript: Clinical Performance and Clinical Impact of the BioFire FilmArray Pneumonia Panel Compared with Conventional Culture in Hospitalized Patients with Pneumonia

PLOS ONE — Major Revision

General Response

We thank the Academic Editor and the three reviewers for their careful evaluation of our manuscript and for their constructive comments. Their feedback prompted us to conduct a comprehensive re-audit of the analytic dataset underlying the clinical-impact comparison. This re-examination identified several inconsistencies in the derivation of clinical outcome variables between the two study periods. We harmonized the operational definitions, recalculated all clinical-impact outcomes using identical criteria in both groups, and repeated the analyses using multivariable adjustment together with propensity score–based sensitivity analyses. These revisions have strengthened the methodological rigor and transparency of our study.

Two reviewer comments prompted particularly extensive revisions.

• Reviewer #2 noted that our clinical-impact analysis relied entirely on unadjusted univariate comparisons despite meaningful baseline imbalance and asked whether the observed findings remained after appropriate statistical adjustment.

• Reviewer #3 asked us to clarify whether time to antibiotic change had been measured from reporting of the microbiological result or from specimen collection, and pointed out that the reported value for the sputum culture group appeared implausibly long if it had indeed been measured from result reporting.

Reviewer #3 correctly identified an inconsistency in our original analysis. Re-examination showed that this outcome had been calculated from specimen collection in the sputum culture group but from result reporting in the FilmArray group. Because conventional sputum culture at our institution has a median turnaround time of approximately three days, whereas the FilmArray panel reports on the same day (median, 0 days), this inconsistency substantially exaggerated the apparent advantage of the panel. The audit also identified an inconsistent derivation of total antibiotic days between the two groups, together with a single implausible body mass index value (658.8 kg/m²) that distorted the mean and standard error reported in the baseline table.

Using the same 494-patient cohort (sputum culture group, n = 294; FilmArray group, n = 200), we therefore re-analyzed every clinical-impact outcome by applying a single pre-specified operational definition identically to both groups and incorporating the multivariable adjustment and propensity score–based sensitivity analyses requested by Reviewer #2. Accordingly, the baseline table, Tables 4 and 5, the Results, the Abstract, and the Conclusions have all been revised.

The revised analyses resulted in an important revision of one of our original conclusions.

Total antibiotic exposure was not reduced in the FilmArray group; instead, it was modestly but significantly increased (adjusted difference, +1.86 days; 95% CI, +0.29 to +3.44; P = 0.021). At the same time, implementation of the panel:

• increased the proportion of patients in whom the microbiological result led to an antibiotic decision, from 26.5% to 40.5% (adjusted OR, 1.86; 95% CI, 1.24–2.79; P = 0.003);

• shortened the interval between result reporting and that decision (adjusted difference, −0.59 days; 95% CI, −1.07 to −0.10; P = 0.019); and

• changed the nature of those decisions—from post hoc modification of already initiated empirical therapy (87.2% of actions in the culture group) to result-guided initiation of therapy in antibiotic-naïve patients (67.9% of actions in the FilmArray group; P < 0.001).

De-escalation, however, was no more frequent (30.8% vs. 35.3%; P = 0.81).

Importantly, these revised analyses resolve the apparent paradox identified by Reviewer #2. The inconsistency arose from differences in outcome derivation rather than from conflicting clinical findings. Once total antibiotic days were calculated identically in both groups, antibiotic exposure was consistently higher in the FilmArray group during both days 1–7 and the overall hospitalization. Both observations are explained by the same underlying pattern: the panel was used predominantly to initiate or broaden antibiotic therapy rather than to facilitate de-escalation. These findings are consistent with previously published randomized and observational studies (Virk et al., Lancet Microbe, 2024; Enne et al., Intensive Care Medicine, 2025; Chen et al., 2024; Plattner et al., 2024; Manda et al., 2025), which likewise reported that syndromic molecular panels more readily support escalation than de-escalation of antimicrobial therapy.

Accordingly, the Discussion and Conclusions were revised to emphasize that the BioFire FilmArray Pneumonia Panel should be interpreted as a complementary diagnostic tool to support antimicrobial stewardship, rather than as a stand-alone strategy for reducing antibiotic exposure.

We recognize that corrections of this magnitude require complete transparency. We have therefore deposited the fully de-identified analytic dataset together with the complete analysis script in a public repository, enabling independent verification of every value reported in the revised tables.

________________________________________

Academic Editor

Comment

"The submitted article addresses a relevant topic. However, it requires further methodological clarification, as well as a better definition of the role of molecular diagnostics versus traditional diagnostics."

Response: We appreciate the Academic Editor’s thoughtful comments.

We have completely rewritten the Statistical Analysis section to improve methodological clarity. Detailed responses are provided under Reviewer #2 (Comments 2 and 4) and Reviewer #3 (comments regarding lines 141 and 153–158). We also revised the Discussion and Conclusions to more clearly distinguish the diagnostic role of the FilmArray Pneumonia Panel from its real-world clinical impact on antimicrobial prescribing.

Completed STROBE (clinical-impact analysis) and STARD 2015 (diagnostic-performance analysis) reporting checklists, together with a participant flow diagram, have been included as Supporting Information.

In addition, the Discussion has been revised to provide a clearer distinction between the respective roles of the BioFire FilmArray Pneumonia Panel and conventional culture. Specifically, we emphasize that the FilmArray panel facilitates earlier, result-guided initiation of antimicrobial therapy, but did not reduce overall antibiotic exposure or increase de-escalation compared with conventional culture in the present study. We also discuss the respective strengths and limitations of the two diagnostic methods.

Moreover, we changed the title from "Clinical Performance and Clinical Impact..." to "Diagnostic Performance and Clinical Impact..." because the word ‘clinical’ appeared twice in the original title. Importantly, the term "sputum culture group" was misleading because it included both sputum and bronchoalveolar lavage (BAL) specimens. Therefore, throughout the revised manuscript, this term has been replaced with "pre-implementation period." Accordingly, "FilmArray group" has been renamed "FilmArray period" to maintain consistent terminology.

Data availability

“We note that you have indicated that there are restrictions to data sharing for this study. PLOS only allows data to be available upon request if there are legal or ethical restrictions on sharing data publicly.”

Response: The fully de-identified minimal dataset needed to reproduce every value in Tables 1, 4 and 5, together with the complete analysis script, has been deposited in a public repository and the Data Availability Statement has been updated accordingly. The fully de-identified dataset together with the complete statistical analysis script has been deposited in Zenodo (DOI: https://doi.org/10.5281/zenodo.21767056).

Acknowledgements

We have revised the Acknowledgements section to include the following statement:

"We thank Myeongjin Seo, MT, for microbiological data curation, and Seung-Hee Shin, Health Information Management Specialist, for assistance with medical record review and patient identification."

This revision appropriately acknowledges their contributions to the study.

Reviewer #1

Comment 1 — Operational definition of antibiotic modification

"Antibiotic modification is defined quite broadly as 'escalation, de-escalation, or any change in regimen.' This definition makes it impossible to distinguish whether a clinician made a decision based on FilmArray findings or on concurrent biochemical, radiological, or other clinical data. Distinguishing modifications that can truly be attributed to FilmArray from this broad set requires a review of both the operational definition and the analytical method."

Response: We agree, and have revised both the operational definition and the analytical method.

The primary clinical-impact endpoint is now a result-driven antibiotic action, defined a priori as the initiation of a new antibiotic, or a change to an existing regimen, occurring on the day of, or within five days after, the final microbiological result was reported. Antibiotic actions preceding the report were not counted. A "one day before the report" window that had been permitted for the FilmArray group in the original analysis has been removed, so that the temporal rule is now identical in both groups. The definition, and the number of patients meeting it in each group, are stated in the Methods and footnoted in Tables 4 and 5.

We also now report what kind of action was taken (Table 5): initiation of therapy in an antibiotic-naive patient versus modification of an ongoing regimen, and, among modifications, escalation versus de-escalation. This distinction proved to be the single most informative finding of the study.

We nevertheless accept the reviewer’s deeper point: a temporal rule, however strict, cannot establish that the clinician acted because of the panel result rather than because of concurrent clinical, biochemical or radiological information. Our electronic records did not contain a structured field recording the clinician’s stated reason for each antibiotic decision, so a formal attribution analysis was not possible. We have removed all language implying attribution, and have added the following to the Limitations:

"Although the FilmArray Pneumonia Panel provides rapid microbiological information, antibiotic prescribing decisions are influenced by multiple clinical factors in addition to microbiological test results. Clinical improvement or deterioration, radiologic findings, inflammatory markers, and other patient-specific considerations may all contribute to treatment modification. Therefore, although antibiotic actions were evaluated only when they occurred within five days of result reporting, this temporal association should not be interpreted as establishing a causal relationship between FilmArray implementation and changes in antibiotic prescribing.."

Comments 2 and 3 — Validation of AMR genes against phenotypic susceptibility testing

“the detection of AMR genes was not validated by conventional susceptibility test results significantly weakens the clinical interpretation of resistance gene data. For example, it is unknown whether treatment was actually changed after KPC positivity was detected. This shortcoming significantly limits the study’s claims regarding its impact on antimicrobial stewardship. It is not specified whether these resistance genes have been investigated in pathogens cultured in vitro. If they have not been investigated, the fact that this comparison was not made appears to be a shortcoming.”

Response: We agree that validation of antimicrobial resistance (AMR) genes against phenotypic antimicrobial susceptibility testing (AST) would provide additional information regarding the clinical significance of the detected resistance genes. However, this analysis was beyond the scope of the present study. The primary objectives of this study were to evaluate the diagnostic performance of the BioFire FilmArray Pneumonia Panel compared with conventional culture and to assess its clinical impact on antibiotic management. Accordingly, we did not perform a systematic correlation analysis between AMR gene detection and phenotypic AST results. This limitation has been acknowledged in the revised manuscript, and we suggest that future studies specifically designed to investigate the concordance between molecular resistance determinants and phenotypic susceptibility are warranted.

Reviewer #2

Comment 1 — Uncontrolled before-and-after design; causal language

"...The authors should consider reframing all conclusions to reflect the observational nature of the study. Replace causal language (e.g., 'FilmArray testing was associated with...') with cautious, descriptive language (e.g., 'In this before-and-after comparison, the FilmArray group exhibited...')."

Response: We fully agree with the reviewer’s valuable comment and have revised the manuscript accordingly. Throughout the manuscript, statements implying a causal effect of the FilmArray Pneumonia Panel have been replaced with descriptive language appropriate for an observational before-and-after study. For example, expressions suggesting that the panel "led to" or "resulted in" changes in antibiotic management have been revised to describe observed differences between the two study periods without implying causality.

The Abstract Conclusions, Discussion, and Conclusions have all been rewritten to explicitly acknowledge that the study design does not permit causal inference. In addition, we have expanded the Limitations section to discuss potential temporal confounding, including secular changes in clinical practice, antimicrobial stewardship activities, patient case-mix, seasonal variation in pathogen prevalence, and institutional policy changes that may have influenced the observed findings.

________________________________________

Comment 2 — Baseline imbalance and the absence of multivariable analysis

"...the FilmArray group is significantly younger, has higher CRP levels..., and differs in ICU proportion and residence distribution. Currently, the authors rely solely on univariate comparisons (t-tests and chi-square). The absence of multivariable analysis is a critical omission. Perform multivariable regression models (logistic or linear) or propensity score matching to adjust for these baseline imbalances (age, CRP, ICU status, residence)."

Response: We appreciate this important comment, which prompted a comprehensive re-analysis of all clinical-impact outcomes and substantially strengthened the statistical analyses presented in the revised manuscript.

All clinical-impact outcomes are now reported as adjusted estimates. Multivariable models included the four variables identified by the reviewer (age, CRP, ICU admission, and residence type), together with sex and ECOG performance status. Continuous outcomes were analyzed using multivariable linear regression with heteroscedasticity-consistent (HC3) standard errors, whereas binary outcomes were analyzed using multivariable logistic regression.

Because IPTW was performed as a sensitivity analysis to assess the robustness of the primary multivariable analyses, the detailed IPTW results were not included in the manuscript.

Outcome Multivariable regression IPTW (propensity score)

Result-driven antibiotic action OR 1.86 (1.24–2.79), P = 0.003 OR 1.80 (1.20–2.69), P = 0.004

Time to antibiotic action, days −0.59 (−1.07 to −0.10), P = 0.019 −0.57 (−1.01 to −0.13), P = 0.011

Total antibiotic days +1.86 (+0.29 to +3.44), P = 0.021 +1.66 (+0.00 to +3.32), P = 0.050

Length of hospital stay, days −1.61 (−3.07 to −0.15), P = 0.031 −1.77 (−3.32 to −0.22), P = 0.025

30-day mortality OR 1.50 (0.90–2.48), P = 0.118 OR 1.46 (0.92–2.32), P = 0.105

During the re-analysis, we identified an additional technical issue, which we report here for transparency. The residence variable originally included a nursing-home category containing only two patients in the entire cohort (one in each study group). Retaining this category as an independent model term produced a leverage value close to 1 and unstable heteroscedasticity-consistent standard errors. We therefore combined this category with the clinically adjacent long-term-care category, resulting in a three-level residence variable (home, long-term care, and acute-care hospital). This modification is described in the Statistical Analysis section and produced more stable model estimates and statistically reliable confidence intervals.

The reviewer specifically asked whether the observed findings remained after adjustment. The revised analyses provide a nuanced answer. The association between the FilmArray implementation period and result-driven antibiotic decisions as well as earlier antibiotic modification remained statistically significant after adjustment. In contrast, the apparent reduction in total antibiotic exposure observed in the original analysis was no longer supported; instead, adjusted analyses showed a modest but statistically significant increase in total antibiotic days. Likewise, the reduction in hospital length of stay persisted after adjustment but was considerably smaller than originally reported (adjusted reduction, 1.6 days versus the unadjusted estimate 5.0 days; P = 0.031 by multivariable regression, P = 0.025 by IPTW, and P = 0.038 in the survivor-only analysis).

________________________________________

Comment 3 — The day 1–7 paradox

"The manuscript reports a decrease in overall antibiotic days in the FilmArray group, yet paradoxically reports longer antibiotic exposure during the first seven days (5.41 vs. 4.62 days, p = 0.012). This contradiction undermines the central narrative. Please provide an explicit discussion and explanation for this paradox."

Response: We appreciate this insightful observation. The apparent paradox has now been resolved through the revised analyses.

The contradiction resulted from an inconsistency in the derivation of total antibiotic days, as described in the General Response. After recalculating antibiotic exposure using identical operational definitions in both study groups, the apparent contradiction disappeared. Antibiotic exposure was consistently higher in the FilmArray group during both the first seven days and the entire hospitalization.

The revised analyses also provide a plausible explanation for these findings. As shown in the Table 5, 67.9% of result-driven antibiotic actions in the FilmArray group represented initiation of therapy in previously antibiotic-naïve patients, compared with only 12.8% in the conventional culture group (P < 0.001). In contrast, de-escalation occurred at similar frequencies in both groups (30.8% vs. 35.3%; P = 0.81).

Taken together, these findings indicate that the FilmArray panel facilitated earlier and more microbiologically informed antibiotic decisions, but it did not shorten overall antibiotic exposure because clinicians more often used the results to initiate or broaden antimicrobial therapy rather than to de-escalate treatment. We have added a dedicated paragraph to the Discussion to explain this finding and revised the Conclusions accordingly.

________________________________________

Comment 4 — Confidence intervals and effect sizes

“While CIs are mentioned in the abstract, the main results (Table 4) only report mean values and p-values. Report 95% CIs in all main results tables and consider adding effect sizes for continuous outcomes to improve the interpretability of the findings.”

Response: Thank you for this helpful suggestion. The manuscript has been revised accordingly. Every outcome reported in the revised Table 4 now includes the number of patients contributing to the analysis, mean ± standard deviation, median (interquartile range), and the adjusted effect estimate (mean difference or odds ratio) with its corresponding 95% confidence interval.

The Statistical Analysis section has been revised to clarify the statistical methods used. Continuous outcomes were assessed for normality using the Shapiro–Wilk test. Because the primary analyses were based on adjusted multivariable regression models, only adjusted effect estimates with corresponding 95% confidence intervals are presented in the revised Table 5.________________________________________

Comment 5 — STARD and STROBE

Response: We appreciate the reviewer’s suggestion. Completed STROBE (for the clinical-impact analysis) and STARD 2015 (for the diagnostic-performance analysis) checklists have been included as Supporting Information, together with a STROBE participant flow diagram.

To comply with the STARD 2015 reporting guidelines, we added three sentences to the Methods section regarding (1) the reference standard, (2) blinding, and (3) sample size.

1) Line 160. Because this was a retrospective observational study, all eligible patients identified during the predefined study period were included, and no formal sample size calculation was performed. FilmArray testing and conventional microbiological culture were performed independently as part of routine clinical practice.

2) Line 161. FilmArray testing and conventional microbiological culture were performed independently as part of routine clinical practice, and no additional interpretation of either test was performed with knowledge of the corresponding results from the other method.

3) Line 131. Because of the retrospective study design, no formal sample size calculation was performed; instead, the sample size was determined by the number of eligible patients identified during the predefined study period.

Reviewer #3

1) “The utility and performance characteristics of the tests viral targets are mentioned only in passing, and not sufficiently quantified or discussed. Without adding information on these, the article provides only a narrow and biased view of the tests clinical utility. For example how did a positive result on a viral target affect antibiotic de-escalation? And importantly; how well did Filmarray perform against reference tests for viral targets, and which tests were used as reference tests?”

Response: We appreciate the reviewer’s thoughtful comment. We agree that evaluation of the viral targets would require comparison with an appropriate reference method and assessment of their potential impact on clinical decision-making.

However, the present study was designed to evaluate the diagnostic performance of the BioFire FilmArray Pneumonia Panel for bacterial pathogens and its clinical impact on antibiotic management. Routine laboratory testing for respiratory viruses was not generally performed during the study period; therefore, no independent reference standard was available for assessing the diagnostic performance of the viral targets.

Likewise, the contribution of viral target detection to antibiotic de-escalation could not be reliably evaluated because antibiotic prescribing decisions were based on the overall clinical assessment, including patients' clinical presentation, radiologic findings, and microbiological results, rather than viral detection alone.

Accordingly, diagnostic performance analyses were intentionally restricted to bacterial pathogens, and evaluation of the viral targets and their influence on antibiotic management was beyond the scope of the present study. We have clarified this point in the revised Methods, Results, and Discussion.

2) “For the bacterial targets, the comparison of clinical impact between the two study periods was only done with sputum samples, if I understood correctly. The authors should either include culture results for BAL samples prior to the adoption of Filmarray assay as well, or explain why this information is not available.

Response: We appreciate the reviewer’s important comment. This concern arose from our original use of the term "sputum culture group," which was misleading. The clinical-impact analysis was not restricted to sputum specimens. Both sputum and bronchoalveolar lavage (BAL) specimens were included in the pre-implementation period and were evaluated using conventional culture, while the post-implementation period included the same specimen types evaluated using the FilmArray Pneumonia Panel in addition to conventional culture.”

To avoid this misunderstanding, we have revised the terminology throughout the manuscript. The comparator group is now consistently referred to as the pre-implementation period, rather than the sputum culture group, and we have clarified in the Methods that both sputum and BAL specimens were included in the clinical-impact analysis.

3) “The routine culture methods and bacterial identification methods for culture are not described in the article. For the reader to adequately estimate the performance characteristics of the multiplex PCR test, more information on the reference testing methods is required.”

Response: We agree that a more detailed description of the conventional microbiological methods is necessary to allow readers to appropriately interpret the diagnostic performance of the FilmArray Pneumonia Panel.

Accordingly, we have expanded the Methods section to describe the routine culture procedures used for respiratory specimens, including specimen processing, bacterial culture methods, organism identification, and antimicrobial susceptibility testing. We believe that these additional methodological details improve the transparency and reproducibility of the study.

4) “Please verify that the article fulfills the STARD 2015 items.”

Response: Completed STROBE (for the clinical-impact analysis) and STARD 2015 (for the diagnostic-performance analysis) checklists have been included as Supporting Information, together with a STROBE participant flow diagram. No substantive changes to the manuscript text were required because the reporting checklists are provided as supplementary files.

Abstract, lines 49–50 — Reference point for time to antibiotic change

"Please confirm whether the time to change is from test result (as is stated in the abstract and later in the text) or from the time from sample collection or time from sample arriving to laboratory."

Response We thank the reviewer for this careful observation. The reviewer correctly identified an inconsistency in our original analysis, and this question led us to uncover a genuine error.

The reported data of 3.83 days for the sputum culture group had in fact been measured from specimen collection, whereas the corresponding FilmArray value had been measured from result reporting. Because the median turnaround time of sputum culture at our institution is 3 days, compared with 0 days for the FilmArray Pneumonia Panel, this inconsistency accounted for most of the apparent difference between the two groups.

Both groups are now measured from the same reference point—the reporting of the final microbiological result. Using this consistent definition, the mean time to a result-driven antibiotic action was 1.03 days in the sputum culture group and 0.47 days in the FilmArray group (adjusted difference, −0.59 days, 95% CI −1.07 to −0.10; P = 0.019). A statistically significant difference therefore remains, although the magnitude of the effect is substantially smaller than originally reported. The Abstract, Results, Table 5, and Conclusions have all been revised accordingly.

Abstract, lines 50–51 — Escalation / de-escalation wording

50-51 “The phrase describing escalation of sixty percent and de-escalation of thirty percent can seem unclear to the reader. Consider revising either adding detail and clarity, or simplifying to just stating there was no significant difference in the patterns of escalation or de-escalation, and provide the additional detail in the article.”

Response: We adopted the reviewer’s second suggestion. The Abstract now states that, among patients whose ongoing antibiotic regimen was modified, the proportions of escalation and de-escalation did not differ significantly between the two study periods, with the complete distribution of antibiotic actions presented in Table 5.

Definitions of escalation and de-escalation

"Please provide definitions for antibiotic escalation and antibiotic de-escalation that were used in the study."

Response: These definitions have now been added to the Methods. Escalation was defined as a change to an antimicrobial agent or combination with a broader antibacterial spectrum than the regimen currently in use, including the addition of a new agent. De-escalation was defined as a change to a narrower-spectrum regimen or discontinuation of one or more agents from combination therapy without broadening antibacterial coverage.

Initiation of antibiotic therapy in patients who were not receiving antimicrobial treatment at the time of microbiological result reporting is now reported separately in Table 5 as "result-guided initiation", rather than being classified as escalation, because no existing antibiotic regimen was available for comparison

127-128 “A table or a figure demonstrating the concordance (or lack of concordance) between BAL and sputum results from individuals who had both sample materials available would be an interesting addition to the paper.”

Response: We appreciate the reviewer’s thoughtful suggestion. We agree that a comparison of the concordance between bronchoalveolar lavage (BAL) and sputum results in patients who had both specimen types available would be of interest and could provide additional insight into the diagnostic performance of the FilmArray Pneumonia Panel. However, this analysis was beyond the scope of the present study, which was designed to compare the overall diagnostic performance of the FilmArray Pneumonia Panel with conventional culture and to evaluate its clinical impact on antibiotic management. Therefore, we did not perform this additional analysis. We agree that such an analysis would be valuable and consider it an important topic for future investigation.

141 “Are these the intended references? Their absolute relevance for the diagnostic performance analysis seems questionable, ie these publications are hardly the first to describe the use of sensitivity, specificity, PPV and NPV, in the way they are utilized in the current study. In addition, there are differences between these two references, (for example in their approach to discrepant analysis), which makes clearer explanation of the approaches selected for this current study even more essential. As far as I can deduce from the tables, the sensitivity and specificity are calculated based on worst case scenario (ie. Assuming the culture result is always more ”correct” than the Filmarray result, possibly underestimating the PCR tests performance?)? If so, this could be stated explicitly. Consider revising this section to provide the reader clarity on your rationale and methodology, and additionally verify that the references are correct.”

Response: We appreciate this insightful comment and agree that the rationale for our analytical approach, together with the methods used to calculate the diagnostic performance measures, should be described more explicitly.

In the revised manuscript, we have clarified that diagnostic performance was evaluated using conventional culture as the reference standard, and that sensitivity, specificity, positive predictive value (PPV), and negative predictive value (NPV) were calculated based on standard 2 × 2 contingency tables. We did not perform discrepant analysis or any adjudication of discordant results. Consequently, FilmArray-positive/culture-negative results were classified as false-positive results and FilmArray-negative/culture-positive results as false-negative results for the purpose of calculating diagnostic performance. As the reviewer correctly noted, this represents a conservative ("worst-case") approach that may underestimate the diagnostic performance of the molecular assay.

Because the statistical methods used are standard and self-explanatory, we removed the previous references and instead expanded the methodological description to explicitly explain how diagnostic performance was calculated.

147 ”According to the above period.. I suggest stating the specific time period, as it is laborious for the reader to go back to the beginning to check it.”

Response: We appreciate the reviewer’s helpful suggestion. The manuscript has been revised to state the specific study period directly, rather than referring to "the above period," thereby improving clarity and readability.

149 ”Were categorized to the sputum culture group..” Meaning no BAL samples were included in the culture group? The lack of BAL samples in the ”pre-Biofire” group/comparator group should be addressed, see also major revision 2.”

Response: We appreciate the reviewer’s careful and very important observation. We agree that the original wording, "sputum culture group," was misleading and could be interpreted as indicating that only sputum specimens were included in the comparator group. This was not the case. The pre-implementation period included both sputum and bronchoalveolar lavage (BAL) specimens, all of which were evaluated by conventional culture.

To avoid this misunderstanding, we have revised the terminology throughout the manuscript. The comparator group is now consistently referred to as the pre-implementation period, rather than the sputum culture group. Similarly, the "FilmArray group" has been renamed the "FilmArray period" to maintain consistent terminology. We have also clarified that both sputum and BAL specimens were included during the pre-implementation period, whereas the post-implementation period included the same specimen types evaluated using the FilmArray Pneumonia Panel in addition to conventional culture.

________________________________________

Methods, lines 153–154 — Inclusion and exclusion criteria

153-154 “The Filmarray time period for the clinical impact comparison was defined as same as the diagnostic performance evaluation, but here is stated that only 200 samples were included in the Filmarray group. Please clarify in the article, what were the inclusion/exclusion criteria here?”

Response: We apologize that these eligibility criteria were not clearly described in the original submission. They are now stated explicitly in the Methods, and a STROBE participant flow diagram has been added as Supporting Information to illustrate the number of patients excluded at each step.

Both groups comprised hospitalized patients with pneumonia who (i) received antibiotic therapy during admission and (ii) had a positive microbiological result—a bacterial pathogen detected by sputum culture or a bacterial target detected by the FilmArray panel. Patients were excluded if they had not received antibiotics, had a negative microbiological result, or—during the FilmArray period—had only atypical bacteria or viral targets detected.

The rationale for the final exclusion criterion has now been clarified in the manuscript. Conventional sputum culture cannot detect respiratory viruses; therefore, an equivalent "virus-only" category does not exist in the comparator group. Including such patients only in the FilmArray group would have introduced a population with, by definition, no microbiological indication for antibacterial therapy into one study arm but not the other, thereby biasing all antibiotic-related comparisons. Excluding these patients ensured that the two cohorts were comparable with respect to the microbiological indication for antibiotic treatment. These criteria yielded 294 patients during the pre-implementation period and 200 patients during the FilmArray period.

We have also clarified at the beginning of both Results subsections that the diagnostic accuracy analysis (558 specimens) and the clinical-impact analysis (494 patients) were conducted in different study populations.

Methods,

158-159 “Please confirm whether the time to antibiotic change is from test result, or from the time from sample collection or time from sample arriving to laboratory. If it is truly from reporting the test result, the mean times, especially for the sputum culture group, in the results section seem rather long and in that case would be beneficial to discuss, whether antibiotic changes this long after reporting the results are related to the laboratory results or are they attributable to other factors.”

Response: The reviewer correctly inferred that the reported interval had not been measured from result reporting (see our response above).

Using a consistent definition based on result reporting, the mean interval to a result-driven antibiotic action was 1.03 days in the pre-implementation period and 0.47 days in the FilmArray period, while the median was 0 days in both groups. The implausibly long intervals that prompted the reviewer’s question have therefore been eliminated.

Furthermore, antibiotic actions occurring more than five days after result reporting are no longer considered result-driven, precisely because such decisions are increasingly likely to reflect subsequent clinical developments rather than the microbiological result itself. This rationale is now explicitly described in the Methods and further discussed in the Limitations.

“In Table 5 the total number of samples includes only the patients receiving antibiotic treatment. As this number is notably lower than the total number of samples in each group, it appears evident that many of the samples that were positive for bacterial pathogens in Filmarray testing were from patients that were not clinically considered true bacterial infections (only 81 from 200 patients in the Filmarray group were receiving antibiotics according to this table; In patients considered clinically as having bacterial pneumonia, antibiotic treatment would have undoubtedly been initiated). I highly recommend exploring this (patients positive for bacterial pathogens, but not receiving antibiotic treatment) in the results and discussion.”

Response: We appreciate the reviewer’s careful observation. The apparent discrepancy results from a misunderstanding of the denominator used in Table 5. Table 5 does not include all patients in the clinical-impact cohort or all patients who received antibiotic therapy. Rather, it includes only patients whose antibiotic regimen was modified after the microbiological result became available. Patients whose antibiotic treatment remained unchanged are not included in this table.

Therefore, the number of patients shown in Table 5 should not be interpreted as the number of patients who received antibiotic treatment or as evidence that bacterial pathogen-positive patients were not treated with antibiotics. To avoid this misunderstanding, we have revised the table title and footnotes to clarify that Table 5 summarizes only patients who underwent a microbiology-guided antibiotic modification.

“The method section seems to lack clear information on additional testing that the patients with Biofire Filmarray testing results underwent. Later in the results section it is clear that most these patients underwent conventional culture as well, but this should be described in the materials and methods section as well. For example details on whether selective culture for Legionella species (or urine antigen testing for Legionella pneumophila) was performed on any subset of the patients, and so on for other pathogens, as this would help assess the performance of the multiplex test for different contexts.”

Response: We agree that the additional microbiological testing performed during the FilmArray period should be described more clearly in the Methods.

In the revised manuscript, we have clarified that patients in the FilmArray period also underwent conventional microbiological culture as part of routine clinical care, and that the diagnostic performance analysis was based on the comparison between FilmArray results and conventional culture. We have also clarified that additional pathogen-specific investigations, such as selective culture for Legionella species or urinary antigen testing for Legionella pneumophila, were not performed systematically according to the study protocol and therefore were not incorporated into the diagnostic performance analysis. Consequently, these tests were not used as reference standards for evaluating the performance of the FilmArray Pneumonia Panel.

These clarifications have been added to the Methods section to improve the transparency of the study design and microbiological evaluation.

“If available, results for blood cultures from the patients would also be of great interest, and clarify the possible additional value of the Biofire Pneumonia panel in pneumonia patients with positive blood cultures.”

Response: We agree that blood culture results could provide additional information regarding the potential complementary value of the FilmArray Pneumonia Panel in patients with bacteremic pneumonia. However, the present study was designed to evaluate the diagnostic performance of the FilmArray Pneumonia Panel using respiratory specimens and to assess its clinical impact on antibiotic management. Accordingly, blood culture results were beyond the scope of the current study and were not included in the study analyses. We agree that integrating respiratory and blood culture findings would be of considerable interest and represents an important direction for future research.

“Importantly, comparison to other detection methods for viral pathogens is lacking. Please describe what were the reference methods used in the laboratory to evaluate the performance of the viral targets of the Biofire Filmarray assay. See also major revision note 1.”

Response: We agree that evaluation of the viral targets would require comparison with an appropriate reference method, such as a validated respiratory viral PCR assay. However, routine laboratory testing for respiratory viruses was not generally performed in the study population, and therefore no independent reference method was available for evaluating the viral targets detected by the FilmArray Pneumonia Panel.

Table 4 — Number of patients in each row

"Table 4: Please include in the table or in the text description what was the total number of patients on each row."

Response: Thank you for this helpful suggestion. The manuscript has been revised accordingly. Every row of the revised Table 5 states its analysis population and the number of patients contributing to it in each group. The footnotes also state how many patients were removed by each pre-specified truncation (antibiotic courses > 45 days: 14 in the culture group and 15 in the FilmArray group; hospital stays > 30 days: 94 and 43 respectively), and we now report a sensitivity analysis in which the 45-day truncation is removed. The conclusion is unchanged (adjusted ratio of means 1.37, 95% CI 1.16–1.62; P < 0.001, log-transformed model).

Handling of deaths in the length-of-stay analysis

"Please explain, how were deaths handled in the analysis of hospital stay length? Excluded from analysis?"

Response: Patients who died in hospital were included; their length of stay was the interval from admission to death. This is now stated explicitly in the Methods. Because in-hospital death shortens length of stay, and because 30-day mortality differed numerically between the groups, we repeated the analysis restricted to survivors as a sensitivity analysis. The estimate was essentially unchanged (adjusted difference −1.62 days, 95% CI −3.16 to −0.09; P = 0.038, versus −1.61 days, 95% CI −3.07 to −0.15; P = 0.031 in the primary analysis). We also now report 30-day mortality, which was omitted from the original Table 4: 19.8% in the sputum culture group and 23.1% in the FilmArray group (adjusted OR 1.50, 95% CI 0.90–2.48; P = 0.118).

Results:

Figures

“Figures present only percentages, consider adding a table of the Filmarray findings that were missed by culture and vice versa.”

Response: We thank the reviewer for this valuable suggestion. In response, we removed the previous figure showing concordance percentages between conventional culture and the FilmArray Pneumonia Panel. It has been replaced by two new tables (Tables 2A and 2B) presenting organism-level discordant results, including pathogens detected only by the FilmArray panel (culture-negative/FilmArray-positive) and those detected only by conventional culture (culture-positive/FilmArray-negative), separately for BAL and sputum specimens.

These tables provide substantially more clinically relevant information regarding discordant findings between the two diagnostic methods. The Results section has been revised accordingly, and the previous Figure 1 has been removed.

Tables.

“Consider if Table 3 is necessary? And were all multiple findings considered significant ie.true polymicrobial infections? Any confirmatory testing performed, or additional evaluation of clinical presentation? Personally I would find it more interesting to explore the clinical significance of multiple findings, rather than the number of possible pathogens detected.”

Response: We agree that the clinical significance of multiple pathogen detections is more important than simply reporting the number of organisms detected. Accordingly, Table 3 has been removed from the revised manuscript.

In the present retrospective study, we did not consider all multiple detections to represent true polymicrobial infections. No confirmatory microbiological testing or standardized clinical adjudication was performed to determine the clinical significance of each detected organism. Therefore, distinguishing true infection from colonization or clinically insignificant findings was beyond the scope of this study.

We have acknowledged this limitation in the Discussion and stated that future prospective studies integrating microbiological, radiological, and clinical data are warranted to better evaluate the clinical significance of multiple pathogen detections.

275 and Table 5: “For antibiotic modification, were other factors considered, for example modifications based on clinical improvement/deterioration? Consider adding a flowchart to clarify escalation and de-escalation that different patient groups underwent. Ideally the flow chart would include information on the type of result that prompted the modification, or a categorical evaluation on whether the modification was truly warranted based on the result, and whether there had been results, that would have warranted escalation or de-escalation, but did not result in changes in patient management.”

Response: We appreciate the reviewer’s thoughtful and constructive suggestion. We agree that a detailed evaluation of the clinical factors influencing antibiotic modification, including whether changes were prompted by microbiological results, clinical improvement or deterioration, or other clinical considerations, would provide additional insight into antimicrobial decision-making.

However, the present study was designed to compare antibiotic modifications between the pre-implementation and FilmArray periods rather than to adjudicate the clinical appropriateness of individual treatment decisions. Because of the retrospective study design, it was not possible to reliably determine the relative contribution of microbiological results versus other clinical factors to each antibiotic modification, nor to objectively assess whether changes were warranted or whether opportunities for escalation or de-escalation were missed.

We agree that a flowchart incorporating these clinical decision pathways would be valuable. However, such an analysis was beyond the scope of the current study and would require detailed prospective clinical adjudication. We have acknowledged this as a limitation in the revised manuscript and consider it an important area for future research.

“The comparison of the demographic details between the study groups is presented in Supplementary file 3. As this is important information consider transferring it to the main article.”

Response: We agree that comparison of baseline demographic characteristics is essential for interpreting the clinical-impact analyses. Accordingly, the table previously provided as Supplementary File 3 has been moved into the main manuscript and is now presented as Table 4.

To improve clarity throughout the manuscript, we have also revised the terminology used to describe the study groups. The previous labels, "sputum culture group" and "FilmArray group," have been replaced with "pre-implementation period" and "FilmArray period," respectively. These revised terms more accurately reflect the study design because both sputum and bronchoalveolar lavage specimens were included during the FilmArray period, whereas the previous terminology could incorrectly suggest that only sputum specimens were analyzed.

DISCUSSION

318-319 “Consulting the list of bacteria for this group shows that most of them appear to be true pathogens. Please clarify, what do the authors mean by this phrase?”

Response: We thank the reviewer for pointing out this ambiguity. The original wording was imprecise. By this phrase, we intended to indicate that the bacteria detected by the FilmArray Pneumonia Panel in this group were organisms that are commonly recognized as respiratory pathogens, rather than microorganisms that are typically regarded as colonizers or contaminants. We have revised the text to clarify this point and to avoid overinterpretation of the results.

319 ”In THE systematic review..”, I believe should be: ”in A systematic review”

Response: Thank you for this helpful suggestion. The manuscript has been revised accordingly.

327 “Could the authors refer here to surveillance data from their region?”

Response: We have incorporated regional antimicrobial resistance surveillance data from Gyeongsang National University Changwon Hospital collected during the same study period (2023) to provide local epidemiological context for ESBL-producing Enterobacterales, carbapenem-resistant Enterobacterales, and methicillin-resistant Staphylococcus aureus. The Discussion has been revised accordingly.

331 “Consider discussing the possibility of colonization, thus leading to unnecessary antibiotic escalation?”

Response: We thank the reviewer for this valuable suggestion. We agree that the high analytical sensitivity of the FilmArray Pneumonia Panel may detect colonizing organisms that are not responsible for the patient’s infection. If these findings are interpreted without appropriate clinical correlation, they could potentially lead to unnecessary initiation or escalation of antimicrobial therapy. We have therefore revised the Discussion to explicitly acknowledge this limitation and to emphasize that FilmArray results should always be interpreted together with the patient’s clinical presentation, radiologic findings, and conventional microbiological results.

337-339: “Consider discussing also what are the advantages of culture in comparison with molecular methods? Such as semi-quantitative nature, scope of detectable bacteria (and fungi) more comprehensive than in a targeted panel, possibilities for selective cultures and phenotypic susceptibility testing?”

Response: We fully agree that the complementary strengths of conventional culture should be more clearly acknowledged.

Accordingly, the Discussion now emphasizes that, despite the rapid turnaround time of the FilmArray Pneumonia Panel, conventional culture remains indispensable because it provides semi-quantitative information that may help distinguish colonization from infection, detects a broader range of bacterial and fungal pathogens than those included in targeted molecular panels, enables selective culture for unexpected organisms, and supports phenotypic antimicrobial susceptibility testing. We therefore emphasize that molecular assays should be regarded as complementary to, rather than replacements for, conventional microbiological methods.

356-357 ”several studies”, but only one reference, consider adding other relevant references or rephrasing.

Response: We changed the expression, ‘several studies’ into ‘a study’.

362-364 “seems to contradict with 374-376, so please specify how you define the difference between antibiotic optimization and antibiotic modification.”

Response: We agree that the term "antibiotic optimization"was too broad and could be interpreted inconsistently with "antibiotic modification."To avoid ambiguity, we have removed the term "antibiotic optimization". Instead, Table 5 and the corresponding text now explicitly distinguish "result-guided initiation of antibiotics"from "modification of an ongoing antibiotic regimen (including escalation and de-escalation)." We believe these revisions provide a clearer description of the clinical actions evaluated in this study.

366 “I believe it would be important to evaluate whether the Filmarray results even would consistently enough warrant de-escalation to have a detectable effect on antibiotic prescription patterns? Ie if the Filmarray result is, for example polymicrobial (as it often is), it seems natural that the clinician would await for culture results and/or clinical improvement, before de-escalation. The authors could explore already in the results section, what is the proportion of truly actionable results in a real life scenario.”

Response: We greatly appreciate this thoughtful and clinically important suggestion because it prompted us to examine an aspect of clinical utility that had not been sufficiently explored in the original manuscript.

We agree that not every positive FilmArray result is immediately actionable, particularly in patients with polymicrobial detections or when confirmation by conventional culture and correlation with the clinical presentation are required before narrowing antimicrobial therapy.

In our study, antibiotic management reflected real-world clinical practice rather than hypothetical treatment decisions. Clinicians interpreted FilmArray results together with patients' clinical status, radiologic findings, severity of illness, and subsequent culture results when making antimicrobial decisions. Consequently, not every positive FilmArray result led to immediate antibiotic de-escalation.

Because this was a retrospective study, we could not reliably classify individual FilmArray results as "actionable" or "non-actionable" using predefined criteria without introducing subjective interpretation. We therefore acknowledge this as an important limitation and recommend that future prospective studies evaluate both the proportion of actionable FilmArray results and their direct impact on antimicrobial stewardship interventions.

To address the reviewer’s concern, we have expanded the Discussion to clarify that the clinical utility of the FilmArray Pneumonia Panel depends not only on its diagnostic performance but also on the interpretability and clinical actionability of individual test results. We also emphasize that polymicrobial detections and discordant findings may appropriately prompt clinicians to continue empirical therapy until additional microbiological or clinical information becomes available. Consequently, the observed impact on antibiotic prescribing reflects real-world clinical decision-making rather than the diagnostic performance of the assay alone.

410 ”Lack of reliability”, can the authors be more clear; does this refer to the clinicians lack of confidence in the results (if so, how was this evaluated or measured)? Or does it refer to the fact, that the absence of the genes included in the panel does not necessarily reflect phenotypic susceptibility?

Response: We thank the reviewer for pointing out this ambiguity. Our intention was not to suggest that clinicians lacked confidence in the FilmArray results, as clinician confidence or perceptions were not evaluated in this retrospective study. To avoid misinterpretation, we have removed the phrase "lack of reliability" and replaced it with a more precise description that accurately reflects the scope of the present study. The revised wording now emphasizes the limitations of molecular resistance-marker detection rather than clinician confidence in the assay.

414-415 “Can this statement be made with confidence? Is it shown in the article, that pathogens responsible for the clinical disease or AMR genes were missed by culture, especially when correlation with culture based susceptibility testing or culture based AMR screening was not included in the study? Consider revising to better reflect the actual results of the current study.”

Response: We thank the reviewer for this important observation and agree that our original wording was stronger than warranted by the available evidence.

Our study did not directly evaluate whether pathogens responsible for the clinical infection or antimicrobial resistance determinants were missed by conventional culture. In addition, correlation between FilmArray resistance gene detection and culture-based antimicrobial susceptibility testing or comprehensive resistance mechanism analysis was beyond the scope of this study.

Accordingly, we have revised the Discussion to avoid implying that the FilmArray Pneumonia Panel definitively identified pathogens or resistance determinants missed by conventional culture. Instead, we now state that the FilmArray Pneumonia Panel may provide complementary microbiological information that can assist conventional diagnostic methods, while emphasizing that its clinical significance should always be interpreted together with conventional culture findings and the patient’s clinical presentation.

We believe that the revised wording more accurately reflects both the findings and the limitations of the present study.

“Because of the retrospective nature of the study, the differences observed between the timeframe before Filmarray use and during the Filmarray use may be due to various factors unrelated to the newly adopted methodology. It is mentioned briefly in the manuscript (principally in 354-355, 390-392), but I believe it warrants to be acknowledged as a significant limitation even more clearly.”

Response: We thank the reviewer for this important comment and fully agree that the retrospective before-and-after study design represents one of the major limitations of the present study.

Because the pre-implementation period and the FilmArray implementation period occurred at different time points, temporal changes unrelated to the introduction of the FilmArray Pneumonia Panel may have influenced antibiotic prescribing practices and clinical outcomes. Potential sources of confounding include changes in antimicrobial stewardship activities, institutional treatment protocols, clinician experience, patient characteristics, and local epidemiology.

Accordingly, we have substantially expanded the Limitations section to emphasize that the observed differences between the two study periods cannot be attributed solely to implementation of the FilmArray Pneumonia Panel. We further state that prospective controlled studies with concurrent comparison groups are required to more accurately determine the causal impact of the FilmArray Pneumonia Panel on antimicrobial prescribing and patient outcomes.

“Some inherent limitations of molecular methods, that I believe could be mentioned or discussed in relation to the results:

-Discussion of clinically insignificant findings due to high sensitivity of the assay, and the risks related to this.

-Scalability of the methodology at its current price point?”

Response: We agree that these are important considerations when interpreting the clinical utility of multiplex molecular diagnostic assays.

Accordingly, we have expanded the Discussion to acknowledge two additional limitations of the FilmArray Pneumonia Panel.

First, because of its high analytical sensitivity, the assay may detect colonizing organisms, nonviable microorganisms, or clinically insignificant pathogens that are not responsible for the patient’s infection. Therefore, FilmArray results should always be interpreted in conjunction with the patient’s clinical presentation, radiologic findings, and conventional microbiological results to avoid unnecessary antimicrobial therapy.

Second, we acknowledge that the relatively high cost of multiplex molecular testing may limit its widespread implementation, particularly in resource-limited healthcare settings. Although earlier diagnosis and more appropriate antimicrobial therapy may partially offset these additional costs, formal cost-effectiveness analyses were beyond the scope of the present study. Future prospective studies should evaluate both the economic impact and the scalability of routine FilmArray implementation in different clinical settings.

These additional considerations have been incorporated into the revised Discussion.

Discussion, line 366 — Proportion of truly actionable results

"...The authors could explore already in the results section, what is the proportion of truly actionable results in a real life scenario."

Response: We greatly appreciate this suggestion because it prompted us to examine an important aspect of clinical utility that had not been sufficiently addressed in the original manuscript.

The new Table 6 now directly addresses this question by reporting both the proportion of microbiological results that led to an antibiotic action (26.5% vs. 40.5%) and the specific type of action (result-guided initiation, escalation, or de-escalation).

Importantly, de-escalation occurred in only 8 of 81 patients in the FilmArray period, supporting the reviewer’s hypothesis that polymicrobial molecular results alone rarely provide clinicians with sufficient confidence to narrow antimicrobial therapy. This finding is now discussed explicitly in the revised Discussion, where we emphasize that the clinical value of the FilmArray Pneumonia Panel lies not only in its diagnostic accuracy but also in how its results are integrated into real-world antimicrobial decision-making.

Summary of changes to the manuscript (statistical sections)

• The Statistical Analysis section was completely rewritten to include pre-specified operational definitions, normality assessment, parallel reporting of parametric and non-parametric analyses, multivariable regression models with predefined covariates, IPTW sensitivity analyses, handling of deaths and outcome truncation, statistical software, and explicit adherence to the STROBE and STARD reporting guidelines.

• Table 4 (Baseline characteristics) was moved from the Supplementary Material to the main manuscript. Standardized mean differences were added, and one implausible body mass index value (658.8 kg/m²) was corrected. The revised BMI values are 21.87 ± 3.88 kg/m² and 21.63 ± 4.16 kg/m².

• Table 5 (Clinical outcomes) was completely reconstructed to report the analysis population and sample size for each outcome, mean ± SD and median (IQR), adjusted effect estimates with 95% confidence intervals, and 30-day mortality.

• Table 6 (Result-driven antibiotic actions) was newly structured to present the frequency and pattern of antibiotic actions (result-guided initiation, escalation, and de-escalation), together with explicit denominators and Fisher’s exact test results.

• The Abstract and Conclusions were revised to reflect the corrected analyses and updated interpretation of the study findings.

• A de-identified analytic dataset together with the complete statistical analysis script has been deposited in a public repository to ensure transparency and reproducibility.

Attachment Submitted filename: Response_to_Reviewers.docx

# Peer review: Decision Letter 1

**DOI:** 10.1371/journal.pone.0358986.r003

**Authors:** Flora De Conto

25 Aug 2026

Dear Dr. Kim,

Thank you for submitting your manuscript to PLOS One. After careful consideration, we feel that it has merit but does not fully meet PLOS One’s publication criteria as it currently stands. Therefore, we invite you to submit a revised version of the manuscript that addresses the points raised during the review process.

Please submit your revised manuscript by Oct 09 2026 11:59PM. If you will need more time than this to complete your revisions, please reply to this message or contact the journal office at plosone@plos.org. When you're ready to submit your revision, log on to https://www.editorialmanager.com/pone/ and select the 'Submissions Needing Revision' folder to locate your manuscript file.

- A letter that responds to each point raised by the academic editor and reviewer(s). You should upload this letter as a separate file labeled 'Response to Reviewers'.
- A marked-up copy of your manuscript that highlights changes made to the original version. You should upload this as a separate file labeled 'Revised Manuscript with Track Changes'.
- An unmarked version of your revised paper without tracked changes. You should upload this as a separate file labeled 'Manuscript'.

If you would like to make changes to your financial disclosure, please include your updated statement in your cover letter. Guidelines for resubmitting your figure files are available below the reviewer comments at the end of this letter.

If applicable, we recommend that you deposit your laboratory protocols in protocols.io to enhance the reproducibility of your results. Protocols.io assigns your protocol its own identifier (DOI) so that it can be cited independently in the future. For instructions see: https://journals.plos.org/plosone/s/submission-guidelines#loc-laboratory-protocols. Additionally, PLOS One offers an option for publishing peer-reviewed Lab Protocol articles, which describe protocols hosted on protocols.io. Read more information on sharing protocols at https://plos.org/protocols?utm_medium=editorial-email&utm_source=authorletters&utm_campaign=protocols.

As the corresponding author, your ORCID iD is verified in the submission system and will appear in the published article. PLOS supports the use of ORCID, and we encourage all coauthors to register for an ORCID iD and use it as well. Please encourage your coauthors to verify their ORCID iD within the submission system before final acceptance, as unverified ORCID iDs will not appear in the published article. *Only*  the individual author can complete the verification step; PLOS staff *cannot*  verify ORCID iDs on behalf of authors.

We look forward to receiving your revised manuscript.

Kind regards,

Flora De Conto, Ph.D.

Academic Editor

PLOS One

Journal Requirements:

If the reviewer comments include a recommendation to cite specific previously published works, please review and evaluate these publications to determine whether they are relevant and should be cited. There is no requirement to cite these works unless the editor has indicated otherwise.

Please review your reference list to ensure that it is complete and correct. If you have cited papers that have been retracted, please include the rationale for doing so in the manuscript text, or remove these references and replace them with relevant current references. Any changes to the reference list should be mentioned in the rebuttal letter that accompanies your revised manuscript. If you need to cite a retracted article, indicate the article’s retracted status in the References list and also include a citation and full reference for the retraction notice.

Additional Editor Comments:

I believe that the revision of the article has significantly improved its content and appeal.

The second (minor) revision request is intended to make it even more impactful.

[Note: HTML markup is below. Please do not edit.]

Reviewers' comments:

Reviewer’s Responses to Questions

**Comments to the Author**

Reviewer #3: (No Response)

**********

2. Is the manuscript technically sound, and do the data support the conclusions?

Reviewer #3: Yes

**********

3. Has the statistical analysis been performed appropriately and rigorously?

Reviewer #3: I Don't Know

**********

4. Have the authors made all data underlying the findings in their manuscript fully available?

The PLOS Data policy

Reviewer #3: Yes

**********

5. Is the manuscript presented in an intelligible fashion and written in standard English?

Reviewer #3: Yes

**********

Reviewer #3: I would like to thank the Editor, the other Reviewers and the Authors for thorough work on this article. Also, I would like to congratulate the authors on the comprehensive corrections in the revised version and the improvements made in the article. However, as the changes made are quite extensive, there are some novel points I would like to raise for consideration to further improve the current version of the article. The two comments listed first here, I believe to be most pertinent, and comments 3-6 are mostly minor in nature.

1) One of the previous comments, concerning the time to result driven action, prompted extensive revision. I would like to add, that an additional important comparison might in fact be made comparing time from sampling to result driven decision: the main advantage of molecular testing generally being faster turn-around time. Thus, an important additional correction would be to report also time from sampling to result driven decision for both groups (pre-intervention period and FilmArray period) and use terminology that describes this clearly. For clarity, I believe the current comparison of time from microbiologic result to action should also remain in the article.

2) Table 6:

The authors have explored the proportions of different types of actions in a subgroup of patients where some active action after microbiologic result was undertaken. I believe it would be more meaningful and informative to report the proportion of patients with specific modifications of all patients included in the clinical impact evaluation (that is, include in the denominator also the patients, where no modification was made). Deciding not to change the antibiotic regimen after the microbiological result is still a clinical decision even if it appears "passive". At the very minimum, a row indicating how many patients in each period did not have changes in the antibiotic regimen, should be added. But to demonstrate the possible difference seen with a different approach to the dataset, please find below as an example the proportions calculated from the total group included in the clinical comparison:

pre-FilmArray period

initiation: 10/294, 3.4%

escalation: 44/294, 14.9%

de-escalation: 24/294, 8.1%

FilmArray period

initiation: 55/200, 27.5%

escalation: 18/200, 9%

de escalation: 8/200, 4%

Crudely, both de-escalation and escalation appear to be more frequent in the pre-intervention period, (possible statistical significance?). Please, consider this carefully and if you decide to maintain the initial approach, please provide justification in the article. I acknowledge that exploring the suggested approach might result in the need to revise the conclusions drawn and the discussion section, but I believe it to be an important enough question to warrant careful revision.

Detailed minor revisions:

3) Abstract, lines 47-48: I recommend removing the indirect (“double-negative”) expression: “did not decrease” and substituting with more direct language: “increased antibiotic exposure”.

4) Abstract lines 49-50: I suggest adding something to further clarify the role of molecular testing, for example: “when used as a complementary tool to traditional microbiological methods and integrated with structured ...”

5) Table 5: In the 30-day mortality comparison both groups are missing one patient. Based on Figure S1 this is missing data. Can the authors confirm whether this is correct? Missing data on 30-mortality is of course possible, but in small datasets (about 200 patients) usually rather rare (i.e. usually it is apparent whether patients are alive or not at the 30-day time point).

6) Figure S1: Pre-implementation box only mentions sputum culture, where I believe, should be also culture of BAL-samples.

**********

what does this mean?). If published, this will include your full peer review and any attached files.

If you choose “no”, your identity will remain anonymous but your review may still be made public.

**Do you want your identity to be public for this peer review?**  For information about this choice, including consent withdrawal, please see our Privacy Policy

Reviewer #3: **Yes:** Maarit Johanna Ahava

**********

[NOTE: If reviewer comments were submitted as an attachment file, they will be attached to this email and accessible via the submission site. Please log into your account, locate the manuscript record, and check for the action link "View Attachments". If this link does not appear, there are no attachment files.]

To ensure your figures meet our technical requirements, please review our figure guidelines: https://journals.plos.org/plosone/s/figures

You may also use PLOS’s free figure tool, NAAS, to help you prepare publication quality figures: https://journals.plos.org/plosone/s/figures#loc-tools-for-figure-preparation.

NAAS will assess whether your figures meet our technical requirements by comparing each figure against our figure specifications.

Attachment Submitted filename: Plos one filmarray Reviewer 3 23.8.2026.pdf

# Author comment: Author response to Decision Letter 2

**DOI:** 10.1371/journal.pone.0358986.r004

6 Sep 2026

Response to Reviewers — second revision

PONE-D-26-13230R1

Diagnostic Performance and Clinical Impact of the BioFire FilmArray Pneumonia Panel Compared with Conventional Culture in Hospitalized Patients with Pneumonia

General response

We thank Reviewer #3 for a second careful reading and for the generous assessment of our first revision. The two principal comments have again improved the paper substantially, and both have been fully addressed in the revised manuscript.

Comment 1 highlighted an important aspect that was not adequately addressed in our previous revision. Turnaround time is the central practical advantage of syndromic molecular testing, and our previous analysis, anchored entirely on the reporting of the result, could not show it.

Comment 2 identified an equally important framing problem: by restricting Table 6 to patients in whom some action followed the result, we had treated a deliberate decision not to change therapy as though it were not a decision at all. Both tables have been rebuilt. Measured from specimen sampling, the difference was large and statistically significant, and this finding is now emphasized in the revised manuscript.

We are glad to report that the reviewer’s own calculations were reproduced by our re-analysis, and that the reviewer’s substantive question about escalation and de-escalation has a clear answer, given below.

The reviewer’s minor points 3 to 6 have all been addressed, and one of them (point 6) prompted a correction to the supplementary figure that we are grateful to have caught before publication.

Reviewer #3

Comment 1 — Time from sampling to result-driven action

"...an additional important comparison might in fact be made comparing time from sampling to result driven decision: the main advantage of molecular testing generally being faster turn-around time. Thus, an important additional correction would be to report also time from sampling to result driven decision for both groups... For clarity, I believe the current comparison of time from microbiologic result to action should also remain in the article."

Response: We agree, and this comment has changed the emphasis of the paper. Two new rows have been added to Table 5, and the existing result-reporting comparison has been retained exactly as the reviewer requests, so that the two timings can be read side by side.

First, we now report the turnaround time itself — the interval from obtaining the respiratory specimen to reporting of the final microbiological result:

— Pre-implementation period: mean 3.18 ± 1.21 days, median 3 (IQR 2–4), n = 293

— FilmArray period: mean 0.02 ± 0.12 days, median 0 (IQR 0–0), n = 199

— Difference −3.17 days (95% CI −3.31 to −3.03); P < 0.001

Second, we report the interval from sampling to the result-driven antibiotic action:

— Pre-implementation period: mean 3.91 ± 1.73 days, median 4 (IQR 2–5), n = 78

— FilmArray period: mean 0.47 ± 1.23 days, median 0 (IQR 0–0), n = 81

— Unadjusted difference −3.44 days (95% CI −3.91 to −2.97); adjusted difference −3.53 days (95% CI −4.08 to −2.99); P < 0.001

The contrast between the two timings is itself informative, and we now make it explicitly in the Discussion. Measured from the reporting of the result, the panel advanced antibiotic decisions by about half a day; measured from the moment the specimen was taken, it advanced them by three and a half days.

The terminology has been made explicit throughout: "turnaround time" for sampling to reporting, "time from sampling to antibiotic action", and "time from result reporting to antibiotic action". Most of the observed time advantage therefore appears to derive from the shorter diagnostic turnaround time rather than from a faster clinician response after the result became available.

Turnaround time is presented unadjusted, since it is a property of the diagnostic method rather than of the patient; the two action-time outcomes are presented both unadjusted and adjusted.

Comment 2 — Denominator for Table 6

"...I believe it would be more meaningful and informative to report the proportion of patients with specific modifications of all patients included in the clinical impact evaluation... Deciding not to change the antibiotic regimen after the microbiological result is still a clinical decision even if it appears ‘passive’... Crudely, both de-escalation and escalation appear to be more frequent in the pre-intervention period, (possible statistical significance?)"

Response: We accept this point without reservation. Table 6 has been rebuilt on the whole clinical-impact cohort, and a row for patients in whom no action followed the result has been added. The reviewer’s calculations were confirmed by our re-analysis, with minor differences due only to rounding. The revised Table 6 is as follows (percentages are of 294 and 200 patients respectively):

— Result-guided initiation: 10 (3.4%) vs 55 (27.5%); P < 0.001; adjusted OR 9.20 (95% CI 4.48–18.90), P < 0.001

— Escalation: 44 (15.0%) vs 18 (9.0%); P = 0.053; adjusted OR 0.55 (95% CI 0.29–1.03), P = 0.061

— De-escalation: 24 (8.2%) vs 8 (4.0%); P = 0.092; adjusted OR 0.58 (95% CI 0.24–1.40), P = 0.226

— No action attributable to the result: 216 (73.5%) vs 119 (59.5%); P = 0.001; adjusted OR 0.54 (95% CI 0.36–0.81), P = 0.003

The four-category distribution differed between periods (chi-square P < 0.001).

On the reviewer’s specific question — whether escalation and de-escalation were significantly more frequent before implementation — the answer is that the direction is as the reviewer observed, but neither difference reaches conventional significance, before or after adjustment. We now state this explicitly rather than leaving it to be inferred.

This whole-cohort analysis provides a more clinically informative interpretation of the pattern of antibiotic actions, and we have revised the Results and Discussion accordingly. What changed with implementation of the panel was not the frequency of escalation or de-escalation but the character of the decision: result-guided antibiotic initiation was substantially more frequent among patients who were not receiving antibiotics at the time of microbiological reporting (3.4% vs. 27.5%), and the proportion of patients in whom the microbiological result changed nothing fell from 73.5% to 59.5%. At the same time, the fact that 59.5% of patients in the FilmArray period had no antibiotic action attributable to the microbiological result within five days, and that de-escalation remained uncommon in both periods, is now presented as a genuine limitation of real-world actionability rather than as a neutral observation. This is consistent with our observation of modestly increased total antibiotic exposure that we report, and with the randomized and observational literature we cite.

Comment 3 — Abstract wording

"Abstract, lines 47-48: I recommend removing the indirect (“double-negative”) expression: “did not decrease” and substituting with more direct language: “increased antibiotic exposure”."

Response: Changed as suggested. The sentence now reads:

"…However, total antibiotic exposure was modestly longer (adjusted mean difference, 1.86 days; 95% CI, 0.29–3.44; P = 0.021), and antibiotic de-escalation rates did not differ significantly between the two periods."

Comment 4 — Role of molecular testing in the Abstract

"Abstract lines 49-50: I suggest adding something to further clarify the role of molecular testing, for example: “when used as a complementary tool to traditional microbiological methods and integrated with structured ...”"

Response: Adopted. The concluding sentence of the Abstract now reads:

"The BioFire FilmArray Pneumonia Panel improved pathogen detection and facilitated earlier and more frequent microbiology-guided antibiotic management, and was associated with a modest reduction in hospital length of stay. However, it modestly increased overall antibiotic exposure and did not increase antibiotic de-escalation. Rapid molecular diagnostics may improve the timeliness and precision of antimicrobial therapy, but their full clinical benefit is likely to be realized only when they are used as a complementary tool alongside conventional microbiological methods and integrated within a structured antimicrobial stewardship program."

Comment 5 — Missing 30-day mortality data

"Table 5: In the 30-day mortality comparison both groups are missing one patient. Based on Figure S1 this is missing data. Can the authors confirm whether this is correct?"

Response: The reviewer is correct, and we thank them for checking. Vital status at 30 days could not be ascertained in exactly one patient in each period. In both cases the patient was transferred to another institution before day 30 and no further record was available to us. The reviewer is also right that this should be rare, and it is: 2 of 494 patients (0.4%).

We have added a footnote to Table 5 stating the reason, and the lower panel of S1 Fig now labels these two patients explicitly. The mortality comparison is therefore based on 293 and 199 patients. Assigning both patients to either outcome does not change the conclusion (all P > 0.1).

Comment 6 — S1 Fig: culture of BAL samples

"Figure S1: Pre-implementation box only mentions sputum culture, where I believe, should be also culture of BAL-samples."

Response: The reviewer is right and we are grateful for the correction. Bronchoalveolar lavage cultures were indeed included in the pre-implementation period; the label in S1 Fig was also inconsistent with the manuscript text, which refers throughout to conventional culture rather than to sputum culture alone. S1 Fig has been corrected: the period is now labelled "conventional culture" and the screening box reads "Patients with a respiratory culture (sputum or BAL) for suspected pneumonia". We have re-checked the remaining figure labels for the same error and found none.

Summary of changes

— Table 5: two new rows reporting turnaround time (sampling to result reporting) and time from sampling to antibiotic action, with the existing result-reporting comparison retained; footnote added on the two patients with unascertained 30-day vital status.

— Table 6: rebuilt on the whole clinical-impact cohort (294 and 200), with a row for patients in whom no action followed the result, Fisher exact P values, and adjusted odds ratios.

— Abstract: direct wording for the antibiotic-exposure finding; sampling-to-decision interval added; concluding sentence revised to describe the panel as a complementary tool within a stewardship program.

— Results and Discussion: revised to distinguish turnaround time from clinician response time, and to interpret the whole-cohort distribution of antibiotic actions, including the absence of a significant difference in escalation and de-escalation.

— S1 Fig: pre-implementation period relabelled as conventional culture of sputum or BAL; turnaround time added to the analysis-population panel.

— Statistical Analysis: definitions of the two sampling-based intervals added.

Attachment Submitted filename: Response_to_Reviewers_R2.docx

# Editor report: Decision Letter 2

**DOI:** 10.1371/journal.pone.0358986.r005

**Authors:** Flora De Conto

8 Sep 2026

Diagnostic Performance and Clinical Impact of the BioFire FilmArray Pneumonia Panel Compared with Conventional Culture in Hospitalized Patients with Pneumonia

PONE-D-26-13230R2

Dear Dr. Kim,

We’re pleased to inform you that your manuscript has been judged scientifically suitable for publication and will be formally accepted for publication once it meets all outstanding technical requirements.

Within one week, you’ll receive an e-mail detailing the required amendments. When these have been addressed, you’ll receive a formal acceptance letter and your manuscript will be scheduled for publication.

An invoice will be generated when your article is formally accepted. Please note, if your institution has a publishing partnership with PLOS and your article meets the relevant criteria, all or part of your publication costs will be covered. Please make sure your user information is up-to-date by logging into Editorial Manager at Editorial Manager® and clicking the ‘Update My Information' link at the top of the page. For questions related to billing, please contact billing support.

If your institution or institutions have a press office, please notify them about your upcoming paper to help maximize its impact. If they’ll be preparing press materials, please inform our press team as soon as possible -- no later than 48 hours after receiving the formal acceptance. Your manuscript will remain under strict press embargo until 2 pm Eastern Time on the date of publication. For more information, please contact onepress@plos.org.

Kind regards,

Flora De Conto, Ph.D.

Academic Editor

PLOS One

Additional Editor Comments (optional):

The changes requested as part of the second revision were accepted with a favorable impact on the content and impact of the article.

Reviewers' comments:

# Editor report: Acceptance letter

**DOI:** 10.1371/journal.pone.0358986.r006

**Authors:** Flora De Conto

PONE-D-26-13230R2

PLOS One

Dear Dr. Kim,

I'm pleased to inform you that your manuscript has been deemed suitable for publication in PLOS One. Congratulations! Your manuscript is now being handed over to our production team.

At this stage, our production department will prepare your paper for publication. This includes ensuring the following:

* All references, tables, and figures are properly cited

* All relevant supporting information is included in the manuscript submission,

* There are no issues that prevent the paper from being properly typeset

You will receive further instructions from the production team, including instructions on how to review your proof when it is ready. Please keep in mind that we are working through a large volume of accepted articles, so please give us a few days to review your paper and let you know the next and final steps.

Lastly, if your institution or institutions have a press office, please let them know about your upcoming paper now to help maximize its impact. If they'll be preparing press materials, please inform our press team within the next 48 hours. Your manuscript will remain under strict press embargo until 2 pm Eastern Time on the date of publication. For more information, please contact onepress@plos.org.

You will receive an invoice from PLOS for your publication fee after your manuscript has reached the completed accept phase. If you receive an email requesting payment before acceptance or for any other service, this may be a phishing scheme. Learn how to identify phishing emails and protect your accounts at https://explore.plos.org/phishing.

If we can help with anything else, please email us at customercare@plos.org.

Thank you for submitting your work to PLOS One and supporting open access.

Kind regards,

PLOS One Editorial Office Staff

on behalf of

Prof. Flora De Conto

Academic Editor

PLOS One
